# Correction: Prevalence and factors associated with undernutrition among 15–49-year-old women in Sierra Leone: A secondary data analysis of Sierra Leone Demographic Health Survey of 2019

**DOI:** 10.1371/journal.pone.0330515

**Published:** 2025-08-18

**Authors:** The PLOS One Staff

The image for Fig 2 is incorrect. The figure caption appear in the correct order. The authors have provided a corrected version of figures here.

**Fig 2:**
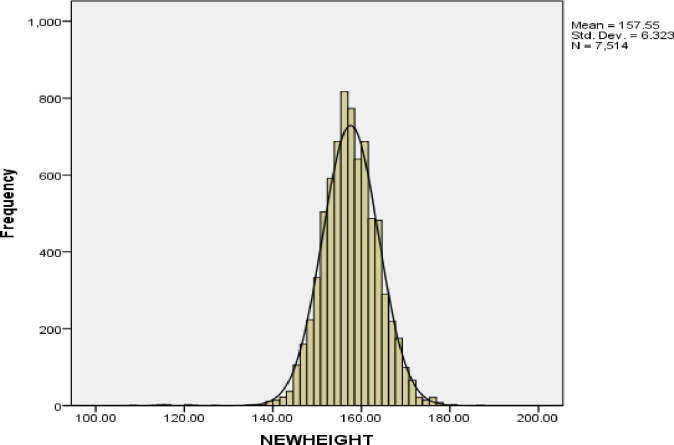


The publisher apologizes for the error.
