# Supplementary material for: Prevalence and factors associated with undernutrition among 15–49-year-old women in Sierra Leone: A secondary data analysis of Sierra Leone Demographic Health Survey of 2019
Source: PLoS One. 2024 Nov 11;19(11):e0311845. doi: 10.1371/journal.pone.0311845 (PMC11554195; doi:10.1371/journal.pone.0311845)
Supplement: Attachment — Submitted filename: review by Gxxx.pdf [file pone.0311845.s001.pdf]

PLOS ONE

Prevalence and factors associated with undernutrition among 15–49-year-old women in Sierra Leone: A secondary data analysis of Sierra Leone Demographic Health Survey of 2019.

--Manuscript Draft--

**Manuscript Number:**	PONE-D-23-29763R1
**Article Type:**	Research Article
**Full Title:**	Prevalence and factors associated with undernutrition among 15–49-year-old women in Sierra Leone: A secondary data analysis of Sierra Leone Demographic Health Survey of 2019.
**Short Title:**	Prevalence and factors for undernutrition among women in the reproductive age (15-49 years) in Sierra Leone.
**Corresponding Author:**	David Lagoro Kitara, MD, MMed, FCS, PhD<br>Gulu University Faculty of Medicine<br>Gulu, Uganda UGANDA
**Keywords:**	Keywords: Underweight, stunting, women of reproductive age (15-49 years), Sierra Leone, undernutrition.
**Abstract:**	Background: Undernutrition of women of childbearing age is pertinent for maternal and offspring health. This study aimed to determine the prevalence and factors associated with undernutrition (underweight and stunting) among women of reproductive age (15-49 years) in Sierra Leone using secondary data analysis of the 2019 Demographic Health Survey.<br>Methods: Anthropometric measurements and maternal characteristics were obtained from the Sierra Leone Demographic Health Survey (SLDHS) of 2019. The heights and weights of women were measured, and BMI in kg/m2 was calculated. Based on the World Health Organization's recommendations, stunting was defined as having heights <145cm and underweight as BMI <18.5kg/m2. Multivariable logistic regression analyses were conducted to identify correlates of undernutrition, with a significance level set at p<0.05.<br>Results: A total of 7,514 women of reproductive age, 15-49 years, participated in this study. The prevalence of stunting and underweight was 1.5% (113/7514) and 6.7%(502/7,514), respectively. Women with primary education had a 47% lower likelihood of stunting (adjusted Odds Ratio [aOR]=0.53, 95% Confidence Interval [CI]:0.30-0.94;p=0.029) than those with secondary education. Also, women in the poorest wealth index had a 51% lower likelihood of stunting (aOR=0.49,95%CI:0.27-0.88;p=0.017) than the middle wealth index. However, underweight was 1.48 times more likely among women with a parity of one-to-four (aOR=1.48,95% CI:1.08-2.03;p=0.015) than women who had never given birth. Additionally, underweight was 1.41 times more likely among women who listened to radios (aOR=1.41,95% CI:1.14-1.74;p=0.002) than those who did not. Notably, age groups of 15-19 years and 40-49 years had a 54% (aOR=0.46,95%CI:0.34-0.62;p<0.001) and 34% (aOR=0.66,95%CI:0.45-0.97;p=0.035) lower likelihood of underweight than 20-29-year age group, respectively. Women with primary education had a 26% lower likelihood of being underweight (aOR=0.74,95%CI:0.56-0.99;p=0.042) than those with secondary education. However, none of the wealth indices were significantly associated with underweight.<br>Conclusion: The prevalence of underweight and stunting among women of reproductive age (15-49 years) in Sierra Leone was lower compared to regional and world data. This study highlights similarities and differences in this population's prevalence and correlates of undernutrition. Underweight and stunting were less likely in women with primary education, while parity of one-to-four and listening to radios were significantly associated with underweight. Further trend studies using DHS data from 2010, 2014, and 2019 are warranted to understand the dynamics of undernutrition in Sierra Leone.
**Order of Authors:**	Nelson Onira Alema, MD, MMed, MPH
	Eric Nzirakaindi Ikoona, MD, MMed, MPH, PhD

Powered by Editorial Manager® and ProduXion Manager® from Aries Systems Corporation

	Mawe Awa Toure, MD, MSC
	Amon Njenga, MD, MPH
	John Bosco Matovu, MD, MPH
	Oliver Eleeza, MD, MSC
	Lucy Namulemo, MD
	Ronald Kaluya, MD, MPH
	Kassim Kamara, MD, MPH
	Freddy Wathum Drinkwater Oyat, MD, MPH
	Emmanuel Olal, MD, MPH
	Judith Aloyo, MD, MPH
	David Lagoro Kitara, MD, MMed, FCS, PhD

Response to Reviewers:

Response to the Academic Editor PLOS ONE.

Title: Prevalence and factors associated with undernutrition among 15–49-year-old women in Sierra Leone: A secondary data analysis of Sierra Leone Demographic Health Survey of 2019.

We want to thank this journal's Academic Editor for the reviews I received after submitting our manuscript on the above title. I have been a reviewer of PLOS ONE for many years, and I have seen the processes and have experience reviewing articles in this journal.

As per our paper presented above, this was a secondary analysis of datasets from the demographic health survey 2019 in Sierra Leone. This data collection was conducted by the Bureau of Statistics of Sierra Leone. Here is A detailed explanation of the final 7,514 respondents out of the 15,934 women.

Methods

Study design: The SLDHS-2019 was conducted as a countrywide representative cross-sectional survey led by the Bureau of Statistics of Sierra Leone (Stats SL) with technical assistance from ICF through DHS programs. This survey was funded by the United States Agency for International Development (USAID)29.

Study sites: This study was conducted in all four provinces and western areas of Sierra Leone29.

Sampling and study participants: The sampling of the study participants was based on the 2015 population and housing census of the Republic of Sierra Leone30. This census was conducted by Statistics Sierra Leone (Stats SL) and provided the ready-made sampling frame for the SLDHS-201930.

Sierra Leone is administratively divided into four provinces and western areas (urban and rural), sixteen districts, and 190 chiefdoms30,31,32. Each district is subdivided into chiefdoms/census wards, and each chiefdom/census ward is subdivided into sections30,31,32. In addition, the 2015 population and housing census subdivided each locality into convenient census: the enumeration areas (EAs)30,33. The EAs were the primary sampling units (PSUs) and clusters for the SLDHS-201930-35. The list of EAs from the 2015 census formed the basis for estimating the number of households required and classifying EAs (clusters) into urban/rural for the SLDHS-2019 sampling frame30,31,34,35.

Furthermore, the SLDHS-2019 employed a two-stage stratified sampling design, and the stratification was achieved by classifying each district into urban and rural areas34,35. So, thirty-one sampling strata were created, and samples were selected independently in each stratum via a two-stage selection process34,35.

Thus, implicit stratifications were achieved at each lower administrative level by sorting the sampling frame before sample selection according to administrative order and using a probability proportional-to-size selection during the first sampling stage34,35.

Also, five hundred and seventy-eight (578) EAs were selected using a probability proportional to EA size34,35 in the first stage of the selection process. In addition, the enumeration area size was determined by the number of households residing in it, and a household listing operation was then performed in all selected enumeration

Powered by Editorial Manager® and ProduXion Manager® from Aries Systems Corporation

areas34,35. The resulting lists of households served as a sampling frame for selecting households in the second stage of the survey34,35.
In the second stage's selection, a fixed number of twenty-four households was chosen in every cluster through an equal probability systematic sampling, resulting in a total sample size of approximately 13,872 households distributed in 578 clusters34,35. The household listing in this stage was conducted using computer tablets, and households were randomly selected through computer programming34,35.
The survey interviewed only pre-selected households in the clusters, and no replacements or changes of the selected households were allowed in the implementation stage of the survey to prevent selection bias of the study population34,35. Due to the non-proportional allocation of samples to the sixteen districts in Sierra Leone and the possible differences in response rates, sample weights were calculated, added to the data file, and applied so that the results would be representative at national and domain levels34,35. Further, because the SLDHS-2019 sample was a two-stage stratified cluster sampling, sample weights were calculated separately at each sampling stage based on sampling probabilities34,35.
After that, the SLDHS-2019 included all women aged 15-49 in the sampled households34,35. Permanent residents in the selected homes and visitors who stayed overnight before the survey were eligible for interviews in the household34,35. The man's questionnaire covered the identification of respondents, background information, reproduction, contraception, marriage and sexual activity, fertility preferences, employment status, gender roles, HIV and AIDS, and other health issues35. The biomarker questionnaire covered the identification of respondents, weights, heights, and hemoglobin measurements for children aged 0–5 years, weights, heights, HIV testing, and hemoglobin measurements for women aged 15–49 years35. The fieldworker questionnaire covered the background information on each field worker35.
Anthropometric measurements. The weight of respondents was recorded in kilograms (kg) to the nearest decimal point and was measured using an electronic scale (SECA 878)34,35. Participants' heights were measured using a stadiometer in centimeters (cm) to one decimal point34,35. Body Mass Index (BMI) of respondents was calculated in kg/m2 using weights (in kilograms) and heights (meters) of women of reproductive age (15–49 years) and classified according to WHO criteria as underweight (<18.5kg/m2), normal weight (18.5–24.9kg/m2), overweight (25.0–29.9kg/m2), obesity (≥30.0kg/m2 and ≤50.0kg/m2), and overnutrition (≥25.0kg/m2 and ≤50.0kg/m2).
Wealth Index (WI). To calculate each household's wealth, we used the wealth index (WI) as a proxy indicator of household wealth35. This composite index used household key asset ownership variables to calculate each household wealth index from the SLDHS-2019 data35. These variables were the characteristics of the household's dwelling unit, for example, the source of water, type of toilet facilities, type of fuel used for cooking, number of rooms, ownership of livestock, possessions of durable goods, mosquito nets, and primary materials for the floor, roof, and walls of the dwelling place35. The respondent's household wealth index was calculated using computer analysis of household composite factors. It was then categorized into five quintiles: poorest, poorer, middle, richer, and richest wealth indices (Table 1).
Operational definitions.
Body Mass Index (BMI): Weight in kilograms divided by heights in meters squared (kg/m2).
Underweight: BMI <18.5kg/m2
Overweight: BMI ≥25.0kg/m2 and ≤29.9kg/m2
Obese: BMI ≥30.0kg/m2 and ≤50.0kg/m2
Overnutrition (Overweight and obese): BMI ≥25.0kg/m2 and ≤50.0kg/m2.
Enumeration Area (Clusters): An EA is a geographic area consisting of a convenient number of dwelling units that serve as a counting unit for the survey.
Data Collection: Data collection for this survey was conducted from May 14, 2019, to August 31, 201929. The primary sampling unit (PSU), a cluster, was based on enumeration areas (EAs) obtained from the 2015 EA population census sampling frame29.
The SLDHS-2019 used five validated questionnaires for the thematic parts of the survey29. The household questionnaire collected data on household environment, assets, and basic demographic information of household members. The woman's questionnaire collected data on women's reproductive health, domestic violence, and nutrition indicators29. The man's questionnaire collected data on men's health, while the biomarker questionnaire collected data on anthropometry and blood tests for mothers and children (0-5 years), and the fieldworker questionnaire collected data on

Powered by Editorial Manager® and ProduXion Manager® from Aries Systems Corporation

	background information of fieldworkers36,37.<br>This secondary data analysis included women of reproductive age, 15-49 years, whose anthropometric characteristics were recorded with consent. Trained health technicians were deployed to measure the heights and weights of the participants to ensure the quality of anthropometric measurements29.<br>Out of the weighted sample of 15,934 women in the dataset, 7,514 anthropometric measurements were included in the survey design, while 8,420 had invalid weight measurements due to erroneous and ineligible measurements. The weight measurement is vital for calculating the BMI of each participant, which was finally used for assessing the nutritional status of each respondent.<br>In some of the participants' results, heights were not well recorded, and we could only obtain completed anthropometric measurements for 7,514 women who were not lactating, non-pregnant, and post-menopausal. In the final analysis, a weighted sample 7,514 was included in our secondary data analysis, as summarized in Table 1. A complete protocol with detailed explanations about data collection processes and sampling is available online29.<br>Outcome variables: The first outcome variable for this study was stunting. It was coded as "1" for stunted women and "0" for not stunted. Stunting was defined as heights of <145cm ± Standard Deviations (SD) from the median value set by the World Health Organization (WHO). The second outcome variable was underweight, which was defined as BMI<18.5kg/m2 and coded as "1" for underweight women and "0" for normal weight. Normal weight was defined as a BMI of 18.5-24.9kg/m2.<br>Independent variables: The independent variables in this study were based on previous studies, the WHO stunting framework, underweight, normal weight, and available information in the SLDHS-2019 database. We included nineteen independent variables in this data analysis.<br>Women's characteristics: Parity (categorized as para 0, para one-to-four, and five and above), work status (categorized as working-class versus not working), marital status (categorized as married versus not married/single), levels of education (categorized as no education, primary, secondary, and higher), age groups (categorized as 15-19, 20-29, 30-39, and 40-49 years), woman's stunting status (defined as heights <145cm for stunted and ≥145cm for not stunted women), and woman's BMI classification as normal BMI (18.5-24.9kg/m2) and underweight (<18.5kg/m2).<br>Household characteristics: These characteristics include regions of Sierra Leone (Northwest, Eastern, Western, Southern, and Northern); household wealth indices (categorized as richest, richer, middle, poorer, and poorest); sex of the head of household (female versus male); household size (less than six versus six and above); residency (urban versus rural); television viewing (yes versus no); reading magazines (yes versus no); listening to radios (yes versus no); smoking cigarettes (yes versus no); and alcohol use (yes versus no).<br>The study should explicitly state the criteria for "underweight" and "normal weight" to facilitate understanding of the analysis, as it investigates associations between socio-demographic characteristics and women's nutritional status without providing clear definitions for these categories.<br>In this study, underweight was determined by calculating the body mass index, which is given by the weights (kg) of respondents divided by heights in meters squared (m2) [kg/m2]. The WHO classification of the nutritional status of respondents using BMI, underweight, normal BMI, overweight, and obesity were used in this description. Underweight is described as BMI<18.5kg/m2, and Normal weight = BMI≥18.5-24.9kg/m2. This explanation has been provided for the method of this revised manuscript.<br>In Figure 1 and Figure 2, the source is mentioned as "primary data," but the text refers to the data as coming from the "2019 SLDHS," a secondary data source. This inconsistency creates confusion and should be corrected.<br>We want to acknowledge it as an error and have revised it to read, "the source of data is SLDHS-2019". We thank you for the advice.<br>Table 1 includes variables related to "work status" and "wealth indices," but the study's title references "socio-demography" without mentioning the economic aspect. The title should accurately reflect the variables included in the table.<br>We thank you for the advice. Indeed, we agree that you have revised the title to read "Socio-economic and demographic characteristics."<br>In Table 2, the "Age groups (years) 20-29" as the reference category is not justified. Providing a rationale for this selection would enhance the understanding of the analysis.

Powered by Editorial Manager® and ProduXion Manager® from Aries Systems Corporation

	Thank you for your review on this. We have looked at this issue repeatedly and are convinced that using the age group of 20-29 years as a reference category for the analysis was the right decision. This decision is because this age group had a median value, which allowed us to explore the relationship between different age groups with stunting at bi- and multivariable analysis. In the end, there was a significant relationship between the age group of 15-19 years in bivariate analysis but not multivariable regression analysis.<br>In Table 2, it is essential to specify the "reference" category for the variable "working status" to ensure the correct interpretation of the results.<br>Thank you for your review. We have noted that we erroneously left out the labeling of the reference category for this variable. We have now included it on the table. The reference category is "not working," and there were no significant relations with stunting.<br>The study should provide a more detailed and comprehensive explanation of the results and their implications in Table 2, as the current interpretation is overly brief and lacks meaningful context, making it challenging for readers to make sense of the findings.<br><br>Thank you for your reviews and advice. We have taken it up entirely and revised the manuscript by including details in the result section.<br>Addressing these critical issues is vital to improving the study's methodology and reporting's clarity, transparency, and quality. The manuscript has been rejected based on these deficiencies.<br>We thank you for critically reviewing this manuscript. Because we have provided additional information, we request that you consider re-admitting this revised manuscript for consideration for publication in your journal. We would like to have this article considered by your esteemed journal.<br><br><br>Prof. David Kitara Lagoro<br>Corresponding author
Additional Information:	
**Question**	**Response**
**Financial Disclosure**<br><br>Enter a financial disclosure statement that describes the sources of funding for the work included in this submission. Review the submission guidelines for detailed requirements. View published research articles from *PLOS ONE* for specific examples.<br><br>This statement is required for submission and **will appear in the published article** if the submission is accepted. Please make sure it is accurate.	No

**Unfunded studies**<br>Enter: *The author(s) received no specific funding for this work.*<br><br>**Funded studies**<br>Enter a statement with the following details:<br>• Initials of the authors who received each award<br>• Grant numbers awarded to each author<br>• The full name of each funder<br>• URL of each funder website<br>• Did the sponsors or funders play any role in the study design, data collection and analysis, decision to publish, or preparation of the manuscript?<br>• **NO** - Include this sentence at the end of your statement: *The funders had no role in study design, data collection and analysis, decision to publish, or preparation of the manuscript.*<br>• **YES** - Specify the role(s) played.<br><br>* typeset	
**Competing Interests**<br><br>Use the instructions below to enter a competing interest statement for this submission. On behalf of all authors, disclose any competing interests that could be perceived to bias this work—acknowledging all financial support and any other relevant financial or non-financial competing interests.<br><br>This statement is required for submission and **will appear in the published article** if the submission is accepted. Please make sure it is accurate and that any funding sources listed in your Funding Information later in the submission form are also declared in your Financial Disclosure statement.<br><br>View published research articles from *PLOS ONE* for specific examples.	All authors declare no conflict of interest

**NO authors have competing interests**<br><br>Enter: *The authors have declared that no competing interests exist.*<br><br>**Authors with competing interests**<br><br>Enter competing interest details beginning with this statement:<br><br>*I have read the journal's policy and the authors of this manuscript have the following competing interests: [insert competing interests here]*<br><br>* typeset	
**Ethics Statement**<br><br>Enter an ethics statement for this submission. This statement is required if the study involved:<br><br>• Human participants<br>• Human specimens or tissue<br>• Vertebrate animals or cephalopods<br>• Vertebrate embryos or tissues<br>• Field research<br><br>Write "N/A" if the submission does not require an ethics statement.<br><br>General guidance is provided below. Consult the submission guidelines for detailed instructions. **Make sure that all information entered here is included in the Methods section of the manuscript.**	This study on women of reproductive age (15-49 years) followed all relevant institutional guidelines and regulations. It was approved by the Sierra Leone Ethics and Scientific Review Committee (SLESRC) and the ICF Institutional Review Board. The study was conducted according to institutional guidelines where informed consent was obtained from each participant and parents and legal guardians of participants in this study.

Powered by Editorial Manager® and ProduXion Manager® from Aries Systems Corporation

**Format for specific study types**<br><br>**Human Subject Research (involving human participants and/or tissue)**<br>• Give the name of the institutional review board or ethics committee that approved the study<br>• Include the approval number and/or a statement indicating approval of this research<br>• Indicate the form of consent obtained (written/oral) or the reason that consent was not obtained (e.g. the data were analyzed anonymously)<br><br>**Animal Research (involving vertebrate animals, embryos or tissues)**<br>• Provide the name of the Institutional Animal Care and Use Committee (IACUC) or other relevant ethics board that reviewed the study protocol, and indicate whether they approved this research or granted a formal waiver of ethical approval<br>• Include an approval number if one was obtained<br>• If the study involved *non-human primates*, add *additional details* about animal welfare and steps taken to ameliorate suffering<br>• If anesthesia, euthanasia, or any kind of animal sacrifice is part of the study, include briefly which substances and/or methods were applied<br><br>**Field Research**<br><br>Include the following details if this study involves the collection of plant, animal, or other materials from a natural setting:<br>• Field permit number<br>• Name of the institution or relevant body that granted permission	
**Data Availability**<br><br>Authors are required to make all data underlying the findings described fully available, without restriction, and from the time of publication. PLOS allows rare exceptions to address legal and ethical concerns. See the PLOS Data Policy and FAQ for detailed information.	Yes - all data are fully available without restriction

A Data Availability Statement describing where the data can be found is required at submission. Your answers to this question constitute the Data Availability Statement and **will be published in the article**, if accepted.<br><br>**Important:** Stating 'data available on request from the author' is not sufficient. If your data are only available upon request, select 'No' for the first question and explain your exceptional situation in the text box.<br><br>Do the authors confirm that all data underlying the findings described in their manuscript are fully available without restriction?	
**Describe where the data may be found in full sentences. If you are copying our sample text, replace any instances of XXX with the appropriate details.**<br><br>• If the data are **held or will be held in a public repository**, include URLs, accession numbers or DOIs. If this information will only be available after acceptance, indicate this by ticking the box below. For example: *All XXX files are available from the XXX database (accession number(s) XXX, XXX.)*.<br>• If the data are all contained **within the manuscript and/or Supporting Information files**, enter the following: *All relevant data are within the manuscript and its Supporting Information files.*<br>• If neither of these applies but you are able to provide **details of access elsewhere**, with or without limitations, please do so. For example:<br><br>*Data cannot be shared publicly because of [XXX]. Data are available from the XXX Institutional Data Access / Ethics Committee (contact via XXX) for researchers who meet the criteria for access to confidential data.*<br><br>*The data underlying the results presented in the study are available from (include the name of the third party*	All relevant data are within the manuscript and its Supporting Information files.

Powered by Editorial Manager® and ProduXion Manager® from Aries Systems Corporation

and contact information or URL).<br>• This text is appropriate if the data are owned by a third party and authors do not have permission to share the data.<br><br>* typeset	
Additional data availability information:	Tick here if the URLs/accession numbers/DOIs will be available only after acceptance of the manuscript for publication so that we can ensure their inclusion before publication.

Powered by Editorial Manager® and ProduXion Manager® from Aries Systems Corporation

Manuscript Click here to access/download;Manuscript;1 Final Edited Prevalence and factors.docx

Prevalence and factors associated with undernutrition among 15–49-year-old women in Sierra Leone: A secondary data analysis of Sierra Leone Demographic Health Survey of 2019.

Nelson Onira Alema[1], Eric Nzirakaindi Ikoona[2], Mame Awa Toure[2], Oliver Eleeza[2], Amon Njenga[2], John Bosco Matovu[3] Lucy Namulemo[4,5,6], Ronald Kaluya[6], Kassim Kamara[7], Freddy Wathum Drinkwater Oyat[8], Emmanuel Olal[8,9], Judith Aloyo[8,10], David Lagoro Kitara[8,11,12*].

[1]Department of Anatomy, Faculty of Medicine, Gulu University, Gulu City, Uganda.
[2]ICAP at Columbia University, Freetown, Sierra Leone.
[3]ICAP at Columbia University, Nairobi, Kenya.
[4]Foothills Community Based interventions, Monticello, Kentucky, USA.
[5]Lindsey Wilson College, School of Professional Counseling, Kentucky, USA.
[6]Uganda Counseling and Support Services, Kampala, Uganda.
[7]Directorate of Health Security and Emergencies, Ministry of Health and Sanitation, Sierra Leone.
[8]Uganda Medical Association (UMA), UMA-Acholi branch, Gulu City, Uganda.
[9]Yotkom Medical Centre, Kitgum, Uganda.
[10]Rhites-N, Acholi, Gulu City, Uganda.
[11]Gulu Centre for Advanced Medical Diagnostics, Research, Trainings, and Innovations (GRUDI BIONTECH INITIATIVE), Gulu City, Uganda.
[*12]Gulu University, Faculty of Medicine, Department of Surgery, Gulu City, Uganda.

***Corresponding Author:** *David Lagoro Kitara is a Takemi fellow of Harvard University and Faculty at Gulu University, Faculty of Medicine, Department of Surgery, P.0. Box 166, Gulu City, Uganda, email: klagoro2@gmail.com; phone: (+256)772524474; ORCID: 0000-0001-7282-5026.*

Abstract

Background: Undernutrition of women of childbearing age is pertinent for maternal and offspring health. This study aimed to determine the prevalence and factors associated with undernutrition (underweight and stunting) among women of reproductive age (15-49 years) in Sierra Leone using secondary data analysis of the 2019 Demographic Health Survey.

Methods: Anthropometric measurements and maternal characteristics were obtained from the Sierra Leone Demographic Health Survey (SLDHS) of 2019. The heights and weights of women were measured, and BMI in kg/m^2 was calculated. Based on the World Health Organization's recommendations, *stunting* was defined as having heights <145cm and underweight as BMI <18.5kg/m^2. Multivariable logistic regression analyses were conducted to identify correlates of undernutrition, with a significance level set at p<0.05.

Results: A total of 7,514 women of reproductive age, 15-49 years, participated in this study. The prevalence of stunting and underweight was 1.5% (113/7514) and 6.7%(502/7,514), respectively. Women with primary education had a 47% lower likelihood of stunting (adjusted Odds Ratio [aOR]=0.53, 95% Confidence Interval [CI]:0.30-0.94;p=0.029) than those with secondary education. Also, women in the poorest wealth index had a 51% lower likelihood of stunting (aOR=0.49,95%CI:0.27-0.88;p=0.017) than the middle wealth index. However, underweight was 1.48 times more likely among women with a parity of one-to-four (aOR=1.48,95% CI:1.08-2.03;p=0.015) than women who had never given birth. Additionally, underweight was 1.41 times more likely among women who listened to radios (aOR=1.41,95% CI:1.14-1.74;p=0.002) than those who did not. Notably, age groups of 15-19 years and 40-49 years had a 54% (aOR=0.46,95%CI:0.34-0.62;p<0.001) and 34% (aOR=0.66,95%CI:0.45-0.97;p=0.035) lower likelihood of underweight than 20-29-year age group, respectively. Women with primary education had a 26% lower likelihood of being underweight (aOR=0.74,95%CI:0.56-0.99;p=0.042) than those with secondary education. However, none of the wealth indices were significantly associated with underweight.

Conclusion: The prevalence of underweight and stunting among women of reproductive age (15-49 years) in Sierra Leone was lower compared to regional and world data. This study highlights similarities and differences in this population's prevalence and correlates of undernutrition. Underweight and stunting were less likely in women with primary education, while parity of one-to-four and listening to radios were significantly associated with underweight. Further trend studies using DHS data from 2010, 2014, and 2019 are warranted to understand the dynamics of undernutrition in Sierra Leone.

Keywords: Underweight, stunting, women of reproductive age (15-49 years), Sierra Leone, undernutrition.

Introduction: Malnutrition, characterized by deficiencies in calories, protein, vitamins, and minerals, and poor health and social conditions, poses a significant health challenge for millions of women and adolescent girls worldwide[1]. Adequate nutrition is crucial for women's overall health and has far-reaching implications for the well-being of their children1. Children born to malnourished women are at higher risk of cognitive impairments, stunted growth, increased susceptibility to infections, and elevated morbidity and mortality rates throughout their lives[1].

Undernutrition remains a pressing global health issue, encompassing being underweight, wasting, stunting, and deficiencies in essential minerals and vitamins[2]. Research indicates that women with a body mass index (BMI) below 18.5kg/m^2 in developing countries face an escalating mortality risk and heightened vulnerability to illnesses[2-4]. Consequently, the impact of malnutrition extends beyond women's health, affecting the well-being of their children[5-6]. This scenario perpetuates a cycle of undernutrition that spans generations[5,6], especially in countries like Sierra Leone, where social and biological factors such as civil unrest, poverty, epidemic outbreaks, and food insecurity contribute to women's vulnerability to undernutrition[5]. In addition, numerous individual, household, and community factors influence women's nutritional health status[5,6].

On the one side, stunting is a consequence of complex interactions among household, environmental, socioeconomic, and cultural factors. It has detrimental effects such as susceptibility to infections, impaired cognitive and motor development, and elevated risks of non-communicable diseases later in life[7-9]. Also, research has shown that individuals who experience stunting during childhood are more likely to face challenges such as poor cognitive function, lower educational performance, reduced adult wages, decreased productivity, and an increased risk of nutrition-related chronic diseases in adulthood[10]. Therefore, ensuring adequate nutrition is a fundamental foundation for individual and population health[11-16].

Furthermore, maternal undernutrition, underweight, and stunting have been linked to adverse maternal health conditions, such as chronic energy deficiency, cesarean delivery, pre-eclampsia, anemia, decreased productivity, mental health issues, and adverse pregnancy outcomes[11-16]. On the other end of the malnutrition spectrum, overweight and obesity pose significant health risks for women, including a higher likelihood of developing hypertension, diabetes, cardiovascular diseases, and stroke[17–20].

Thus, determinants of undernutrition in women encompass many factors, including community-level water, sanitation, and hygiene (WASH) practices[21,22], food stability status[23], household income and wealth, women's education level, age at first marriage, age at first delivery, multiparity, short birth intervals, and land ownership[19,24-28]. Therefore, identifying maternal nutritional status prevalence levels and determinants is crucial for targeted interventions and resource allocation in resource-limited settings[19,21-28].

Despite the significance of understanding maternal nutritional status, limited research has been conducted in Sierra Leone, often focusing solely on malnutrition determinants in young children and adolescents. The present study addresses this research gap by investigating the risk factors for undernutrition among women of reproductive age (15-49 years) in Sierra Leone, utilizing data from the Sierra Leone Demographic Health Survey (SLDHS-2019).

The findings of this study hold essential policy implications from a global health perspective and specifically for Sierra Leone, aiding in monitoring progress toward sustainable development goals (SDGs) and regional nutrition strategies. Moreover, the study can guide the allocation of limited resources by the government and health stakeholders to improve the nutritional and health status of women and infants in Sierra Leone.

In addition, in using data from a population-based cohort of non-pregnant women of childbearing age in Sierra Leone, this study aimed to determine the prevalence and factors associated with undernutrition (underweight and stunting) among women of reproductive age (15-49 years) based on the 2019 Demographic Health Survey.

Methods

Study design: The SLDHS-2019 was conducted as a countrywide representative cross-sectional survey led by the Bureau of Statistics of Sierra Leone (Stats SL) with technical assistance from ICF through DHS programs. This survey was funded by the United States Agency for International Development (USAID)[29].

Study sites: This study was conducted in all four provinces and western areas of Sierra Leone[29].

Sampling and study participants: The sampling of the study participants was based on the 2015 population and housing census of the Republic of Sierra Leone[30]. This census was conducted by Statistics Sierra Leone (Stats SL) and provided the ready-made sampling frame for the SLDHS-2019[30].

Sierra Leone is administratively divided into four provinces and western areas (urban and rural), sixteen districts, and 190 chiefdoms[30,31,32]. Each district is subdivided into chiefdoms/census wards, and each chiefdom/census ward

is subdivided into sections[30,31,32]. In addition, the 2015 population and housing census subdivided each locality into convenient census: the enumeration areas (EAs)[30,33]. The EAs were the primary sampling units (PSUs) and clusters for the SLDHS-2019[30-35]. The list of EAs from the 2015 census formed the basis for estimating the number of households required and classifying EAs (clusters) into urban/rural for the SLDHS-2019 sampling frame[30,31,34,35].

Furthermore, the SLDHS-2019 employed a two-stage stratified sampling design, and the stratification was achieved by classifying each district into urban and rural areas[34,35]. So, thirty-one sampling strata were created, and samples were selected independently in each stratum via a two-stage selection process[34,35].

Thus, implicit stratifications were achieved at each lower administrative level by sorting the sampling frame before sample selection according to administrative order and using a probability proportional-to-size selection during the first sampling stage[34,35].

Also, five hundred and seventy-eight (578) EAs were selected using a probability proportional to EA size[34,35] in the first stage of the selection process. In addition, the enumeration area size was determined by the number of households residing in it, and a household listing operation was then performed in all selected enumeration areas[34,35]. The resulting lists of households served as a sampling frame for selecting households in the second stage of the survey[34,35].

In the second stage's selection, a fixed number of twenty-four households was chosen in every cluster through an equal probability systematic sampling, resulting in a total sample size of approximately 13,872 households distributed in 578 clusters[34,35]. The household listing in this stage was conducted using computer tablets, and households were randomly selected through computer programming[34,35].

The survey interviewed only pre-selected households in the clusters, and no replacements or changes of the selected households were allowed in the implementation stage of the survey to prevent selection bias of the study population[34,35]. Due to the non-proportional allocation of samples to the sixteen districts in Sierra Leone and the possible differences in response rates, sample weights were calculated, added to the data file, and applied so that the results would be representative at national and domain levels[34,35]. Further, because the SLDHS-2019 sample was a two-stage stratified cluster sampling, sample weights were calculated separately at each sampling stage based on sampling probabilities[34,35]. After that, the SLDHS-2019 included all women aged 15-49 in the

sampled households[34,35]. Permanent residents in the selected homes and visitors who stayed overnight before the survey were eligible for interviews in the household[34,35]. The man's questionnaire covered the identification of respondents, background information, reproduction, contraception, marriage and sexual activity, fertility preferences, employment status, gender roles, HIV and AIDS, and other health issues[35]. The biomarker questionnaire covered the identification of respondents, weights, heights, and hemoglobin measurements for children aged 0–5 years, weights, heights, HIV testing, and hemoglobin measurements for women aged 15–49 years[35]. The fieldworker questionnaire covered the background information on each field worker[35].

Anthropometric measurements. The weight of respondents was recorded in kilograms (kg) to the nearest decimal point and was measured using an electronic scale (SECA 878)[34,35]. Participants' heights were measured using a stadiometer in centimeters (cm) to one decimal point[34,35]. Body Mass Index (BMI) of respondents was calculated in kg/m^2 using weights (in kilograms) and heights (meters) of women of reproductive age (15–49 years) and classified according to WHO criteria as underweight (<18.5kg/m^2), normal weight (18.5–24.9kg/m^2), overweight (25.0–29.9kg/m^2), obesity (≥30.0kg/m^2 and ≤50.0kg/m^2), and overnutrition (≥25.0kg/m^2 and ≤50.0kg/m^2).

Wealth Index (WI). To calculate each household's wealth, we used the wealth index (WI) as a proxy indicator of household wealth[35]. This composite index used household key asset ownership variables to calculate each household wealth index from the SLDHS-2019 data[35]. These variables were the characteristics of the household's dwelling unit, for example, the source of water, type of toilet facilities, type of fuel used for cooking, number of rooms, ownership of livestock, possessions of durable goods, mosquito nets, and primary materials for the floor, roof, and walls of the dwelling place[35]. The respondent's household wealth index was calculated using computer analysis of household composite factors. It was then categorized into five quintiles: poorest, poorer, middle, richer, and richest wealth indices (Table 1).

Operational definitions.

Body Mass Index (BMI): Weight in kilograms divided by heights in meters squared (kg/m^2).

Underweight: BMI <18.5kg/m^2

Overweight: BMI ≥25.0kg/m^2 and ≤29.9kg/m^2

Obese: BMI ≥30.0kg/m^2 and ≤50.0kg/m^2

Overnutrition (Overweight and obese): BMI ≥25.0kg/m^2 and ≤50.0kg/m^2.

Enumeration Area (Clusters): An EA is a geographic area consisting of a convenient number of dwelling units that serve as a counting unit for the survey.

Data Collection: Data collection for this survey was conducted from May 14, 2019, to August 31, 2019[29]. The primary sampling unit (PSU), a cluster, was based on enumeration areas (EAs) obtained from the 2015 EA population census sampling frame[29].

The SLDHS-2019 used five validated questionnaires for the thematic parts of the survey[29]. The household questionnaire collected data on household environment, assets, and basic demographic information of household members. The woman's questionnaire collected data on women's reproductive health, domestic violence, and nutrition indicators[29]. The man's questionnaire collected data on men's health, while the biomarker questionnaire collected data on anthropometry and blood tests for mothers and children (0-5 years), and the fieldworker questionnaire collected data on background information of fieldworkers[36,37].

This secondary data analysis included women of reproductive age, 15-49 years, whose anthropometric characteristics were recorded with consent. Trained health technicians were deployed to measure the heights and weights of the participants to ensure the quality of anthropometric measurements[29].

Out of the weighted sample of 15,934 women in the dataset, 7,514 anthropometric measurements were included in the survey design, while 8,420 had invalid weight measurements due to erroneous and ineligible measurements. The weight measurement is vital for calculating the BMI of each participant, which was finally used for assessing the nutritional status of each respondent.

In some of the participants' results, heights were not well recorded, and we could only obtain completed anthropometric measurements for 7,514 women who were not lactating, non-pregnant, and post-menopausal women. In the final analysis, a weighted sample 7,514 was included in our secondary data analysis, as summarized in Table 1. A complete protocol with detailed explanations about data collection processes and sampling is available online[29].

Outcome variables: The first outcome variable for this study was stunting. It was coded as "1" for stunted women and "0" for not stunted. *Stunting* was defined as heights of <145cm ± Standard Deviations (SD) from the

median value set by the World Health Organization (WHO). The second outcome variable was underweight, which was defined as BMI<18.5kg/m^2 and coded as "1" for underweight women and "0" for normal weight. *Normal weight* was defined as a BMI of 18.5-24.9kg/m^2.

Independent variables: The independent variables in this study were based on previous studies, the WHO stunting framework, underweight, normal weight, and available information in the SLDHS-2019 database. We included nineteen independent variables in this data analysis.

Women's characteristics: Parity (categorized as para 0, para one-to-four, and five and above), work status (categorized as working-class versus not working), marital status (categorized as married versus not married/single), levels of education (categorized as no education, primary, secondary, and higher), age groups (categorized as 15-19, 20-29, 30-39, and 40-49 years), woman's stunting status (defined as heights <145cm for stunted and ≥145cm for not stunted women), and woman's BMI classification as normal BMI (18.5-24.9kg/m^2) and underweight (<18.5kg/m^2).

Household characteristics: These characteristics include regions of Sierra Leone (Northwest, Eastern, Western, Southern, and Northern); household wealth indices (categorized as richest, richer, middle, poorer, and poorest); sex of the head of household (female versus male); household size (less than six versus six and above); residency (urban versus rural); television viewing (yes versus no); reading magazines (yes versus no); listening to radios (yes versus no); smoking cigarettes (yes versus no); and alcohol use (yes versus no).

Ethical approval: This survey protocol was approved by the Sierra Leone Ethics and Scientific Review Committee (SLESRC) and the ICF Institutional Review Board. Written informed consent was obtained for each adult participant, and assent was obtained in the presence of a guardian or a legal representative for participants under eighteen years.

Statistical analysis: Frequency tables and proportions/percentages were used to describe summaries of categorical variables, while means and standard deviations (±SD) were used for continuous variables. Sample weights were used to account for unequal probability sampling in different study population strata and ensure the representativeness of the survey results at all levels[29]. Statistical software SPSS version 25.0 Statistical software complex samples package incorporating all variables in the analysis plan was used to account for the

multistage sampling design inherent in the DHS dataset, including individual sample weight, sample strata for sampling errors/design, and cluster numbers[38-40].

Using a complex sample package ensured the sampling design was incorporated into the analysis, leading to accurate and reliable results. Cross tabulations were conducted, and associations between socio-demographic characteristics and women's nutritional status (stunting and underweight), including their Odds ratios (OR) and P-values, were presented in Table 2 and Table 3.

To assess associations of each independent variable with dependent variables (stunting and underweight), a bivariate logistic regression analysis was conducted, and Crude Odds Ratios (COR), at 95% Confidence Intervals (CI) and P-values were presented. Independent variables were found significant at the bivariable level, and those with P-values ≤0.20 were included in the final multivariable logistic regression analysis model for each dependent variable. The final regression model excluded variables with P-values above 0.201 at the bivariate level.

Adjusted Odds Ratios (aOR), at 95% Confidence Intervals (CI), and corresponding P-values were calculated, with statistical significance levels set at 0.05.

Sensitivity analysis: Sensitivity analysis for stunting was conducted by excluding women with parity of five and above in the multivariable logistic regression model, as it had only 23(1.3%) stunted women. By excluding them from the final regression model, the other factors remained significant, and no substantial changes were observed in the strength of associations. The same statistical approach was used for studying underweight in this study population. Cross tabulations were conducted, and associations between socio-demographic characteristics and women's nutritional status (underweight versus normal weight), including their aOR at 95% CI and P-values, were presented (in Table 3), with no significant differences observed after excluding women with BMI ≥25.0kg/m^2.

Results.

The study was a demographic health survey conducted in Sierra Leone and included 7,514 women of reproductive age, 15-49 years (Table 1). Among the women, the majority belonged to the 20-29-year age group, accounting for 33.6%(2528/7514) of the total population. Women with parity ranging from one to four

represented just over 50%(51.8%; 3892/7514) of the total study population, while most participants resided in rural areas, 58.9%(4,422/7,514) of Sierra Leone. Male-headed households constituted slightly over two-thirds of the study population at 71.3%(5,356/7,514). Moreover, households with a size of six or more individuals constituted the majority at 60.1%(4,519/7,514).

Most of the study population were working-class women, representing 69.7%(5,234/7,514). Among marital status categories, married women constituted 63.8%(4,795/7,514). Regionally, women from the south of Sierra Leone constituted the most significant proportion at 24.4%(1,831/7,514), followed by the north at 24.2%(1,822/7,514), the east at 21.0%(1,579/7,514), the west at 16.7%(1,256/7,514), and the northwest at 13.7%(1,026/7,514).

In terms of educational level, the majority had no formal education, accounting for 47.5%(3,571/7,514), followed by secondary education at 35.2%(2,641/7,514), primary education at 13.5%(1,017/7,514), and a smaller proportion with higher education at 3.8%(285/7,514) (Table 1).

Regarding wealth indices, 21.7%(1,634/7,514) of women were in the richer wealth index category, followed by the poorest and middle at 20.4%(1,533/7,514) each, poorer wealth index at 19.0%(1,482/7,514), and the smallest proportion among the richest wealth index at 18.5%(1,388/7,514) (Table 1).

In terms of BMI categories, most women had normal BMI, accounting for 66.2%(4,974/7,514), followed by overweight at 19.7%(1,479/7,514), obese at 7.4%(5,59/7,514) and the smallest proportion among underweight women at 6.7%(502/7,514) (Table 1).

Regarding social activities, several participants did not watch television, accounting for 74.9%(5,625/7,514). Furthermore, most participants did not listen to radios 58.2%(4,372/7,514) and did not read magazines 93.5%(7,025/7,514). Additionally, most women of reproductive age did not smoke cigarettes 97.0%(7,290/7,514) and did not use alcohol 41.0%(3,081/7,514) (Table 1).

The prevalence of stunting: Out of 15,574 women in the SLDHS-2019, 48%(7,514/15,574) had valid height measurements. The mean height was 157.6cm with a standard deviation (SD) of ±6.3cm. The minimum recorded height was 107.7cm, and the maximum was 186.2cm. The overall prevalence of stunting in the study population was 1.5%(113/15,574) (Table 2).

Figure 1: The heights of women in the reproductive age (15-49 years) in Sierra Leone.

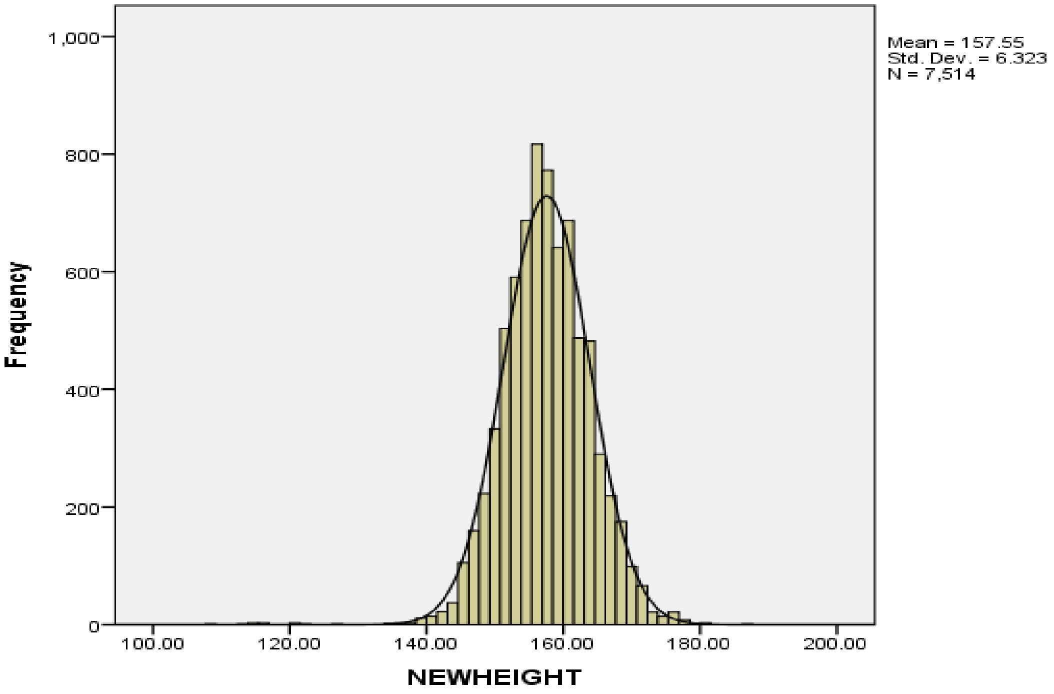

The source of data is SLDHS of 2019.
Figure 1 shows normally distributed heights among women aged (15-49) years in Sierra Leone. The mean height was 157.6 cm, SD±6.3.

The prevalence of underweight: Among the total population of women (n=7,514), the mean BMI was 23.8kg/m^2 (SD±4.7). The prevalence of underweight was 6.7%(502/7,514), with a minimum BMI recorded at 12.8kg/m^2. Within the underweight category, two outlier BMI values were 12.8kg/m^2 and 14.5kg/m^2, each representing 0.03% of the total study population. These outlier BMIs were situated on the left side of the normal distribution curve (Figure 1) (Table 2).

Figure 2: Frequency of underweight among age-groups of women (15-49 years) in the 2019 SLDHS.

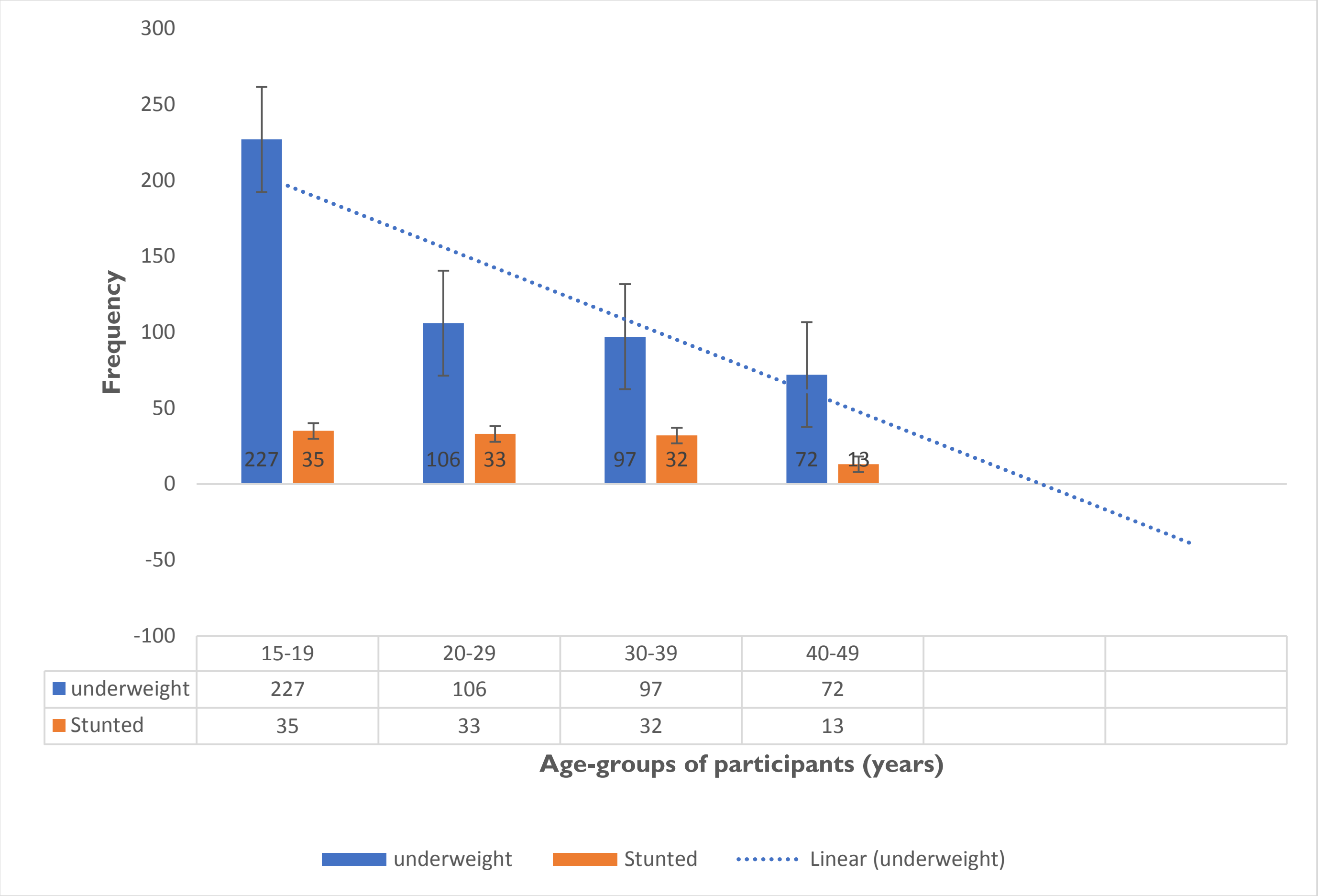

The source data is SLDHS of 2019.

Figure 2 shows the frequency of underweight as it decreased with age group populations, with the majority in the 15-19-year age group 45.2%(227/502), followed by the 20-19-year age group, 21.1%(106/502); 30-39-year age group 19.3%(97/502), and least among the 40-49-year age group 14.3%(72/502).

Correlates of stunting among women of reproductive age (15-49 years) in Sierra Leone: The study revealed that a primary level of education and belonging to the poorest wealth index were less likely factors of stunting among the study population. Women with a primary level of education had a 47% lower likelihood of being stunted (aOR=0.53,95%CI:0.30-0.94;p=0.029) than those with a secondary education. Similarly, women in the poorest wealth index had a 51% lower likelihood of stunting (aOR=0.49,95%CI:0.27-0.88;p=0.017) than in the middle wealth index. Other factors such as parity, residence (urban or rural), sex of the household head, household size, work status, marital status, regions of residence, listening to radios, reading of magazines, alcohol use, and smoking cigarettes did not significantly affect the occurrence of stunting among study participants (Table 2).

Correlates of underweight among women (15-49 years) in Sierra Leone. After adjusting for individual characteristics in the final multivariable logistic regression model, the determinants of underweight among Sierra Leonean women of reproductive age (15-49 years) were identified: Women with parity of one-to-four had a 1.48 times higher likelihood of being underweight (aOR=1.48, 95%CI:1.08-2.03; p=0.015) compared to those who never gave birth. In addition, women who listened to radios had a 1.41 times higher likelihood of being underweight (aOR=1.41,95%CI:1.14-1.74; p=0.002) than those who did not. However, being in the age group of 15-19 years was associated with a 54% lower likelihood of underweight (aOR=0.46, 95% CI:0.34-0.62; p<0.001) than the 20-29-year age-group and being in the age group of 40-49 years was associated with a 34% lower likelihood of underweight (aOR=0.66, 95%CI:0.45-0.97; p=0.035). Furthermore, having a primary level of education was associated with a 26% lower likelihood of underweight (aOR=0.74, 95% CI: 0.56-0.99; p=0.042) than a secondary level of education. None of the wealth indices showed a significant association with underweight in this study population (Table 3).

Table 1: Socio-economic and demographic characteristics of women in the reproductive age (15-49 years) in Sierra Leone.

Variables	Frequency (N=7,514)	Percent (%)
**Ages (years)**		
15-19	1,616	21.5
20-29	2,528	33.6
30-39	2,048	27.3
40-49	1,322	17.6
**Parity**		
Never gave birth	1,895	25.2
One to four	3,892	51.8
Five and above	1,727	23.0
**Type of residence**		
Urban	3,092	41.1
Rural	4,422	58.9
**Sex of the head of household**		
Male	5,356	71.3
Female	2,158	28.7
**Household size**		
Less than six	2,995	39.9
Six and above	4,519	60.1
**Work status**		
Not working	2,280	30.3
Working	5,234	69.7
**Marital status**		
Married	4,795	63.8
Not married	2,719	36.2
**Regions of Sierra Leone**		
East	1,579	21.0
North	1,822	24.2
Northwest	1,026	13.7
South	1,831	24.4
Western	1,256	16.7
**Levels of education**		
No formal education	3,571	47.5
Primary	1,017	13.5
Secondary	2,641	35.2
Higher	285	3.8
**Wealth Indices**		
Poorest	1,533	20.4
Poorer	1,428	19.0
Middle	1,531	20.4
Richer	1,634	21.7
Richest	1,388	18.5
**BMI categories (kg/m^2)**		
Underweight (<18.5)	502	6.7
Normal weight (18.5-24.9)	4,974	66.2
Overweight (25.0-29.9)	1,479	19.7
Obese ($\geq$30.0)	559	7.4
**Watching Television**		
Yes	1,889	25.1
No	5,625	74.9
**Listening to radios**		
Yes	3,142	41.8
No	4,372	58.2
**Reading of magazines**		
Yes	489	6.5
No	7.025	93.5
**Smoking of cigarettes**		
Yes	224	3.0
No	7,290	97
**Alcohol use**		
Yes	667	8.9
No	3,081	41.0

The data source is SLDHS-2019.

In Table 1 the majority of women of reproductive age (15-49 years) in Sierra Leone were in the 20-29-year age group 2528/7514(33.6%); parity of one-to-four 3892/7514(51.8%); of rural residence 4422/7514(58.9%); male-headed households 5356/7514(71.3%); household size of six and above 4519/7514(60.1%); working class 5234/7514(69.7%); married 4,795/7514(63.8%); from the South 1831/7514(24.4%); had no formal education 3571/7514(47.5%); richer wealth index 1634/7514(21.7%); normal weight 4974/7514(66.2%); did not watch television 5625/7514(74.9%); did not listen to radios 4372/7514(58.2%); did not read magazines 7025/7514(93.5%), did not smoke cigarettes 7290/7514(97.0%), and did not use alcohol 3081/7514(41.0%).

Table 2: Bi- and multivariable analysis of stunting among women (15-49 years) in SLDHS-2019.

Variables	Stunted (n=113) (n, %)	Not stunted (n=7,401) (n, %)	Unadjusted POR	95% CI	p value	aOR	95% CI	p value
**Age groups (years)**								
20-29	33(1.3)	2495(98.7)	**Reference**			**Reference**		
15-19	35(2.2)	1582(97.8)	0.597	0.370-0.965	0.035	0.815	0.437-1.520	0.419
30-39	32(1.6)	2016(98.4)	0.833	0.511-1.360	0.465	0.936	0.533-1.644	0.520
40-49	13(1.0)	1309(99.0)	1.332	0.699-2.539	0.384	1.559	0.741-3.277	0.818
**Parity**								
Never gave birth	38(2.0)	1857(98.0)	**Reference**			**Reference**		
One to four	52(1.3)	(3840(98.7)	1.511	0.991-2.304	0.055	1.489	0.792-2.801	0.216
Five and above	23(1.3)	1704(98.7)	1.516	0.900-2.555	0.118	1.524	0.659-3.524	0.324
**Residence**								
Rural	80(1.8)	4342(98.2)	**Reference**			**Reference**		
Urban	33(1.1)	3059(98.9)	1.708	1.136-2.569	0.010	1.257	0.614-2.572	0.531
**Sex of the household head**								
Male	76(1.4)	5280(98.6)	**Reference**					
Female	37(1.7)	2121(98.3)	0.825	0.555-1.226	0.342			
**Household size**								
Six and above	59(1.3)	4460(98.7)	**Reference**			**Reference**		
Less than six	54(1.8)	2941(98.2)	0.72	0.497-1.045	0.084	0.761	0.518-1.112	0.166
**Work status**								
Not working	33(1.4)	2247(98.6)	**Reference**					
Works	80(1.5)	5154(98.5)	0.946	0.946-1.424	0.791			
**Marital status**								
Not married	48(1.8)	2671(98.2)	**Reference**			**Reference**		
Married	65(1.4)	4730(98.6)	1.308	0.898-1.905	0.162	1.303	0.763-2.224	0.333
**Region of residence**								
East	24(1.5)	1555(98.5)	**Reference**			**Reference**		
North	25(1.4)	1797(98.6)	1.109	0.631-1.950	0.718	1.167	0.656-2.074	0.600
Northwest	8(0.8)	1018(99.2)	1.964	0.879-4.389	0.100	1.908	0.846-4.302	0.119
South	43(2.3)	1788(97.7)	0.642	0.388-1.062	0.085	0.695	0.415-1.162	0.165
Western	13(1.0)	1243(99.0)	1.476	0.748-2.910	0.261	0.821	0.368-1.831	0.630
**Level of education**								
Secondary	32(1.2)	2609(98.8)	**Reference**			**Reference**		
No formal education	56(1.6)	3515(98.4)	0.770	0.497-1.192	0.241	0.624	0.351-1.107	0.107
Primary	25(2.5)	992(97.5)	0.487	0.287-0.825	0.008	0.531	0.300-0.938	0.029
Higher	0	285(100)	19814180	0	0.994	12203543	0	0.994
**Wealth Indices**								
Middle	17(1.1)	1514(98.9)	**Reference**			**Reference**		
Poorest	41(2.7)	1492(97.3)	0.409	0.231-0.722	0.002	0.485	0.268-0.880	0.017
Poorer	21(1.5)	1407(98.5)	0.752	0.395-1.432	0.386	0.778	0.404-1.497	0.452
Richer	24(1.5)	1610(98.5)	0.753	0.403-1.408	0.374	0.654	0.302-1.417	0.282
Richest	10(0.7)	1378(99.3)	1.547	0.706-3.391	0.275	1.068	0.377-3.026	0.902
**Watching television**								
No	96(1.7)	5529(98.3)	**Reference**			**Reference**		
Yes	17(0.9)	1872(99.1)	1.912	1.139-3.211	0.014	1.385	0.744-2.578	0.304
**Listens to radio**								
No	74(1.7)	4298(98.3)	**Reference**			**Reference**		
Yes	39(1.2)	3103(98.8)	1.37	0.927-2.204	0.114	0.902	0.585-1.392	0.642
**Reading of magazines**								
No	108(1.5)	6917(98.5)	**Reference**					
Yes	5(1.0)	484(99.0)	1.511	0.614-3.722	0.369			
**Smokes cigarettes**								
No	109(1.5)	7181(98.5)	**Reference**					
Yes	4(1.8)	220(98.2)	0.835	0.305-2.285	0.725			
**Alcohol use**								
No	45(1.5)	3036(98.5)	**Reference**					
Yes	8(1.2)	659(98.8)	1.221	0.573-2.602	0.605			

aOR: adjusted Odds Ratio; CI: Confidence Interval; COR: Crude Odds Ratio; SLDHS: Sierra Leone Demographic and Health Survey.

In Table 2, the determinants of stunting among Sierra Leone women of reproductive age were less likely among women of primary level of education, aOR=0.53,95%CI:0.30-0.94;p=0.029 and those in the poorest wealth index aOR=0.49,95%CI:0.27-0.88; p=0.017.

Table 3: Prevalence and determinants of underweight among women (15-49 years) in SLDHS-2019.

Variables	Under-weight (N=502) (n, %)	Normal weight (N=4,974) (n, %)	Unadjusted (COR)	95% CI	p value	Adjusted (aOR)	95% CI	p value
**Age groups (years)**								
20-29	106(5.6)	1,773(94.4)	**Reference**			**Reference**		
15-19	227(16.0)	1,192(84.0)	0.314	0.246-0.400	<.001	0.457	0.335-0.624	<.001
30-39	97(7.2)	1,244(92.8)	0.767	0.577-1.019	0.068	0.746	0.536-1.037	0.081
40-49	72(8.6)	765(91.4)	0.635	0.465-0.867	0.004	0.663	0.453-0.972	0.035
**Parity**								
Never gave birth	225(14.5)	1,330(85.5)	**Reference**			**Reference**		
One to four	182(6.7)	2537(93.3)	2.358	1.918-2.899	<.001	1.479	1.079-2.029	0.015
Five and above	95(7.9)	1,107(92.1)	1.971	1.531-2.538	<.001	1.362	0.876-2.117	0.170
**Residence**								
Rural	340(9.7)	3,156(90.3)	**Reference**					
Urban	162(8.2)	1,818(91.8)	1.209	0.994-1.470	0.057			
**Sex of household head**								
Male	343(8.7)	3,621(91.3)	**Reference**			**Reference**		
Female	159(10.5)	1,353(89.5)	0.806	0.661-0.983	0.033	0.866	0.701-1.071	0.186
**Household size**								
Six and above	321(9.7)	2,998(90.3)	**Reference**					
Less than six	181(8.4)	1,976(91.6)	1.169	0.966-1.415	0.109			
**Work status**								
Not working	191(11.1)	1,529(88.9)	**Reference**			**Reference**		
Working	311(8.3)	3,445(91.7)	1.384	1.144-1.673	0.001	1.011	0.800-1.277	0.928
**Marital status**								
Not Married	270(12.6)	1,872(87.4)	**Reference**			**Reference**		
Married	232(7.0)	3,102(93.0)	1.928	1.603-2.319	<.001	1.251	0.936-1.672	0.130
**Region of residence**								
East	96(8.1)	1,082(91.9)	**Reference**			**Reference**		
North	153(10.5)	1,305(89.5)	0.757	0.579-0.989	0.041	0.765	0.581-1.008	0.057
Northwest	73(9.2)	724(90.8)	0.88	0.640-1.210	0.431	0.898	0.648-1.243	0.515
South	134(10.3)	1,173(89.7)	0.777	0.590-1.022	0.071	0.789	0.595-1.045	0.098
Western	46(6.2)	690(93.8)	1.331	0.925-1.916	0.777	1.248	0.823-1.892	0.298
**Level of education**								
Secondary	185(9.5)	1,755(90.5)	**Reference**			**Reference**		
No formal education	211(8.1)	2,399(91.9)	1.199	0.975-1.474	0.086	0.886	0.662-1.186	0.417
Primary	96(12.3)	686(87.7)	0.753	0.580-0.979	0.034	0.742	0.557-0.989	0.042
Higher	10(6.9)	134(93.1)	1.413	0.730-2.733	0.305	0.677	0.338-1.357	0.272
**Wealth Indices (WI)**								
Middle	121(10.3)	1,050(89.7)	**Reference**			**Reference**		
Poorest	104(8.3)	1,156(91.7)	1.281	0.973-1.666	0.078	1.236	0.929-1.646	0.146
Poorer	120(10.2)	1,053(89.8)	1.011	0.775-1.320	0.935	0.935	0.711-1.229	0.630
Richer	97(9.1)	974(90.9)	1.157	0.874-1.533	0.309	1.150	0.850-1.557	0.365
Richest	60(7.5)	741(92.5)	1.423	1.030-1.967	0.032	1.158	0.782-1.713	0.464
**Watching television**								
No	404(9.5)	3,851(90.5)	**Reference**					
Yes	98(8.0)	1,123(92.0)	1.202	0.955-1.514	0.117			
**Listens to radio**								
No	350(10.4)	3,007(89.6)	**Reference**			**Reference**		
Yes	152(7.2)	1,967(92.8)	1.506	1.235-1.837	<.001	1.407	1.136-1.742	0.002
**Reading magazines**								
No	473(9.1)	4,698(90.9)	**Reference**					
Yes	29(9.5)	276(90.5)	0.958	0.646-1.421	0.832			
**Smokes cigarettes**								
No	484(9.1)	4,835(90.9)	**Reference**					
Yes	18(11.5)	139(88.5)	0.773	0.469-1.274	0.313			
**Alcohol use**								

No	140(6.7)	2,005(93.3)	**Reference**		
Yes	35(7.5)	429(92.5)	0.856	0.582-1.258	0.428

aOR: adjusted Odds Ratio; CI: Confidence Interval; COR: Crude Odds Ratio; SLDHS: Sierra Leone Demographic and Health Survey.

In Table 3, the determinants of underweight among Sierra Leone women was likely among women with parity of one-to- four aOR=1.48,95%CI:1.08-2.03;p=0.015 and those who listened to radios, aOR=1.41,95%CI:1.14-1.74;p=0.002. However, underweight was less likely among age-group of 15-19 years, aOR=0.46,95%CI:0.34-0.62;p<0.001; age-group of 40-49 years, aOR=0.66,95%CI:0.45-0.97;p=0.035, and those with primary level of education, aOR=0.74,95%CI:0.56-0.99;p=0.042.

Discussion: This population-based study provides valuable insights into the prevalence and correlates of underweight and stunting among women of reproductive age (15-49 years) in Sierra Leone (Table 1, Figure 1, and Figure 2). The prevalence of stunting in Sierra Leone, at 1.5%, is higher than that reported in the DHS of Kenya (less than 1%)[41], and Uganda (1.3%)[36,42] but lower than Tanzania (less than 3%)[43].

Stunting among women of reproductive age is a significant concern, as it reflects long-term exposure to inadequate nutrition, infection, and environmental stress. The consequences of stunting are far-reaching, particularly for girls and women of reproductive age[45], and the effects are experienced at individual, community, and national levels[46]. It is alarming to note that an estimated 450 million adult women in developing countries are stunted due to malnutrition during childhood[47]. Therefore, addressing stunting among women is crucial for improving maternal and child health outcomes.

Stunting among women of reproductive age (15-49 years) in Sierra Leone from the 2019 DHS. Women with a primary level of education were 47% less likely to be stunted than those with a secondary level. Similarly, women in the poorest wealth index were 51% less likely to be stunted compared to those in the middle wealth index. These findings highlight the importance of education and socio-economic status in mitigating the risk of stunting among women. However, no other factors were significantly associated with stunting in this study population (Table 2).

In contrast, the correlates of being underweight differed from those of stunting (Table 2 and Table 3). A parity of one to four and listening to radios were identified as significant factors associated with being underweight (Table 2). Women with a parity of one to four were 1.48 times more likely to be underweight than those who had never given birth. (Table 3). On the other hand, age groups of 15-19 years and 40-49 years, as well as

primary education, were less likely to be underweight. These findings suggest that different factors contribute to underweight compared to stunting among women (15-49 years) in Sierra Leone (Table 3)[48].

The underlying reasons for the low likelihood of primary level of education on stunting and underweight in the study population remain unclear, highlighting the need for in-depth exploration through qualitative research. Conducting qualitative studies would allow for a deeper understanding of the factors and mechanisms contributing to the observed association between primary level of education and better nutritional outcomes. By delving into women's lived experiences and socio-cultural context, qualitative research can provide valuable insights to unravel the complex dynamics at play. Further investigation through qualitative research is warranted to understand why the primary level of education emerges as a protective factor against stunting and underweight in this population.

It is interesting to note that women in the poorest wealth index were less likely to be stunted compared to women in the middle wealth index (Table 2). This finding contradicts many studies in other African countries where stunting is more prevalent among women in the poorest wealth indices[36,42,49].

Studies on stunting among children in Sierra Leone from the same SLDHS-2019 show a high prevalence among children below five years[50]. However, our findings that women in the reproductive age group (15-49 years) from the same data source (SLDHS-2019) had no likely association with any age group were unique. In comparison, children below five years experienced a high prevalence of stunting (31.6% in rural versus 24.0% in urban areas)[50].

The unique finding in Sierra Leone necessitates further investigation to explore the underlying factors contributing to this difference. It is plausible that low-income households have adopted favorable eating habits and practices, such as consuming locally available foods like *plasas*. *Plasas*, a mixture of green leaves with palm oil and fish, is affordable and highly nutritious. Understanding the dietary choices and affordability of nutritious foods among low-income households could provide valuable insights into the observed findings.

Stunting is a chronic condition that begins during the prenatal period and persists through early childhood and adolescence, with the first two years of life being particularly critical[43,49]. Previous studies have highlighted the high prevalence of stunting among women of reproductive age in low-to-middle-income countries, as stunted children often continue to experience stunting into adulthood[50,51]. However, it is essential to note that some

individuals stunted in childhood overcame these challenges by accessing education, obtaining better employment opportunities, increasing their income, or marrying into higher socio-economic strata. As a result, they may have transitioned from lower to higher wealth indices, indicating the potential for social mobility and improvement in their overall well-being. This socio-economic progress achieved by these women may have played a role in the observed outcome of low socio-economic status being unlikely for undernutrition.

In addition, many studies show that improved drinking water was associated with a lower risk of stunting and that improved water was a proxy for less exposure to enteric pathogens[52]. Watanabe and Petri discussed that environmental enteropathy is a chronic disease caused by continuous exposure to faecally contaminated food and water that does not produce symptoms but contributes to poor physical development[52]. This finding may have been a factor experienced among populations in other countries but not in Sierra Leone.

These findings on stunting among women in Sierra Leone contrast with another in Uganda, where the population in the Southwestern northeastern (pygmies and Batwa) was naturally shorter compared to the average Ugandan population[53,54,55]. More to this could be explained by genetic factors, which play a part at the individual level, where it is likely that women of reproductive age in Sierra Leone were generally taller because of their genetic makeup[12]. A contrasting scenario was observed in western Uganda among the pygmies and others who were generally shorter than the average Ugandan population[36,53,54]. However, the situation can be determined further by conducting more comprehensive studies on the height profiles of women in Sierra Leone over several decades to determine the changing patterns of women's heights stratified by regions of the country.

Also, one of the insignificant factors of stunting was the age group of 15-19 years, which is linked to an age group with rapid growth, increased activities, and a high need for adequate nutrients (Table 2). The need for adequate nutrients and diet are paramount for the growth and development of persons in that age category. Our findings that there were no associated factors of stunting with women in specific age groups and poor household wealth indices were inconsistent with literature from Bangladesh and other countries[55-60].

Genetic predilections and environmental factors mainly determine adult heights. In addition to genetic impacts, incomes, social status, infections, and nutrition have been shown to affect body height in the European population[60]. Environmental factors are likely to be more important determinants of height in low and middle-

income countries because environmental stress, including food availability and infections, is higher in those countries compared to high-income countries.

Perkins *et al.* explained in their review that short adult stature in low-and-middle-income countries is mainly because of the cumulative net impact of nutrition associated with disease and environmental conditions, such as socio-economic status[56].

The correlates of stunting and underweight among women of reproductive age (15-49 years) in Sierra Leone were different and raised our concerns (Table 1, Table 2). Many factors singly or collectively contribute to underweight and stunting, including eating patterns, food types, their availability, infections, diseases, physical activity levels, and sleep routines5,6. In addition to social determinants of health, genetics and taking certain medications have been shown to play essential roles in undernutrition in a population[5,6,10,61].

If compared with overweight and obesity the two are mainly caused by food consumption and activities where people gain weight when they eat more calories than they burn through daily activities[61,62]. Also, environmental factors around us matter in the development of obesity and overweight, just like stunting and underweight[62]. The world influences our ability to maintain a healthy weight and lifestyle. That has been seen in many African communities where people who are obese are considered healthy, living a prosperous and fulfilling life, an issue which is admired by women in many African communities[62].

On the other side of the spectrum, some communities have begun to admire smaller sizes and equate them to successful and healthy lives. In this, several blue-colored individuals have begun to reduce their sizes by conducting regular exercises, eating organic foods, fasting, eating less fast foods, less snacking, taking fewer salts and sugars, living a less sedentary lifestyle, riding bicycles, or walking to work, sleeping better, avoiding stressful and mental health situations[62].

Perhaps most interesting from this study is that correlates of underweight and stunting among women in Sierra Leone were different, a factor that should be determined through a comprehensive study, unearthing the underlying reasons. This finding contrasts with many studies in the African continent[36,41,42,43,45].

Chronic effects of malnutrition in early childhood due to inadequate nutrients and unavailability of food are reflected in later life by stunting and other lifelong consequences such as reduced cognitive function and maternal and child health complications, which we did not find in this study population (Table 2).

These correlates of stunting must be addressed in Sierra Leonne's women if improvement in maternal and child health indicators is to be achieved soon in this country[63]. Feeding habits, diets, and food availability for young women in Sierra Leone are prioritized as soon as possible since many young women of reproductive age are affected by stunting and underweight (Table 1 and Table 2).

In addition, early childhood nutrition programs (for example, school feeding programs) could be a welcome intervention for school-going female children.

It is worth noting that there is limited literature on stunting among women of reproductive age in Sierra Leone, with most studies focusing on underweight. Therefore, the findings of this study contribute to filling this knowledge gap and could be used for setting a proper agenda for the population.

Strengths and limitations of this study: This study has several strengths. First, the data quality of this study was assured as the SLDHS-2019 used well-trained field personnel, standardized protocols, and validated tools in data collection processes. Second, this study utilized a nationally representative sample population of women in the reproductive age of 15-49 years. As a result, the study's findings can be generalizable to the target population in Sierra Leone and many low-to-middle-income countries in the African continent. Third, using validated tools and calibrated instruments by the SLDHS-2019, the generated estimates are more robust than other studies in Sierra Leone's context. In addition, we used data with a large sample size, which was collected, entered, and cleaned by a team of well-trained and highly experienced scientists, thus limiting mistakes in the dataset used in the analysis. Finally, as we used the concentration index, these findings are more robust in predicting socio-economic inequalities among the study population.

However, this study had limitations that warrant further discussion. First, the SLDHS-2019 was a cross-sectional survey. As a result, we cannot establish a sequential relationship between explanatory and outcome variables. Second, due to the absence of some crucial data, several significant variables, such as food security and dietary diversity, could not be included in the final model for the analysis. Third, the SLDHS-2019 did not collect individual incomes and expenditures but household data. It used a wealth index as a proxy indicator for household wealth. Fourth, SLDHS collected data only on 15–49-year-old women of reproductive age in Sierra Leone. With the current changes in adolescents' reproductive actions and behaviors, there are children less than 15 years old who have gone through an entire cycle of reproduction. As a result, the distribution of

undernutrition among women below and beyond this age group (15-49 years) was not factored in the analysis. Finally, most data on predictors of undernutrition were based on self-reported information. They were not verified through records, which risks socially acceptable answers, hence social desirability bias in this result.

Generalizability of results: Results from this study can be generalized to women of reproductive age (15-49 years) in resource settings in low-to-middle-income countries.

Conclusion. The prevalence of underweight and stunting among women of reproductive age (15-49 years) in Sierra Leone was lower compared to regional and world data. This study highlights similarities and differences in this population's prevalence and correlates of undernutrition. Underweight and stunting were less likely in women with primary education, while parity of one-to-four and listening to radios were significantly associated with underweight. Further trend studies using DHS data from 2010, 2014, and 2019 are warranted to understand the dynamics of undernutrition in Sierra Leone.

Furthermore, there is a need to improve the social determinants of health in Sierra Leone in women of reproductive age, including school feeding programs among children and adolescents.

In addition, it is essential to note that this study's findings have important implications for addressing maternal and child health in Sierra Leone. The identified correlates of stunting and underweight should be addressed through targeted interventions. Improving feeding habits, ensuring dietary diversity, and addressing food availability for young women in Sierra Leone should be prioritized. Early childhood nutrition programs, such as school feeding programs, could be effective interventions for improving the nutritional status of school-going female children.

In summary, this study provides valuable insights into the prevalence and correlates of underweight and stunting among women of reproductive age in Sierra Leone. The findings highlight the importance of education, socio-economic status, and environmental factors in influencing nutritional outcomes. Addressing the correlates of stunting and underweight among women is essential for improving maternal and child health indicators in Sierra Leone. Further research is needed to explore the underlying reasons for the observed differences in correlates between stunting and underweight and to develop targeted interventions to alleviate these nutritional challenges.

Abbreviations

aOR=adjusted Odds Ratio; BMI=Body Mass Index; CI=Confidence Intervals; COR=Crude Odds Ratio; DHS=Demographic Health Survey; EA=Enumeration Areas; SD=Standard Deviation; SLDHS=Sierra Leone Demographic Health Survey; SDGs=Sustainable Development Goals; USAID=United States of America Agency for International Development; WHO=World Health Organization.

Declarations

Ethics approval and consent to participate: This study on women of reproductive age (15-49 years) followed relevant institutional guidelines and regulations and was approved by the Sierra Leone Ethics and Scientific Review Committee (SLESRC) and ICF Institutional Review Board. The study was conducted according to institutional guidelines, where written informed consent was obtained from each adult participant. For those under 18 years, assent was obtained in the presence of parents or legal representatives.

Consent for publication: Not applicable

Availability of data and material: All datasets supporting this article's conclusion are within this article and are accessible by a reasonable request to the corresponding author.

Competing interests: All authors declare no conflict of interest.

Funding: Most funds for this study were contributions by individual research members.

Authors' contributions: DLK, JA, and FWDO designed the study. JA, ENI, FWDO, and DLK supervised data management. ENI, JA, MAT, LN, EO, NOA, OE, and DLK analyzed and interpreted data. NOA, ENI, FWDO, JA, MAT, EO, LN, KK, AN, OE, JBM, RK, and DLK wrote and revised the manuscript. All the authors approved the revised manuscript.

Acknowledgment: We acknowledge with many thanks for the assistance from 2019 SLDHS for the information obtained.

Authors' Information: Dr. Nelson Onira Alema is a Lecturer in the Department of Anatomy, Faculty of Medicine, Gulu University, Gulu City, Uganda; Dr. Eric Nzirakaindi Ikoona (ENI) is a Technical Director at ICAP at the University of Columbia, Sierra Leone; Dr. Mame Awa Toure (MAT) is at ICAP at the University of Columbia, Freetown, Sierra Leone; Dr. Oliver Eleeza (OE) is an associate director strategic information, ICAP at Columbia University, Sierra Leone; Dr. John Bosco Matovu (JBM) is at ICAP at the University of Columbia, Nairobi, Kenya; Dr. Amon Njenga (AN) is at ICAP at the University of Columbia, Freetown, Sierra Leone; Dr. Lucy Namulemo (LN) is at Foothills Community Based interventions, Monticello, Kentucky, USA; Dr. Ronald Kaluya (RK) is at Uganda Counseling and Support Services, Kampala, Uganda; Dr. Kassim Kamara (KK) is at the Directorate of health security and emergencies, Ministry of Health and Sanitation, Sierra Leone; Dr. Freddy Wathum Drinkwater Oyat (FWDO) is a senior physician, a public health specialist, and a member of Uganda Medical Association, UMA-Acholi branch, Gulu City, Uganda; Dr. Judith Aloyo (JA) is the Deputy Chief of Party at the Rhites-N, Acholi, Gulu City, Uganda; Dr. Emmanuel Olal (EO) is a Physician and a public health specialist at Yotkom Medical Centre, Kitgum, Uganda; Prof. David Lagoro Kitara (DLK) is a Takemi fellow of Harvard University and a Professor at Gulu University, Faculty of Medicine, Department of Surgery, Gulu City, Uganda.

References

1. Leslie Elder, Elizabeth Ransom. Nutrition of women and adolescent girls: Why it matters. https://www.prb.org/resources/nutrition-of-women-and-adolescent-girls-why-it-matters/.
2. Sun Movement. Scaling up nutrition: a framework for action. Sun Movement [homepage on the Internet]. 2011. http://scalingupnutrition.org/wp-content/uploads/ pdf/SUN_Framework.pdf.

3. Rotimi C, Okosun I, Johnson L. The distribution of chronic energy deficiency among adult Nigerian men and women. Eur J Clin Nutr.1999;53(9):734-739.
4. World Health Organization. Physical status: the use and interpretation of anthropometry. Geneva: WHO, 1995.
5. Merchant KM, Kurtz KM. Women's nutrition through the life cycle: social and biological vulnerabilities. In: Marge Koblinsky, Judith Timyan and Jill Gay, editors. The health of women: a global perspective. Boulder: Westview Press, 1993;63-90.
6. Bitew FH, Telake DS. Undernutrition among women in Ethiopia: rural-urban disparity. Calverton: ICF Macro, 2010.
7. Stewart CP, Iannotti L, Dewey KG, Michaelsen KF, Onyango AW. Contextualizing complementary feeding in a broader framework for stunting prevention. Matern Child Nutr. 2013;9(suppl 2):27-45.
8. Prado EL, Dewey KG. Nutrition and brain development in early life. Nutr Rev. 2014;72:267-284.
9. Grantham-McGregor S, Cheung YB, Cueto S, Glewwe P, Richter L, Strupp B. Developmental potential in the first 5 years for children in developing countries. Lancet. 2007;369:60-70.
10. Ashraf Soliman, Vincenzo De Sanctis, Nada Alaaraj, Shayma Ahmed, Fawziya Alyafei, Noor Hamed, *et al.,* Early and Long-term Consequences of Nutritional Stunting: From Childhood to Adulthood. Acta Biomed. 2021;92(1): e2021168.
11. Shafique S, Akhter N, Stallkamp G. Trends of under- and overweight among rural and urban poor women indicate the double burden of malnutrition in Bangladesh. Int J Epidemiol. 2007; 36:449–457.
12. Mallia T, Grech A, Hili A. Genetic determinants of low birth weight. Minerva Ginecol. 2017; 69:631–643.
13. Dahlui M, Azahar N, Oche OM. Risk factors for low birth weight in Nigeria: evidence from the 2013 Nigeria Demographic and Health Survey. Glob Health Action 9. 2016;28822.
14. Nnam NM. Improving maternal nutrition for better pregnancy outcomes. Proc Nutr Soc. 2017;74:454-459.
15. Khan MN, Rahman MM, Shariff AA. Maternal undernutrition and excessive body weight and risk of birth and health outcomes. Arch Public Health.2017;75:12.
16. Xu H, Shatenstein B, Luo Z-C. Role of nutrition in the risk of preeclampsia. Nutr Clin Care. 2009; 67:639-657.
17. Chopra M, Galbraith S & Darnton-Hill. A global response to a global problem: the epidemic of overnutrition. Bull World Health Organ. 2002; 80:952–958.
18. Ng M, Fleming T, Robinson M. Global, regional, and national prevalence of overweight and obesity in children and adults 1980–2013: a systematic analysis. Lancet. 2014; 384:766–781.
19. Zahangir MS, Hasan MM, Richardson A. Malnutrition, and non-communicable diseases among Bangladeshi women: an urban–rural comparison. Nutr Diabetes. 2017; 7, e250.
20. Ly KA, Ton TGN, Ngo QV. Double burden: a cross-sectional survey assessing factors associated with underweight and overweight status in Danang, Vietnam. BMC Public Health.2013;13:35.
21. Fenn B, Bulti AT, Nduna T. An evaluation of an operations research project to reduce childhood stunting in a food-insecure area in Ethiopia. Public Health Nutr.2012;15:1746–1754.
22. World Health Organization, UNICEF & US Agency for International Development (2015) Improving Nutrition Outcomes with Better Water, Sanitation and Hygiene: Practical Solutions for Policy and Programmes. Geneva: WHO. 2015.
23. Harris-Fry H, Azad K, Kuddus A. Socioeconomic determinants of household food security and women's dietary diversity in rural Bangladesh: a cross-sectional study. J Health Popul Nutr. 2015;33:12.
24. Islam A, Islam N, Bharati P. Socio-economic and demographic factors influencing nutritional status among early childbearing young mothers in Bangladesh. BMC Women's Health. 2016;16:58.
25. Hossain MG, Bharati P, Aik SAW. Body mass index of married Bangladeshi women: trends and association with socio-demographic factors. J Biosoc Sci. 2012;44:385-399.
26. Bhuiya A & Mostafa G. Levels and differentials in weight, height, and body mass index among mothers in a rural area of Bangladesh. J Biosoc Sci. 1993;25:31-38.

27. Baqui AH, Arifeen SE, Amin S. Levels and correlates of maternal nutrition status in urban Bangladesh. Eur J Clin Nutr. 1994;48:349–357.
28. Ahsan KZ, Arifeen SE, Al-Mamun MA. Effects of individual, household, and community characteristics on child nutritional status in the slums of urban Bangladesh. Arch Public Health.2017;75:9.
29. Statistics Sierra Leone-StatsSL, ICF. Sierra Leone: Demographic and health survey 2019. 2020. https://www.dhsprogram.com/pubs/pdf/FR365/FR365. pdf.
30. World Food Program (WFP). State of Food Security in Sierra Leone 2015 Comprehensive Food Security and Vulnerability Analysis Data collected September - October 2015. 2015 Sierra Leone CFSV. https://efaidnbmnnnibpcajpcglclefindmkaj/https://documents.wfp.org/stellent/groups/public/documents/ena/wfp288316.pdf?iframe.
31. Worldometer. The population of Sierra Leone. 2022. https://www.worldometers.info/world-population/sierra-leone-population/
32. Sierra Leone NHSSP. National Health Sector Strategic Plan 2017 – 2021. chrome-extension://efaidnbmnnnibpcajpcglclefindmkaj/https://extranet.who.int/countryplanningcycles/sites/default/files/planning_cycle_repository/sierra_leone/sierra_leone_nhssp_2017-21_final_sept2017.pdf
33. World Bank (WB). Microdata in Sierra Leone, 2019. Demographic and Health Survey 2019. Microdata Library. 2019. https://microdata.worldbank.org/index.php/catalog/3826.
34. Statistics Sierra Leone (Stats SL) and ICF. 2020. Sierra Leone Demographic and Health Survey 2019. Freetown, Sierra Leone, Rockville, Maryland, USA: Stats SL and ICF. 2020. chrome-extension://efaidnbmnnnibpcajpcglclefindmkaj/https://dhsprogram.com/pubs/pdf/FR365/FR365.pdf
35. Pamela Okot Atim, Smart Geoffrey Okot, Eric Nzirakaindi Ikoona, Lucy Namulemo, Judith Aloyo, David Lagoro Kitara, *et al.* Factors and prevalence of undernutrition among women of reproductive age (15-49 years) in Sierra Leone. A secondary data analysis of the demographic health survey of 2019. Research Square. 2023. DOI: https://doi.org/10.21203/rs.3.rs-3101722/v1.
36. Sserwanja Q, Mukunya D, Habumugisha T, Mutisya LM, Tuke R, Olal E. Factors associated with undernutrition among 20-to 49-year-old women in Uganda: a secondary analysis of the Uganda demographic health survey 2016. BMC Public Health. 2020;20:1644.
37. Zambia Statistics Agency (ZSA), Ministry of Health (MOH). University Teaching Hospital Virology Laboratory – (UTH-VL), ICF. Zambia demographic and health survey. 2018. https://www.dhsprogram.com/pubs/pdf/ FR361/FR361.pdf.
38. Agbadi P, Eunice TT, Akosua AF, Owusu S. Complex samples logistic regression analysis of predictors of the current use of modern contraceptive among married or in-union women in Sierra Leone: insight from the 2013 demographic and health survey. PLoS One. 2020;15:e0231630.
39. Zou D, Lloyd JEV, Baumbusch JL. Using SPSS to analyze complex survey data: a primer. J Mod Appl Stat Methods. 2020;18:2-22.
40. Croft Trevor N, Aileen MJM, Courtney KA. Guide to DHS Statistics. ICF; 2018.
41. Kenya National Bureau of Statistics, Ministry of Health/Kenya, National AIDS Control Council/Kenya, Kenya Medical Research Institute, Population NCf, Development/Kenya: Kenya Demographic and Health Survey 2014. Rockville; 2015.
42. Quraish Sserwanja. Socio-economic determinants of undernutrition among women of reproductive age in Uganda: a secondary analysis of the 2016 Uganda demographic health survey. UPPSALA UNIVERSITET. 2019;1-49. *http://www.diva-portal.org/smash/get/diva2:1367320/FULLTEXT01.pdf*.
43. Ministry of Health CD, Gender, Elderly, Children - MoHCDGEC/Tanzania Mainland, Ministry of Health - MoH/Zanzibar, National Bureau of Statistics - NBS/Tanzania, Office of Chief Government Statistician - OCGS/Zanzibar, ICF: Tanzania Demographic and Health Survey and Malaria Indicator Survey 2015–2016. Dar es Salaam: MoHCDGEC, MoH, NBS, OCGS, and ICF; 2016.
44. Christian P, Smith ER. Adolescent Undernutrition: Global Burden, Physiology, and Nutritional Risks. Annals of Nutrition and Metabolism. 2018;72(4):316-28.

45. Melaku YA, Zello GA, Gill TK, Adams RJ, Shi Z. Prevalence, and factors associated with stunting and thinness among adolescent students in Northern Ethiopia: a comparison to World Health Organization standards. Archives of Public Health. 2015;73(1):44.
46. Reinhardt K, Fanzo J. Addressing Chronic Malnutrition through Multi-Sectoral, Sustainable Approaches: A Review of the Causes and Consequences. Frontiers in nutrition. 2014;1:13.
47. Haseen F. Malnutrition among ultra poor women in Bangladesh: Malnutrition among Bangladeshi Women in Ultra Poor Households: Prevalence and Determinants. Germany: LAP Lambert Academic Publishing, 2010;52-3.
48. Eric Nzirakaindi Ikoona, Lucy Namulemo, Ronald Kaluya, Freddy Wathum Drinkwater Oyat, Judith Aloyo, David Lagoro Kitara. Prevalence and factors associated with underweight among 15–49-year-old women in Sierra Leone: A secondary Data Analysis of Sierra Leone Demographic Health Survey of 2019. BMC Women's Health. 2023;23:192. *https://doi.org/10.1186/s12905-023-02358-4*.
49. Negash WD, Fetene SM, Shewarega ES. Multilevel analysis of undernutrition and associated factors among adolescent girls and young women in Ethiopia. BMC Nutr. 2022;8:104. https://doi.org/10.1186/s40795-022-00603-x.
50. Sserwanja Q, Kamara K, Mutisya LM, Musaba MW, Ziaei S. Rural and Urban Correlates of Stunting Among Under-Five Children in Sierra Leone: A 2019 Nationwide Cross-Sectional Survey. Nutr Metab Insights. 2021;14:11786388211047056. doi:10.1177/11786388211047056.
51. Cesar G Victora, Parul Christian, Luis Paulo Vidaletti, Giovanna Gatica-Domínguez, Purnima Menon, and Robert E Black. Revisiting maternal and child undernutrition in low-income and middle-income countries: variable progress towards an unfinished agenda. Lancet.2021;397(10282):1388–1399. doi: 10.1016/S0140-6736(21)00394-9.
52. Watanabe K & Petri WA Jr. Environmental enteropathy: elusive but significant subclinical abnormalities in developing countries. *EBioMedicine*. 2016;10:25–32.
53. Clark S, Berrang-Ford L, Lwasa S. The burden and determinants of self-reported acute gastrointestinal illness in an Indigenous Batwa Pygmy population in southwestern Uganda. Epidemiol Infect. 2015;143(11):2287–98.
54. Patterson K, Berrang-Ford L, Lwasa S, Namanya DB, Ford J, Twebaze F, *et al.* Seasonal variation of food security among the Batwa of Kanungu, Uganda. Public Health Nutr. 2017;20(1):1–11.
55. Bogin B Scheffler C & Hermanussen M. Global effects of income and income inequality on adult height and sexual dimorphism in height. Am J Hum Biol. 2017; 29.e22980.
56. Perkins JM, Subramanian SV, Davey Smith G. Adult height, nutrition, and population health. Nutr Rev. 2016;74:149-165.
57. Silventoinen K. Determinants of variation in adult body height. J Biosoc Sci. 2003;35:263–285.
58. de Oliveira VH & Quintana-Domeque C. Early-life environment and adult stature in Brazil: an analysis for cohorts born between 1950 and 1980. Econ Hum Biol. 2014; 15:67–80.
59. National Institute of Population Research and Training, Mitra, and Associates, & ICF International (2014) Bangladesh Demographic and Health Survey 2014. Dhaka and Rockville, MD: NIPORT, Mitra and Associates, and ICF International.
60. Fudvoye J & Parent AS. Secular trends in growth. Ann Endocrinol (Paris). 2017;78:88-91.
61. Ken Maleta. Undernutrition. Malawi Medical Journal; 2006;18(4):189-205.
62. Eric Nzirakaindi Ikoona, Mame Awa Toure, Kassim Kamara, Freddy Wathum Drinkwater Oyat, Judith Aloyo, David Lagoro Kitara, *et al.* Double Burden of Malnutrition Among Women in Reproductive Age (15-49 years) in Sierra Leone: A Secondary Data Analysis of the Demographic Health Survey of 2019 (SLDH-2019). Research Square. 2022. DOI: 10.21203/rs.3.rs-1992723/v1.
63. Survey Quraish Sserwanja, Kassim Kamara, Linet M Mutisya, Milton W Musaba and Shirin Ziaei. Rural and Urban Correlates of Stunting Among Under Five Children in Sierra Leone: A 2019 Nationwide Cross-Sectional. Nutrition and Metabolic Insights. 2021;4:1–10.

Revised Manuscript with Track Changes

Prevalence and factors associated with undernutrition among 15–49-year-old women in Sierra Leone: A secondary data analysis of Sierra Leone Demographic Health Survey of 2019.

Nelson Onira Alema[1], Eric Nzirakaindi Ikoona[2], Mame Awa Toure[2], Oliver Eleeza[2], Amon Njenga[2], John Bosco Matovu[3] Lucy Namulemo[4,5,6], Ronald Kaluya[6], Kassim Kamara[7], Freddy Wathum Drinkwater Oyat[8], Emmanuel Olal[8,9], Judith Aloyo[8,10], David Lagoro Kitara[8,11,12*].

[1]Department of Anatomy, Faculty of Medicine, Gulu University, Gulu City, Uganda.
[2]ICAP at Columbia University, Freetown, Sierra Leone.
[3]ICAP at Columbia University, Nairobi, Kenya.
[4]Foothills Community Based interventions, Monticello, Kentucky, USA.
[5]Lindsey Wilson College, School of Professional Counseling, Kentucky, USA.
[6]Uganda Counseling and Support Services, Kampala, Uganda.
[7]Directorate of Health Security and Emergencies, Ministry of Health and Sanitation, Sierra Leone.
[8]Uganda Medical Association (UMA), UMA-Acholi branch, Gulu City, Uganda.
[9]Yotkom Medical Centre, Kitgum, Uganda.
[10]Rhites-N, Acholi, Gulu City, Uganda.
[11]Gulu Centre for Advanced Medical Diagnostics, Research, Trainings, and Innovations (GRUDI BIONTECH INITIATIVE), Gulu City, Uganda.
[*12]Gulu University, Faculty of Medicine, Department of Surgery, Gulu City, Uganda.

***Corresponding Author:** *David Lagoro Kitara is a Takemi fellow of Harvard University and Faculty at Gulu University, Faculty of Medicine, Department of Surgery, P.0. Box 166, Gulu City, Uganda, email: klagoro2@gmail.com; phone: (+256)772524474; ORCID: 0000-0001-7282-5026.*

Abstract

Background: Undernutrition of non-pregnant women of childbearing age is pertinent for maternal and offspring health. This study aimed to determine the prevalence and factors associated with undernutrition (underweight and stunting), among women of reproductive age (15-49 years) in Sierra Leone using secondary data analysis of the 2019 Demographic Health Survey.

Methods: Anthropometric measurements and maternal characteristics were obtained from the Sierra Leone Demographic Health Survey (SLDHS) of 2019. The heights and weights of women were measured, and BMI in kg/m^2 was calculated. Based on the World Health Organization's recommendations, stunting was defined as having heights <145cm, and underweight as BMI <18.5kg/m^2. Multivariable logistic regression analyses were conducted to identify correlates of undernutrition, with a significance level set at p<0.05.

Results: A total of 7,514 women of reproductive age, 15-49 years, participated in this study. The prevalence of stunting and underweight was 1.5% (113/7514) and 6.7%(502/7,514), respectively. Women with primary education had a 47% lower likelihood of stunting (adjusted Odds Ratio [aOR]=0.53, 95% Confidence Interval [CI]:0.30-0.94;p=0.029) than secondary education. Also, women in the poorest wealth index had a 51% lower likelihood of stunting (aOR=0.49,95%CI:0.27-0.88;p=0.017) than middle wealth index. However, underweight was 1.48 times more likely among women with a parity of one-to-four (aOR=1.48,95% CI:1.08-2.03;p=0.015) than women who had never given birth. Additionally, underweight was 1.41 times more likely among women who listened to radios (aOR=1.41,95% CI:1.14-1.74;p=0.002) than those who did not. Notably, age groups of 15-19 years and 40-49 years had a 54% (aOR=0.46,95%CI:0.34-0.62;p<0.001) and 34% (aOR=0.66,95%CI:0.45-0.97;p=0.035) lower likelihood of underweight than 20-29-year age group, respectively. Women with primary education had a 26% lower likelihood of underweight (aOR=0.74,95%CI:0.56-0.99;p=0.042) than secondary education. However, none of the wealth indices were significantly associated with underweight.

Conclusion: The prevalence of underweight and stunting among women of reproductive age (15-49 years) in Sierra Leone was lower compared to regional and world data. This study highlights similarities and differences in this population's prevalence and correlates of undernutrition. Underweight and stunting were less likely in women with primary education, while parity of one-to-four and listening to radios were significantly associated with underweight. Further trend studies using DHS data from 2010, 2014, and 2019 are warranted to understand the dynamics of undernutrition in Sierra Leone.

Keywords: Underweight, stunting, women of reproductive age (15-49 years), Sierra Leone, undernutrition.

Introduction: Malnutrition, characterized by deficiencies in calories, protein, vitamins, and minerals, coupled with poor health and social conditions, poses a significant health challenge for millions of women and adolescent girls worldwide[1]. Adequate nutrition is crucial for women's overall health and has far-reaching implications for the well-being of their children[1]. Children born to malnourished women are at higher risk of cognitive impairments, stunted growth, increased susceptibility to infections, and elevated morbidity and mortality rates throughout their lives[1].

Undernutrition remains a pressing global health issue, encompassing aspects such as being underweight, wasting, stunting, and deficiencies in essential minerals and vitamins[2]. Research indicates that women with a body mass index (BMI) below 18.5 kg/m^2 in developing countries face an escalating mortality risk and heightened vulnerability to illnesses[2-4]. Consequently, the impact of malnutrition extends beyond women's health, affecting the well-being of their children[5-6]. This perpetuates a cycle of undernutrition that spans generations[5,6], especially in countries like Sierra Leone where social and biological factors such as civil unrest, poverty, epidemic outbreaks, and food insecurity contribute to women's vulnerability to undernutrition[5]. In addition, numerous factors at individual, household, and community levels influence women's nutritional health status[5,6].

On the one side, stunting is a consequence of complex interactions among household, environmental, socioeconomic, and cultural factors, and has detrimental effects such as susceptibility to infections, impaired cognitive and motor development, and elevated risks of non-communicable diseases later in life[7-9]. Also, research has shown that individuals who experience stunting during childhood are more likely to face challenges such as poor cognitive function, lower educational performance, reduced adult wages, decreased productivity, and an increased risk of nutrition-related chronic diseases in adulthood[10]. Therefore, ensuring adequate nutrition serves as a fundamental foundation for individual and population health[11-16].

Furthermore, maternal undernutrition, underweight and stunting, has been linked to adverse maternal health conditions, such as chronic energy deficiency, caesarean delivery, pre-eclampsia, anemia, decreased productivity, and mental health issues, and adverse pregnancy outcomes[11-16]. On the other end of the malnutrition spectrum, overweight and obesity pose significant health risks for women, including a higher likelihood of developing hypertension, diabetes, cardiovascular diseases, and stroke[17–20].

Thus, determinants of undernutrition in women encompass many factors, including community-level water, sanitation, and hygiene (WASH) practices[21,22], food stability status[23], household income and wealth, women's education level, age at first marriage, age at first delivery, multiparity, short birth intervals, and land ownership[19,24-28]. Therefore, identifying the prevalence levels and determinants of maternal nutritional status is crucial for targeted interventions and resource allocation in resource-limited settings[19, 21-28].

Despite the significance of understanding maternal nutritional status, limited research has been conducted in Sierra Leone, often focusing solely on malnutrition determinants in young children and adolescents. The present study aims to address this research gap by investigating the risk factors for undernutrition among women of reproductive age (15-49 years) in Sierra Leone, utilizing data from the Sierra Leone Demographic Health Survey (SLDHS-2019).

The findings of this study hold important policy implications from a global health perspective and specifically for Sierra Leone, aiding in monitoring progress toward sustainable development goals (SDGs) and regional nutrition strategies. Moreover, the study can guide the allocation of limited resources by the government and health stakeholders to improve the nutritional and health status of women and infants in Sierra Leone.

In addition, in using data from a population-based cohort of non-pregnant women of childbearing age in Sierra Leone, this study aimed to determine the prevalence and factors associated with undernutrition (underweight and stunting) among women of reproductive age (15-49 years) based on the 2019 Demographic Health Survey.

Methods

Study design: The SLDHS-2019 was conducted as a countrywide representative cross-sectional survey, led by the Bureau of statistics of Sierra Leone (Stats SL) with technical assistance from ICF through DHS programs. This survey was funded by the United States Agency for International Development (USAID)[29].

Study sites: This study was conducted in all the four provinces and western areas of Sierra Leone[29].

Sampling and study participants: Sampling of the study participants was based on the 2015 population and housing census of the Republic of Sierra Leone[30]. This census was conducted by Statistics Sierra Leone (Stats SL) and provided the ready-made sampling frame for the SLDHS-2019[30].

Sierra Leone is administratively divided into four provinces and western areas (urban and rural), sixteen districts, and 190 chiefdoms[30,31,32]. Each district is subdivided into chiefdoms/census wards, and each chiefdom/census ward is subdivided into sections[30,31,32]. In addition, the 2015 population and housing census subdivided each locality into

convenient census: the enumeration areas (EAs)[30,33]. The EAs were the primary sampling units (PSUs) and clusters for the SLDHS-2019[30-35]. The list of EAs from the 2015 census formed the basis for estimating the number of households required and for classifying EAs (clusters) into urban/rural for the SLDHS-2019 sampling frame[30,31,34,35]. Furthermore, the SLDHS-2019 employed a two-stage stratified sampling design and the stratification was achieved by classifying each district into urban and rural areas[34,35]. So, thirty-one sampling strata were created, and samples were selected independently in each stratum via a two-stage selection process[34,35].

Thus, implicit stratifications were achieved at each lower administrative level by sorting the sampling frame before sample selection according to administrative order and using a probability proportional-to-size selection during the first sampling stage[34,35].

Also, five hundred and seventy-eight (578) EAs were selected using a probability proportional to EA size[34,35] in the first stage of the selection process. In addition, the enumeration area size was determined by the number of households residing in it and a household listing operation was then performed in all selected enumeration areas[34,35]. The resulting lists of households served as a sampling frame for selecting households in the second stage of the survey[34,35].

In the second stage's selection, a fixed number of twenty-four households was chosen in every cluster through an equal probability systematic sampling, resulting in a total sample size of approximately 13,872 households distributed in 578 clusters[34,35]. The household listing in this stage was conducted using computer tablets, and households were randomly selected through computer programming[34,35].

The survey interviewed only pre-selected households in the clusters, and no replacements or changes of the selected households were allowed in the implementation stage of the survey to prevent selection bias of the study population[34,35]. Due to non-proportional allocation of samples to the sixteen districts in Sierra Leone and the possible differences in response rates, sample weights were calculated, added to the data file, and applied so that the results would be representative at national and domain levels[34,35]. Further, because the SLDHS-2019 sample was a two-stage stratified cluster sampling, sample weights were calculated separately at each sampling stage based on sampling probabilities[34,35]. Thereafter, the SLDHS-2019 included all women aged 15-49 in the sampled households[34,35]. Permanent residents in the selected homes and visitors who stayed overnight before the survey were eligible for interviews in the household[34,35]. The man's questionnaire covered the identification of

respondents, background information, reproduction, contraception, marriage and sexual activity, fertility preferences, employment status, gender roles, HIV and AIDS, and other health issues[35]. The biomarker questionnaire covered the identification of respondents, weights, heights, and hemoglobin measurements for children aged 0–5 years, weights, heights, HIV testing, and hemoglobin measurements for women aged 15–49 years[35]. The fieldworker questionnaire covered the background information on each field worker[35].

Anthropometric measurements. The weight of respondents was recorded in kilograms (kg) to the nearest decimal point and was measured using an electronic scale (SECA 878)[34,35]. Participants' heights were measured using a stadiometer in centimeters (cm) to one decimal point[34,35]. Body Mass Index (BMI) of respondents was calculated in kg/m^2 using weights (in kilograms) and heights (meters) of women of reproductive age (15–49 years) and classified according to WHO criteria as underweight ($<18.5 kg/m^2$), normal weight ($18.5–24.9 kg/m^2$), overweight ($25.0–29.9 kg/m^2$), obesity ($\geq 30.0 kg/m^2$ and $\leq 50.0 kg/m^2$), and overnutrition ($\geq 25.0 kg/m^2$ and $\leq 50.0 kg/m^2$).

Wealth Index (WI). To calculate each household's wealth, we used wealth index (WI) as a proxy indicator of household wealth[35]. This composite index used household key asset ownership variables to calculate each household wealth index from the SLDHS-2019 data[35]. These variables were the characteristics of the household's dwelling unit, for example, the source of water, type of toilet facilities, type of fuel used for cooking, number of rooms, ownership of livestock, possessions of durable goods, mosquito nets, and primary materials for the floor, roof, and walls of the dwelling place[35]. The respondent's household wealth index was calculated using computer analysis of household composite factors. It was then categorized into five quintiles as poorest, poorer, middle, richer, and richest wealth indices (Table 1).

Operational definitions.

Body Mass Index (BMI): Weight in kilograms divided by heights in meters squared (kg/m^2).

Underweight: BMI $<18.5 kg/m^2$

Overweight: BMI $\geq 25.0 kg/m^2$ and $\leq 29.9 kg/m^2$

Obese: BMI $\geq 30.0 kg/m^2$ and $\leq 50.0 kg/m^2$

Overnutrition (Overweight and obese): BMI $\geq 25.0 kg/m^2$ and $\leq 50.0 kg/m^2$.

Enumeration Area (Clusters): An EA is a geographic area consisting of a convenient number of dwelling units that served as a counting unit for the survey.

Data Collection: Data collection for this survey was conducted from May 14, 2019, to August 31, 2019[29]. The primary sampling unit (PSU), referred to as a cluster, was based on enumeration areas (EAs) obtained from the 2015 EA population census sampling frame[29].

The SLDHS-2019 used five validated questionnaires for the thematic parts covered in the survey[29]. The household questionnaire collected data on household environment, assets, and basic demographic information of household members. The woman's questionnaire collected data on women's reproductive health, domestic violence, and nutrition indicators[29]. The man's questionnaire collected data on men's health, while the biomarker questionnaire collected data on anthropometry and blood tests for mothers and children (0-5 years), and the fieldworker questionnaire collected data on background information of fieldworkers[36,37].

This secondary data analysis included women in the reproductive age, 15-49 years, whose anthropometric characteristics were recorded with their consent. Trained health technicians were deployed to measure the heights and weights of the participants to ensure the quality of anthropometric measurements[29].

Out of the weighted sample of 15,934 women in the dataset, 7,514 anthropometric measurements were included in the survey design, while 8,420 had invalid weight measurements due to erroneous, and ineligible measurements. The weight measurement is important for calculating the BMI of each participant which was finally used for the assessing the nutritional status of each respondent.

In some of the results of participants, heights too were not well recorded, and we were only able to obtain completed anthropometric measurements for 7,514 women who were not lactating, non-pregnant and post-menopausal. In the final analysis, weighted sample of 7,514 was included in our secondary data analysis as summarized in Table 1. A full protocol with detailed explanations about data collection processes and sampling is available online[29].

Outcome variables: The first outcome variable for this study was stunting. It was coded as "1" for stunted women and "0" for not stunted. Stunting was defined as heights of <145cm ± Standard Deviations (SD) from the median value set by the World Health Organization (WHO). The second outcome variable was underweight, which was defined as BMI<18.5kg/m^2 and coded as "1" for underweight women and "0" for normal weight. Normal weight was defined as BMI of 18.5-24.9kg/m^2.

Independent variables: The independent variables in this study were based on previous studies, the WHO stunting framework, underweight, normal weight, and available information in the SLDHS-2019 database. We included nineteen independent variables in this data analysis.

Women's characteristics: Parity (categorized as para 0, para one-to-four, and five and above), work status (categorized as working-class versus not working), marital status (categorized as married versus not married/single), levels of education (categorized as no education, primary, secondary, and higher), age groups (categorized as 15-19, 20-29, 30-39, and 40-49 years), woman's stunting status (defined as heights <145cm for stunted and ≥145cm for not stunted women), and woman's BMI classification as normal BMI (18.5-24.9kg/m^2) and underweight (<18.5kg/m^2).

Household characteristics: These characteristics include regions of Sierra Leone (Northwest, Eastern, Western, Southern, and Northern); household wealth indices (categorized as richest, richer, middle, poorer, and poorest); sex of the head of household (female versus male); household size (less than six versus six and above); residency (urban versus rural); television viewing (yes versus no); reading magazines (yes versus no); listening to radios (yes versus no); smoking cigarettes (yes versus no); and alcohol use (yes versus no).

Ethical approval: This survey protocol was approved by the Sierra Leone Ethics and Scientific Review Committee (SLESRC) and the ICF Institutional Review Board. For each adult participant, written informed consent was obtained, and for participants under eighteen years, an assent was obtained in the presence of a guardian or a legal representative.

Statistical analysis: Frequency tables and proportions/percentages were used to describe summaries of categorical variables, while means and standard deviations (±SD) were used for continuous variables. Sample weights were used to account for unequal probability sampling in different strata of the study population and to ensure representativeness of the survey results at all levels[29]. Statistical software SPSS version 25.0 statistical software complex samples package incorporating all variables in the analysis plan was used to account for the multistage sampling design inherent in the DHS dataset, including individual sample weight, sample strata for sampling errors/design, and cluster numbers[38-40].

The use of complex samples package ensured that the sampling design was incorporated into the analysis, leading to accurate and reliable results[38-40]. Cross tabulations were conducted, and associations between socio-

demographic characteristics and women's nutritional status (stunting and underweight), including their Odds ratios (OR) and P-values, were presented in Table 2 and Table 3.

To assess associations of each independent variable with dependent variables (stunting and underweight), a bivariate logistic regression analysis was conducted, and Crude Odds Ratios (COR), at 95% Confidence Intervals (CI), and P-values were presented. Independent variables found significant at bivariable level and those with P-values ≤0.20 were included in the final multivariable logistic regression analysis model for each dependent variable. Variables that had P-values above 0.201 at bivariable level were excluded from the final regression model.

Adjusted Odds Ratios (aOR), at 95% Confidence Intervals (CI), and corresponding P-values were calculated, with statistical significance levels set at 0.05.

Sensitivity analysis: Sensitivity analysis for stunting was conducted by excluding women with parity of five and above in the multivariable logistic regression model, as it had only 23(1.3%) stunted women. By excluding them from the final regression model, the other factors remained significant, and no substantial changes were observed in the strength of associations. The same statistical approach was used for studying underweight in this study population. Cross tabulations were conducted, and associations between socio-demographic characteristics and women's nutritional status (underweight versus normal weight), including their aOR at 95% CI and P-values, were presented (in Table 3), with no significant differences observed after excluding women with BMI ≥25.0kg/m^2.

Results

The study was a demographic health survey conducted in Sierra Leone and includes 7,514 women in the reproductive age, 15-49 years (Table 1). Among the women, the majority belonged to the 20-29-year age-group, accounting for 33.6%(2528/7514) of the total population. Women with parity ranging from one to four represented just over 50%(51.8%; 3892/7514) of the total study population, while most participants resided in rural areas 58.9%(4,422/7,514) of Sierra Leone. Male-headed households constituted slightly over two thirds of the study population at 71.3%(5,356/7,514). Moreover, households with a size of six or more individuals constituted the majority at 60.1%(4,519/7,514).

Most women in the study population were working-class women, representing 69.7%(5,234/7,514). Among marital status categories, married women constituted the majority at 63.8%(4,795/7,514). Regionally, women from the south of Sierra Leone constituted the largest proportion at 24.4%(1,831/7,514), followed by the north at

24.2%(1,822/7,514), the east at 21.0%(1,579/7,514), the west at 16.7%(1,256/7,514), and the northwest at 13.7%(1,026/7,514).

In terms of educational level, the majority had no formal education, accounting for 47.5%(3,571/7,514), followed by secondary education at 35.2%(2,641/7,514), primary education at 13.5%(1,017/7,514), and a smaller proportion with higher education at 3.8%(285/7,514) (Table 1).

Regarding wealth indices, 21.7%(1,634/7,514) of women were in the richer wealth index category followed by poorest and middle at 20.4%(1,533/7,514) each, poorer wealth index at 19.0%(1,482/7,514), and the smallest proportion among the richest wealth index at 18.5%(1,388/7,514) (Table 1).

In terms of BMI categories, most women had normal BMI, accounting for 66.2%(4,974/7,514), followed by overweight at 19.7%(1,479/7,514), obese at 7.4%(5,59/7,514) and the smallest proportion among underweight women at 6.7%(502/7,514) (Table 1).

In terms of social activities, several participants did not watch television, accounting for 74.9%(5,625/7,514). Furthermore, most participants did not listen to radios 58.2%(4,372/7,514) and did not read magazines 93.5%(7,025/7,514). Additionally, most women in the reproductive age did not smoke cigarettes 97.0%(7,290/7,514), and did not use alcohol 41.0%(3,081/7,514) (Table 1).

The prevalence of stunting: Out of a total of 15,574 women in the SLDHS-2019, 48%(7,514/15,574) had valid height measurements. The mean height was 157.6cm with a standard deviation (SD) of ±6.3cm. The minimum height recorded was 107.7cm, and the maximum height was 186.2cm. The overall prevalence of stunting in the study population was 1.5%(113/15,574) (Table 2).

Figure 1: Heights of women in the reproductive age (15-49 years) in Sierra Leone.

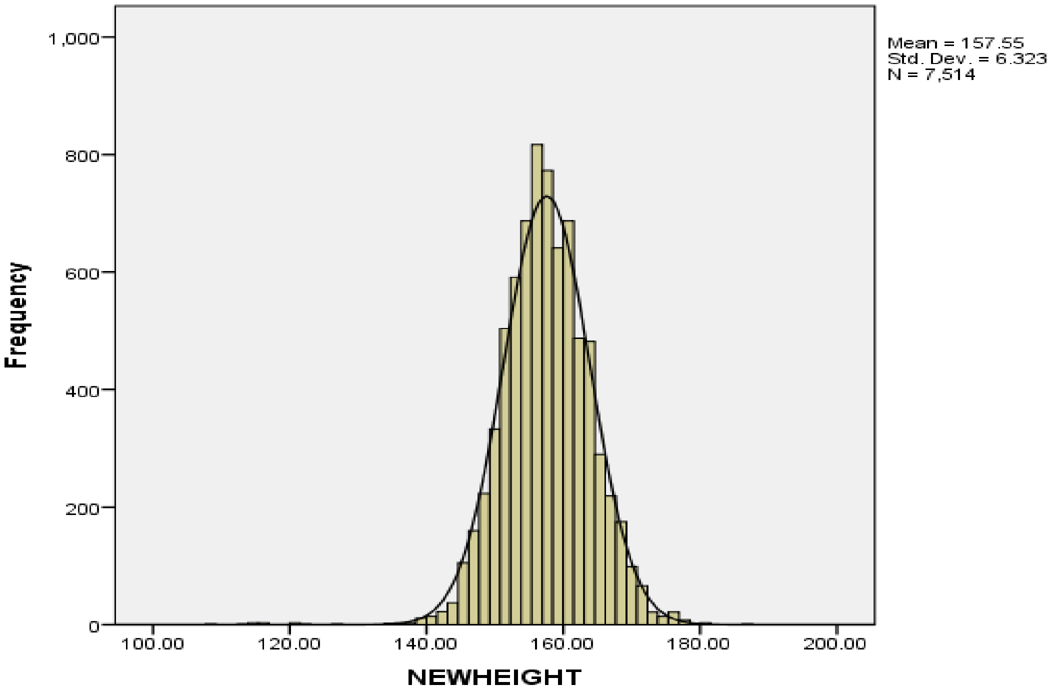

The source of data is SLDHS of 2019.

Figure 1 shows a normally distributed heights among women aged (15-49) years in Sierra Leone. The mean height was 157.6 cm, SD±6.3.

The prevalence of underweight: Among the total population of women (n=7,514), the mean BMI was 23.8kg/m^2 (SD±4.7). The prevalence of underweight was 6.7%(502/7,514), with a minimum BMI recorded at 12.8kg/m^2. Within the underweight category, there were two outlier BMI values, namely 12.8kg/m^2 and 14.5kg/m^2, each representing 0.03% of the total study population. These outlier BMIs were situated on the left side of the normal distribution curve (Figure 1) (Table 2).

Figure 2: Frequency of underweight among age-groups of women (15-49 years) in the 2019 SLDHS.

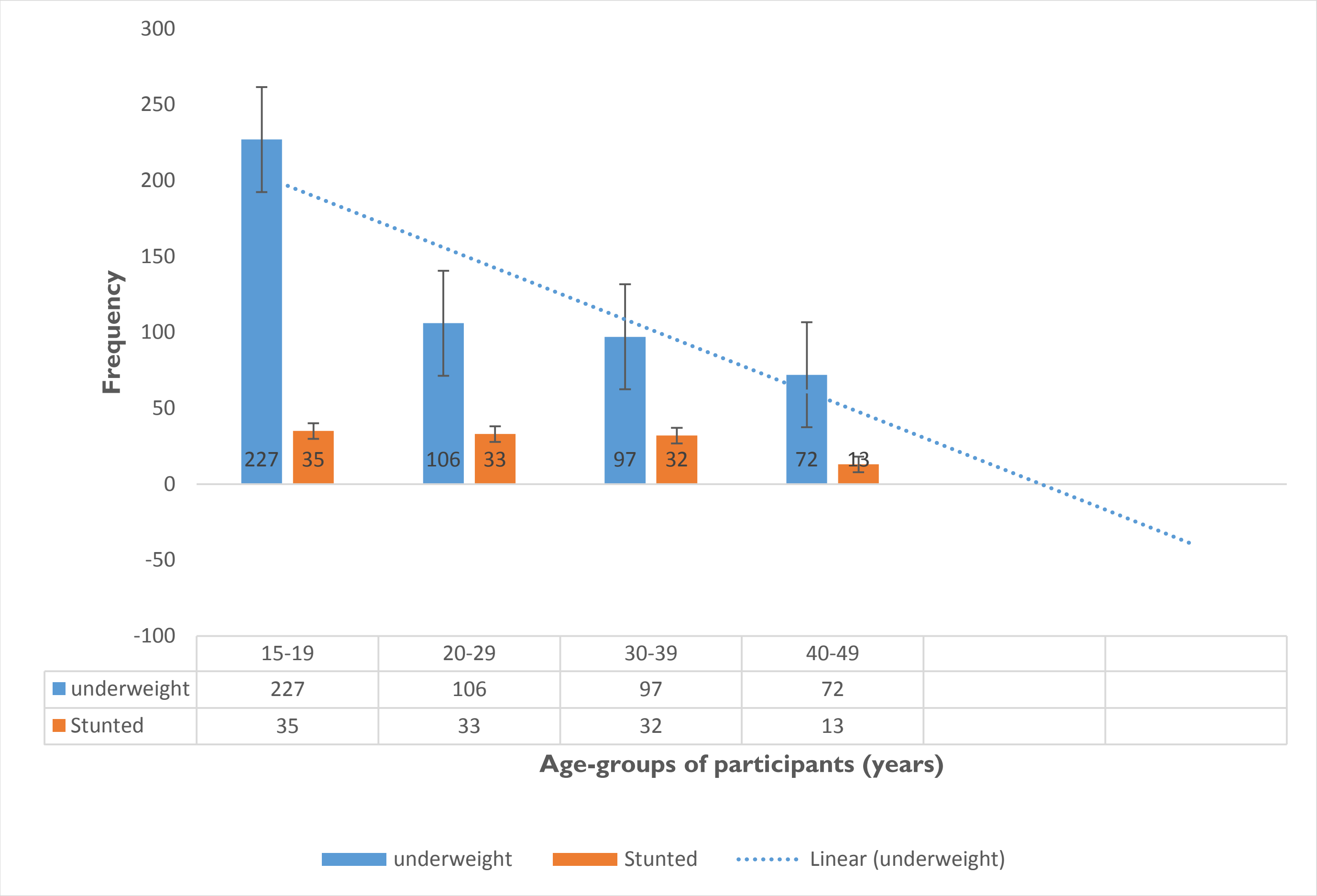

The source data is SLDHS of 2019.

Figure 2 shows the frequency of underweight as it decreased with age group populations with the majority in the 15-19-year age group 45.2%(227/502), followed by 20-19-year age group 21.1%(106/502); 30-39-year age group 19.3%(97/502), and least among the 40-49-year age group 14.3%(72/502).

Correlates of stunting among women of reproductive age (15-49 years) in Sierra Leone: The study revealed that a primary level of education and belonging to the poorest wealth index were less likely factors of stunting among the study population. Women with a primary level of education had a 47% lower likelihood of being stunted (aOR=0.53,95%CI:0.30-0.94;p=0.029) than those with a secondary education. Similarly, women in the poorest wealth index had a 51% lower likelihood of being stunted (aOR=0.49,95%CI:0.27-0.88;p=0.017) than in the middle wealth index. Other factors such as parity, residence (urban or rural), sex of the household head, household size, work status, marital status, regions of residence, listening to radios, reading of magazines, alcohol use and smoking cigarettes did not significantly affect the occurrence of stunting among study participants (Table 2).

Correlates of underweight among women (15-49 years) in Sierra Leone. After adjusting for individual characteristics in the final multivariable logistic regression model, the determinants of underweight among Sierra Leonean women of reproductive age (15-49 years) were identified: Women with a parity of one-to-four had a

1.48 times higher likelihood of being underweight (aOR=1.48, 95%CI:1.08-2.03; p=0.015) compared to those who never gave birth. In addition, women who listened to radios had a 1.41 times higher likelihood of being underweight (aOR=1.41,95%CI:1.14-1.74; p=0.002) than those who did not. However, being in the age group of 15-19 years was associated with a 54% lower likelihood of underweight (aOR=0.46, 95% CI:0.34-0.62; p<0.001) than the 20-29-year age-group and being in the age group of 40-49 years was associated with a 34% lower likelihood of underweight (aOR=0.66, 95%CI:0.45-0.97; p=0.035). Furthermore, having a primary level of education was associated with a 26% lower likelihood of underweight (aOR=0.74, 95% CI: 0.56-0.99; p=0.042) than secondary level of education. None of the wealth indices showed a significant association with underweight in this study population (Table 3).

Table 1: Socio-economic and demographic characteristics of women in the reproductive age (15-49 years) in Sierra Leone.

Variables	Frequency (N=7,514)	Percent (%)
**Ages (years)**		
15-19	1,616	21.5
20-29	2,528	33.6
30-39	2,048	27.3
40-49	1,322	17.6
**Parity**		
Never gave birth	1,895	25.2
One to four	3,892	51.8
Five and above	1,727	23.0
**Type of residence**		
Urban	3,092	41.1
Rural	4,422	58.9
**Sex of the head of household**		
Male	5,356	71.3
Female	2,158	28.7
**Household size**		
Less than six	2,995	39.9
Six and above	4,519	60.1
**Work status**		
Not working	2,280	30.3
Working	5,234	69.7
**Marital status**		
Married	4,795	63.8
Not married	2,719	36.2
**Regions of Sierra Leone**		
East	1,579	21.0
North	1,822	24.2
Northwest	1,026	13.7
South	1,831	24.4
Western	1,256	16.7
**Levels of education**		
No formal education	3,571	47.5
Primary	1,017	13.5
Secondary	2,641	35.2
Higher	285	3.8
**Wealth Indices**		
Poorest	1,533	20.4
Poorer	1,428	19.0
Middle	1,531	20.4
Richer	1,634	21.7
Richest	1,388	18.5
**BMI categories (kg/m^2)**		
Underweight (<18.5)	502	6.7
Normal weight (18.5-24.9)	4,974	66.2
Overweight (25.0-29.9)	1,479	19.7
Obese (≥30.0)	559	7.4
**Watching Television**		
Yes	1,889	25.1
No	5,625	74.9
**Listening to radios**		
Yes	3,142	41.8
No	4,372	58.2
**Reading of magazines**		
Yes	489	6.5
No	7.025	93.5
**Smoking of cigarettes**		
Yes	224	3.0
No	7,290	97
**Alcohol use**		
Yes	667	8.9
No	3,081	41.0

The data source is SLDHS-2019.

In Table 1 the majority of women of reproductive age (15-49 years) in Sierra Leone were in the 20-29-year age group 2528/7514(33.6%); parity of one-to-four 3892/7514(51.8%); of rural residence 4422/7514(58.9%); male-headed households 5356/7514(71.3%); household size of six and above 4519/7514(60.1%); working class 5234/7514(69.7%); married 4,795/7514(63.8%); from the South 1831/7514(24.4%); had no formal education 3571/7514(47.5%); richer wealth index 1634/7514(21.7%); normal weight 4974/7514(66.2%); did not watch television 5625/7514(74.9%); did not listen to radios 4372/7514(58.2%); did not read magazines 7025/7514(93.5%), did not smoke cigarettes 7290/7514(97.0%), and did not use alcohol 3081/7514(41.0%).

Table 2: Bi- and multivariable analysis of stunting among women (15-49 years) in SLDHS-2019.

Variables	Stunted (n=113) (n, %)	Not stunted (n=7,401) (n, %)	Unadjusted POR	95% CI	p value	aOR	95% CI	p value
**Age groups (years)**								
20-29	33(1.3)	2495(98.7)	**Reference**			**Reference**		
15-19	35(2.2)	1582(97.8)	0.597	0.370-0.965	0.035	0.815	0.437-1.520	0.419
30-39	32(1.6)	2016(98.4)	0.833	0.511-1.360	0.465	0.936	0.533-1.644	0.520
40-49	13(1.0)	1309(99.0)	1.332	0.699-2.539	0.384	1.559	0.741-3.277	0.818
**Parity**								
Never gave birth	38(2.0)	1857(98.0)	**Reference**			**Reference**		
One to four	52(1.3)	(3840(98.7)	1.511	0.991-2.304	0.055	1.489	0.792-2.801	0.216
Five and above	23(1.3)	1704(98.7)	1.516	0.900-2.555	0.118	1.524	0.659-3.524	0.324
**Residence**								
Rural	80(1.8)	4342(98.2)	**Reference**			**Reference**		
Urban	33(1.1)	3059(98.9)	1.708	1.136-2.569	0.010	1.257	0.614-2.572	0.531
**Sex of the household head**								
Male	76(1.4)	5280(98.6)	**Reference**					
Female	37(1.7)	2121(98.3)	0.825	0.555-1.226	0.342			
**Household size**								
Six and above	59(1.3)	4460(98.7)	**Reference**			**Reference**		
Less than six	54(1.8)	2941(98.2)	0.72	0.497-1.045	0.084	0.761	0.518-1.112	0.166
**Work status**								
Not working	33(1.4)	2247(98.6)	**Reference**					
Works	80(1.5)	5154(98.5)	0.946	0.946-1.424	0.791			
**Marital status**								
Not married	48(1.8)	2671(98.2)	**Reference**			**Reference**		
Married	65(1.4)	4730(98.6)	1.308	0.898-1.905	0.162	1.303	0.763-2.224	0.333
**Region of residence**								
East	24(1.5)	1555(98.5)	**Reference**			**Reference**		
North	25(1.4)	1797(98.6)	1.109	0.631-1.950	0.718	1.167	0.656-2.074	0.600
Northwest	8(0.8)	1018(99.2)	1.964	0.879-4.389	0.100	1.908	0.846-4.302	0.119
South	43(2.3)	1788(97.7)	0.642	0.388-1.062	0.085	0.695	0.415-1.162	0.165
Western	13(1.0)	1243(99.0)	1.476	0.748-2.910	0.261	0.821	0.368-1.831	0.630
**Level of education**								
Secondary	32(1.2)	2609(98.8)	**Reference**			**Reference**		
No formal education	56(1.6)	3515(98.4)	0.770	0.497-1.192	0.241	0.624	0.351-1.107	0.107
Primary	25(2.5)	992(97.5)	0.487	0.287-0.825	0.008	0.531	0.300-0.938	0.029
Higher	0	285(100)	19814180	0	0.994	12203543	0	0.994
**Wealth Indices**								
Middle	17(1.1)	1514(98.9)	**Reference**			**Reference**		
Poorest	41(2.7)	1492(97.3)	0.409	0.231-0.722	0.002	0.485	0.268-0.880	0.017
Poorer	21(1.5)	1407(98.5)	0.752	0.395-1.432	0.386	0.778	0.404-1.497	0.452
Richer	24(1.5)	1610(98.5)	0.753	0.403-1.408	0.374	0.654	0.302-1.417	0.282
Richest	10(0.7)	1378(99.3)	1.547	0.706-3.391	0.275	1.068	0.377-3.026	0.902
**Watching television**								
No	96(1.7)	5529(98.3)	**Reference**			**Reference**		
Yes	17(0.9)	1872(99.1)	1.912	1.139-3.211	0.014	1.385	0.744-2.578	0.304
**Listens to radio**								
No	74(1.7)	4298(98.3)	**Reference**			**Reference**		
Yes	39(1.2)	3103(98.8)	1.37	0.927-2.204	0.114	0.902	0.585-1.392	0.642
**Reading of magazines**								
No	108(1.5)	6917(98.5)	**Reference**					
Yes	5(1.0)	484(99.0)	1.511	0.614-3.722	0.369			
**Smokes cigarettes**								
No	109(1.5)	7181(98.5)	**Reference**					
Yes	4(1.8)	220(98.2)	0.835	0.305-2.285	0.725			
**Alcohol use**								
No	45(1.5)	3036(98.5)	**Reference**					
Yes	8(1.2)	659(98.8)	1.221	0.573-2.602	0.605			

aOR: adjusted Odds Ratio; CI: Confidence Interval; COR: Crude Odds Ratio; SLDHS: Sierra Leone Demographic and Health Survey.

In Table 2, the determinants of stunting among Sierra Leone women of reproductive age were less likely among women of primary level of education, aOR=0.53,95%CI:0.30-0.94;p=0.029 and those in the poorest wealth index aOR=0.49,95%CI:0.27-0.88; p=0.017.

Table 3: Prevalence and determinants of underweight among women (15-49 years) in SLDHS-2019.

Variables	Under-weight (N=502) (n, %)	Normal weight (N=4,974) (n, %)	Unadjusted (COR)	95% CI	p value	Adjusted (aOR)	95% CI	p value
**Age groups (years)**								
20-29	106(5.6)	1,773(94.4)	**Reference**			**Reference**		
15-19	227(16.0)	1,192(84.0)	0.314	0.246-0.400	<.001	0.457	0.335-0.624	<.001
30-39	97(7.2)	1,244(92.8)	0.767	0.577-1.019	0.068	0.746	0.536-1.037	0.081
40-49	72(8.6)	765(91.4)	0.635	0.465-0.867	0.004	0.663	0.453-0.972	0.035
**Parity**								
Never gave birth	225(14.5)	1,330(85.5)	**Reference**			**Reference**		
One to four	182(6.7)	2537(93.3)	2.358	1.918-2.899	<.001	1.479	1.079-2.029	0.015
Five and above	95(7.9)	1,107(92.1)	1.971	1.531-2.538	<.001	1.362	0.876-2.117	0.170
**Residence**								
Rural	340(9.7)	3,156(90.3)	**Reference**					
Urban	162(8.2)	1,818(91.8)	1.209	0.994-1.470	0.057			
**Sex of household head**								
Male	343(8.7)	3,621(91.3)	**Reference**			**Reference**		
Female	159(10.5)	1,353(89.5)	0.806	0.661-0.983	0.033	0.866	0.701-1.071	0.186
**Household size**								
Six and above	321(9.7)	2,998(90.3)	**Reference**					
Less than six	181(8.4)	1,976(91.6)	1.169	0.966-1.415	0.109			
**Work status**								
Not working	191(11.1)	1,529(88.9)	**Reference**			**Reference**		
Working	311(8.3)	3,445(91.7)	1.384	1.144-1.673	0.001	1.011	0.800-1.277	0.928
**Marital status**								
Not Married	270(12.6)	1,872(87.4)	**Reference**			**Reference**		
Married	232(7.0)	3,102(93.0)	1.928	1.603-2.319	<.001	1.251	0.936-1.672	0.130
**Region of residence**								
East	96(8.1)	1,082(91.9)	**Reference**			**Reference**		
North	153(10.5)	1,305(89.5)	0.757	0.579-0.989	0.041	0.765	0.581-1.008	0.057
Northwest	73(9.2)	724(90.8)	0.88	0.640-1.210	0.431	0.898	0.648-1.243	0.515
South	134(10.3)	1,173(89.7)	0.777	0.590-1.022	0.071	0.789	0.595-1.045	0.098
Western	46(6.2)	690(93.8)	1.331	0.925-1.916	0.777	1.248	0.823-1.892	0.298
**Level of education**								
Secondary	185(9.5)	1,755(90.5)	**Reference**			**Reference**		
No formal education	211(8.1)	2,399(91.9)	1.199	0.975-1.474	0.086	0.886	0.662-1.186	0.417
Primary	96(12.3)	686(87.7)	0.753	0.580-0.979	0.034	0.742	0.557-0.989	0.042
Higher	10(6.9)	134(93.1)	1.413	0.730-2.733	0.305	0.677	0.338-1.357	0.272
**Wealth Indices (WI)**								
Middle	121(10.3)	1,050(89.7)	**Reference**			**Reference**		
Poorest	104(8.3)	1,156(91.7)	1.281	0.973-1.666	0.078	1.236	0.929-1.646	0.146
Poorer	120(10.2)	1,053(89.8)	1.011	0.775-1.320	0.935	0.935	0.711-1.229	0.630
Richer	97(9.1)	974(90.9)	1.157	0.874-1.533	0.309	1.150	0.850-1.557	0.365
Richest	60(7.5)	741(92.5)	1.423	1.030-1.967	0.032	1.158	0.782-1.713	0.464
**Watching television**								
No	404(9.5)	3,851(90.5)	**Reference**					
Yes	98(8.0)	1,123(92.0)	1.202	0.955-1.514	0.117			
**Listens to radio**								
No	350(10.4)	3,007(89.6)	**Reference**			**Reference**		
Yes	152(7.2)	1,967(92.8)	1.506	1.235-1.837	<.001	1.407	1.136-1.742	0.002
**Reading magazines**								
No	473(9.1)	4,698(90.9)	**Reference**					
Yes	29(9.5)	276(90.5)	0.958	0.646-1.421	0.832			
**Smokes cigarettes**								
No	484(9.1)	4,835(90.9)	**Reference**					
Yes	18(11.5)	139(88.5)	0.773	0.469-1.274	0.313			
**Alcohol use**								

No	140(6.7)	2,005(93.3)	**Reference**		
Yes	35(7.5)	429(92.5)	0.856	0.582-1.258	0.428

aOR: adjusted Odds Ratio; CI: Confidence Interval; COR: Crude Odds Ratio; SLDHS: Sierra Leone Demographic and Health Survey.

In Table 3, the determinants of underweight among Sierra Leone women was likely among women with parity of one-to- four aOR=1.48,95%CI:1.08-2.03;p=0.015 and those who listened to radios, aOR=1.41,95%CI:1.14-1.74;p=0.002. However, underweight was less likely among age-group of 15-19 years, aOR=0.46,95%CI:0.34-0.62;p<0.001; age-group of 40-49 years, aOR=0.66,95%CI:0.45-0.97;p=0.035, and those with primary level of education, aOR=0.74,95%CI:0.56-0.99;p=0.042.

Discussion: This population-based study provides valuable insights into the prevalence and correlates of underweight and stunting among women of reproductive age (15-49 years) in Sierra Leone (Table 1, Figure 1, and Figure 2). The prevalence of stunting in Sierra Leone, at 1.5%, is higher than that reported in the DHS of Kenya (less than 1%)[41], and Uganda (1.3%)[36,42] but lower than Tanzania (less than 3%)[43].

Stunting among women of reproductive age is a significant concern, as it reflects long-term exposure to inadequate nutrition, infection, and environmental stress[44]. The consequences of stunting are far-reaching, particularly for girls and women of reproductive age[45], and the effects are experienced at individual, community, and national levels[46]. It is alarming to note that an estimated 450 million adult women in developing countries are stunted due to malnutrition during childhood[47].Therefore, addressing stunting among women is crucial for improving maternal and child health outcomes.

Stunting among women of reproductive age (15-49 years) in Sierra Leone from the 2019 DHS.

Women with primary level of education were 47% less likely to be stunted compared to those with secondary level of education. Similarly, women in the poorest wealth index were 51% less likely to be stunted compared to those in the middle wealth index. These findings highlight the importance of education and socioeconomic status in mitigating the risk of stunting among women. However, no other factors were found to be significantly associated with stunting in this study population (Table 2).

In contrast, the correlates of being underweight differed from those of stunting (Table 2 and Table 3). Parity of one-to-four and listening to radios were identified as significant factors associated with underweight (Table 2). Women with a parity of one-to-four were 1.48 times more likely to be underweight compared to those who had never given birth. (Table 3). On the other hand, age-groups of 15-19 years and 40-49 years, as well as primary

education, were less likely of being underweight. These findings suggest that different factors contribute to underweight compared to stunting among women (15-49 years) in Sierra Leone (Table 3)[48].

The underlying reasons for the low likelihood of primary level of education on both stunting and underweight in the study population remain unclear, highlighting the need for in-depth exploration through qualitative research. Conducting qualitative studies would allow for a deeper understanding of the factors and mechanisms that contribute to the observed association between primary level of education and better nutritional outcomes.

By delving into the lived experiences and socio-cultural context of women, qualitative research can provide valuable insights to unravel the complex dynamics at play. Further investigation through qualitative research is warranted to gain a comprehensive understanding of why primary level of education emerges as a protective factor against stunting and underweight in this population.

It is interesting to note that women in the poorest wealth index were less likely to be stunted compared to women in the middle wealth index (Table 2). This finding contradicts many studies conducted in other African countries where stunting is more prevalent among women in the poorest wealth indices[36,42,49].

Studies on stunting among children in Sierra Leone from the same SLDHS-2019 shows a high prevalence of stunting among children below five years[50]. However, our findings that women in reproductive age-group (15-49 years) from the same data source (SLDHS-2019) had no likely association with any age-group was unique while children below five years experienced high prevalence of stunting (31.6% in rural versus 24.0% in urban areas)[50].

The unique finding in Sierra Leone necessitates further investigation to explore the underlying factors contributing to this difference. It is plausible that low-income households have adopted favorable eating habits and practices, such as the consumption of locally available foods like *plasas*. *Plasas*, a mixture of green leaves with palm oil and fish, is not only affordable but also highly nutritious. Understanding the dietary choices and affordability of nutritious foods among low-income households could provide valuable insights into the observed findings.

Stunting is a chronic condition that begins during the prenatal period and persists through early childhood and adolescence, with the first two years of life being particularly critical[43,49]. Previous studies have highlighted the high prevalence of stunting among women of reproductive age in low-to-middle income countries, as stunted children often continue to experience stunting into adulthood[50,51]. However, it is important to note that some individuals who were stunted in childhood managed to overcome these challenges by accessing education, obtaining better

employment opportunities, increasing their income, or marrying into higher socioeconomic strata. As a result, they may have transitioned from lower to higher wealth indices, indicating the potential for social mobility and improvement in their overall well-being. This socioeconomic progress achieved by these women may have played a role in the observed outcome of low socio-economic status being unlikely for undernutrition.

In addition, many studies show that the use of improved drinking-water was associated with lower risk of stunting and that improved water was a proxy for less exposure to enteric pathogens[52]. Watanabe and Petri discussed that environmental enteropathy is a chronic disease caused by continuous exposure to faecally contaminated food and water that does not produce symptoms but contributes to poor physical development[52]. This may have been a factor experienced among populations in other countries but not in Sierra Leone.

These findings on stunting among women in Sierra Leone contrasts with another in Uganda where the population in Southwestern northeastern (pygmies and Batwa) were found to be naturally shorter compared to the average Ugandan population[53,54,55]. More to this could be explained by genetic factors which play part at individual level where it is likely that women in the reproductive age in Sierra Leone were generally taller because of their genetic makeup[12]. A contrasting scenario was observed in western Uganda among the pygmies and others who were generally shorter compared to the average Ugandan population[36,53,54]. However, the situation can be determined further by conducting more comprehensive studies on the height profiles of women in Sierra Leone over several decades to determine the changing patterns of women's heights stratified by regions of the country.

Also, one of the insignificant factors of stunting was age-group of 15-19 years which is linked to an age-group where there is rapid growth, increased activities, and high need for adequate nutrients (Table 2). The need for adequate nutrients and diet are paramount for the growth and development of persons in that age category. Our findings that there were no associated factors of stunting with women in specific age-group and poor household wealth indices were inconsistent with literatures from Bangladesh and other countries[55-60].

Adult heights are mainly determined by genetic predilections and environmental factors[57]. In addition to genetic impacts, incomes, social status, infections, and nutrition have been shown to affect body height in European population[60]. Environmental factors are likely to be more important determinants of height in low-and-middle-income countries because environmental stress including food availability and infections are higher in those countries compared to high-income countries[57,58].

Perkins *et al.* explained in their review that short adult stature in low-and-middle-income countries is mainly because of the cumulative net impact of nutrition associated with disease and environmental conditions, such as socio-economic status[56].

The correlates of stunting and underweight among women in the reproductive age (15-49 years) in Sierra Leone were different and has raised our concerns (Table 1, Table 2). Many factors singly or collectively contribute to underweight and stunting including eating patterns, food types, their availability, infections, diseases, physical activity levels, and sleep routines[5,6]. In addition to social determinants of health, genetics, and taking certain medications have been shown to play important roles in undernutrition in a population[5,6,10,61].

If compared with overweight and obesity, the two are mainly caused by food consumption and activities where people gain weight when they eat more calories than they burn through daily activities[61,62]. Also, environmental factors around us matter in the development of obesity and overweight, just like stunting and underweight[62]. The world around us influences our ability to maintain a healthy weight and lifestyle[62]. That has been seen in many African communities where people who are obese are considered healthy, living a prosperous and fulfilling life, an issue which is admired by women in many African communities[62].

On the other side of the spectrum, some communities have begun to admire smaller sizes and equate it to successful and healthy lives. In this, several blue colored individuals have begun to reduce their sizes by conducting regular exercises, eating organic foods, fasting, eating less of fast foods, less snacking, taking less salts and sugars, living less sedentary lifestyle, riding bicycles, or walking to work, sleeping better, avoiding stressful and mental health situations[62].

What is perhaps most interesting from this study is that correlates of underweight and stunting among women in Sierra Leone were different; a factor that should be determined through a comprehensive study, unearthing the underlying reasons. This contrasts with many studies in the African continent[36,41,42,43,45].

Chronic effects of malnutrition in early childhood due to inadequate nutrients and unavailability of food is reflected in later life by stunting and other lifelong consequences such as reduced cognitive function, maternal and child health complications which we did not find in this study population (Table 2).

It is important that these correlates of stunting are addressed in Sierra Leonne's women if improvement in maternal and child health's indicators are to be achieved soon in this country[63]. Feeding habits, diets, and availability

of food for young women in Sierra Leone are prioritized as soon as possible since many young women in the reproductive age are affected by stunting and underweight (Table 1 and Table 2).

In addition, early childhood nutrition programs (for example, school feeding programs) could be a welcome intervention for the school going female children.

It is worth noting that there is limited literature available on stunting among women of reproductive age in Sierra Leone, with most studies focusing on underweight. Therefore, the findings of this study contribute to filling this knowledge gap and could be used for setting proper agenda for the population.

Strengths and limitations of this study: This study has several strengths. First, data quality of this study was assured as the SLDHS-2019 used a well-trained field personnel, standardized protocols, and validated tools in data collection processes. Second, this study utilized a nationally representative sample population of women in the reproductive age of 15-49 years. As a result, findings of the study can be generalizable to the target population in Sierra Leone and many low-to-middle income countries in the African continent. Third, the use of validated tools and calibrated instruments by the SLDHS-2019, the generated estimates are more robust than other studies in Sierra Leone's context. In addition, we used data with a large sample size which was collected, entered, and cleaned by a team of well trained and highly experienced scientists, thus limiting mistakes in the dataset used in the analysis. Finally, as we used concentration index, these findings are more robust in predicting socio-economic inequalities among the study population.

However, this study had limitations which warrant further discussion. First, the SLDHS-2019 was a cross-sectional survey. As a result, we cannot establish a sequential relationship between explanatory and outcome variables. Second, due to the absence of some important data, several significant variables, such as food security and dietary diversity, could not be included in the final model for the analysis. Third, the SLDHS-2019 did not collect individual incomes and expenditures but household data. It used a wealth index as a proxy indicator for household wealth. Fourth, SLDHS collected data only on 15–49 years old women of reproductive age in Sierra Leone. With the current changes in adolescents' reproductive actions and behaviors, there are children less than 15 years who have gone through a full cycle of reproduction. As a result, the distribution of undernutrition among women below and beyond this age group (15-49 years) were not factored in the analysis. Finally, most data on predictors

of undernutrition were based on self-reported information and were not verified through records which risks socially acceptable answers hence social desirability bias in this result.

Generalizability of the results: Results from this study can be generalized to women in reproductive age (15-49 years) in resource settings in low-to-middle-income countries.

Conclusion. The prevalence of underweight and stunting among women of reproductive age (15-49 years) in Sierra Leone were lower compared to regional and world data. Also, the prevalence and correlates of stunting and underweight were different in this study population. These comparisons of anthropometric measurements serve to highlight major differences in correlates and prevalence of stunting and underweight in the same population of women in the reproductive age (15-49 years) in Sierra Leone. However, the two had similar shielding factors in this study population. There is a need to improve the social determinants of health in Sierra Leone in women of reproductive age including school feeding programs among children and adolescents.

In addition, it is important to note that this study findings have important implications for addressing maternal and child health in Sierra Leone. The identified correlates of stunting and underweight should be addressed through targeted interventions. Improving feeding habits, ensuring dietary diversity, and addressing food availability for young women in Sierra Leone should be prioritized. Early childhood nutrition programs, such as school feeding programs, could be effective interventions for improving the nutritional status of school-going female children.

In summary, this study provides valuable insights into the prevalence and correlates of underweight and stunting among women of reproductive age in Sierra Leone. The findings highlight the importance of education, socioeconomic status, and environmental factors in influencing nutritional outcomes. Addressing the correlates of stunting and underweight among women is essential for improving maternal and child health indicators in Sierra Leone. Further research is needed to explore the underlying reasons for the observed differences in correlates between stunting and underweight and to develop targeted interventions to alleviate these nutritional challenges.

Abbreviations

aOR=adjusted Odds Ratio; BMI=Body Mass Index; CI=Confidence Intervals; COR=Crude Odds Ratio; DHS=Demographic Health Survey; EA=Enumeration Areas; SD=Standard Deviation; SLDHS=Sierra Leone Demographic Health Survey; SDGs=Sustainable Development Goals; USAID=United States of America Agency for International Development; WHO=World Health Organization.

Declarations

Ethics approval and consent to participate: This study on women of reproductive age (15-49 years) followed relevant institutional guidelines and regulations and was approved by the Sierra Leone Ethics and Scientific Review Committee (SLESRC), and ICF Institutional Review Board. The study was conducted according to institutional guidelines where written informed consent was obtained from each adult participant and for those under 18 years, assent was obtained in the presence of parents or legal representatives.

Consent for publication: Not applicable

Availability of data and material: All datasets supporting this article's conclusion are within this article and are accessible by a reasonable request to the corresponding author.

Competing interests: All authors declare no conflict of interest.

Funding: Most funds for this study were contributions by individual research members.

Authors' contributions: DLK, JA, and FWDO designed the study. JA, ENI, FWDO, and DLK supervised data management. ENI, JA, MAT, LN, EO, NOA, OE and DLK analyzed and interpreted data. NOA, ENI, FWDO, JA, MAT, EO, LN, KK, AN, OE, JBM, RK, and DLK wrote and revised the manuscript. All the authors approved the revised manuscript.

Acknowledgment: We acknowledge with many thanks for the assistance from 2019 SLDHS for the information obtained.

Authors' Information: Dr. Nelson Onira Alema is a Lecturer in the Department of Anatomy, Faculty of Medicine, Gulu University, Gulu City, Uganda; Dr. Eric Nzirakaindi Ikoona (ENI) is a Technical Director at ICAP at the University of Columbia, Sierra Leone; Dr. Mame Awa Toure (MAT) is at ICAP at the University of Columbia, Freetown, Sierra Leone; Dr. Oliver Eleeza (OE) is an associate director strategic information, ICAP at Columbia University, Sierra Leone; Dr. John Bosco Matovu (JBM) is at ICAP at the University of Columbia, Nairobi, Kenya; Dr. Amon Njenga (AN) is at ICAP at the University of Columbia, Freetown, Sierra Leone; Dr. Lucy Namulemo (LN) is at Foothills Community Based interventions, Monticello, Kentucky, USA; Dr. Ronald Kaluya (RK) is at Uganda Counseling and Support Services, Kampala, Uganda; Dr. Kassim Kamara (KK) is at the Directorate of health security and emergencies, Ministry of Health and Sanitation, Sierra Leone; Dr. Freddy Wathum Drinkwater Oyat (FWDO) is a senior physician, a public health specialist, and a member of Uganda Medical Association, UMA-Acholi branch, Gulu City, Uganda; Dr. Judith Aloyo (JA) is the Deputy Chief of Party at the Rhites-N, Acholi, Gulu City, Uganda; Dr. Emmanuel Olal (EO) is a Physician and a public health specialist at Yotkom Medical Centre, Kitgum, Uganda; Prof. David Lagoro Kitara (DLK) is a Takemi fellow of Harvard University and a Professor at Gulu University, Faculty of Medicine, Department of Surgery, Gulu City, Uganda

References

1. Leslie Elder, Elizabeth Ransom. Nutrition of women and adolescent girls: Why it matters. https://www.prb.org/resources/nutrition-of-women-and-adolescent-girls-why-it-matters/.
2. Sun Movement. Scaling up nutrition: a framework for action. Sun Movement [homepage on the Internet]. 2011. http://scalingupnutrition.org/wp-content/uploads/ pdf/SUN_Framework.pdf.
3. Rotimi C, Okosun I, Johnson L. The distribution of chronic energy deficiency among adult Nigerian men and women. Eur J Clin Nutr.1999;53(9):734-739.
4. World Health Organization. Physical status: the use and interpretation of anthropometry. Geneva: WHO, 1995.
5. Merchant KM, Kurtz KM. Women's nutrition through the life cycle: social and biological vulnerabilities. In: Marge Koblinsky, Judith Timyan and Jill Gay, editors. The health of women: a global perspective. Boulder: Westview Press, 1993;63-90.

6. Bitew FH, Telake DS. Undernutrition among women in Ethiopia: rural-urban disparity. Calverton: ICF Macro, 2010.
7. Stewart CP, Iannotti L, Dewey KG, Michaelsen KF, Onyango AW. Contextualizing complementary feeding in a broader framework for stunting prevention. Matern Child Nutr. 2013;9(suppl 2):27-45.
8. Prado EL, Dewey KG. Nutrition and brain development in early life. Nutr Rev. 2014;72:267-284.
9. Grantham-McGregor S, Cheung YB, Cueto S, Glewwe P, Richter L, Strupp B. Developmental potential in the first 5 years for children in developing countries. Lancet. 2007;369:60-70.
10. Ashraf Soliman, Vincenzo De Sanctis, Nada Alaaraj, Shayma Ahmed, Fawziya Alyafei, Noor Hamed, *et al.,* Early and Long-term Consequences of Nutritional Stunting: From Childhood to Adulthood. Acta Biomed. 2021;92(1): e2021168.
11. Shafique S, Akhter N, Stallkamp G. Trends of under- and overweight among rural and urban poor women indicate the double burden of malnutrition in Bangladesh. Int J Epidemiol. 2007; 36:449–457.
12. Mallia T, Grech A, Hili A. Genetic determinants of low birth weight. Minerva Ginecol. 2017; 69:631–643.
13. Dahlui M, Azahar N, Oche OM. Risk factors for low birth weight in Nigeria: evidence from the 2013 Nigeria Demographic and Health Survey. Glob Health Action 9. 2016;28822.
14. Nnam NM. Improving maternal nutrition for better pregnancy outcomes. Proc Nutr Soc. 2017;74:454-459.
15. Khan MN, Rahman MM, Shariff AA. Maternal undernutrition and excessive body weight and risk of birth and health outcomes. Arch Public Health.2017;75:12.
16. Xu H, Shatenstein B, Luo Z-C. Role of nutrition in the risk of preeclampsia. Nutr Clin Care. 2009; 67:639-657.
17. Chopra M, Galbraith S & Darnton-Hill. A global response to a global problem: the epidemic of overnutrition. Bull World Health Organ. 2002; 80:952–958.
18. Ng M, Fleming T, Robinson M. Global, regional, and national prevalence of overweight and obesity in children and adults 1980–2013: a systematic analysis. Lancet. 2014; 384:766–781.
19. Zahangir MS, Hasan MM, Richardson A. Malnutrition, and non-communicable diseases among Bangladeshi women: an urban–rural comparison. Nutr Diabetes. 2017; 7, e250.
20. Ly KA, Ton TGN, Ngo QV. Double burden: a cross-sectional survey assessing factors associated with underweight and overweight status in Danang, Vietnam. BMC Public Health.2013;13:35.
21. Fenn B, Bulti AT, Nduna T. An evaluation of an operations research project to reduce childhood stunting in a food-insecure area in Ethiopia. Public Health Nutr.2012;15:1746–1754.
22. World Health Organization, UNICEF & US Agency for International Development (2015) Improving Nutrition Outcomes with Better Water, Sanitation and Hygiene: Practical Solutions for Policy and Programmes. Geneva: WHO. 2015.
23. Harris-Fry H, Azad K, Kuddus A. Socioeconomic determinants of household food security and women's dietary diversity in rural Bangladesh: a cross-sectional study. J Health Popul Nutr. 2015;33:12.
24. Islam A, Islam N, Bharati P. Socio-economic and demographic factors influencing nutritional status among early childbearing young mothers in Bangladesh. BMC Women's Health. 2016;16:58.
25. Hossain MG, Bharati P, Aik SAW. Body mass index of married Bangladeshi women: trends and association with socio-demographic factors. J Biosoc Sci. 2012;44:385-399.
26. Bhuiya A & Mostafa G. Levels and differentials in weight, height, and body mass index among mothers in a rural area of Bangladesh. J Biosoc Sci. 1993;25:31-38.
27. Baqui AH, Arifeen SE, Amin S. Levels and correlates of maternal nutrition status in urban Bangladesh. Eur J Clin Nutr. 1994;48:349–357.
28. Ahsan KZ, Arifeen SE, Al-Mamun MA. Effects of individual, household, and community characteristics on child nutritional status in the slums of urban Bangladesh. Arch Public Health.2017;75:9.
29. Statistics Sierra Leone-StatsSL, ICF. Sierra Leone: Demographic and health survey 2019. 2020. https://www.dhsprogram.com/pubs/pdf/FR365/FR365. pdf.
30. World Food Program (WFP). State of Food Security in Sierra Leone 2015 Comprehensive Food Security and Vulnerability Analysis Data collected September - October 2015. 2015 Sierra Leone CFSV.

https://efaidnbmnnnibpcajpcglclefindmkaj/https://documents.wfp.org/stellent/groups/public/documents/ena/wfp288316.pdf?iframe.
31. Worldometer. The population of Sierra Leone. 2022. https://www.worldometers.info/world-population/sierra-leone-population/
32. Sierra Leone NHSSP. National Health Sector Strategic Plan 2017 – 2021. chrome-extension://efaidnbmnnnibpcajpcglclefindmkaj/https://extranet.who.int/countryplanningcycles/sites/default/files/planning_cycle_repository/sierra_leone/sierra_leone_nhssp_2017-21_final_sept2017.pdf
33. World Bank (WB). Microdata in Sierra Leone, 2019. Demographic and Health Survey 2019. Microdata Library. 2019. https://microdata.worldbank.org/index.php/catalog/3826.
34. Statistics Sierra Leone (Stats SL) and ICF. 2020. Sierra Leone Demographic and Health Survey 2019. Freetown, Sierra Leone, Rockville, Maryland, USA: Stats SL and ICF. 2020. chrome-extension://efaidnbmnnnibpcajpcglclefindmkaj/https://dhsprogram.com/pubs/pdf/FR365/FR365.pdf
35. Eric Nzirakaindi Ikoona, Lucy Namulemo, Ronald Kaluya, Freddy Wathum Drinkwater Oyat, Judith Aloyo, David Lagoro Kitara, et al. Prevalence and factors associated with underweight among 15–49-year-old women in Sierra Leone: A Secondary Data Analysis of Sierra Leone Demographic Health Survey of 2019. BMC Women's Health. 2023;23:192.
36. Sserwanja Q, Mukunya D, Habumugisha T, Mutisya LM, Tuke R, Olal E. Factors associated with undernutrition among 20-to 49-year-old women in Uganda: a secondary analysis of the Uganda demographic health survey 2016. BMC Public Health. 2020;20:1644.
37. Zambia Statistics Agency (ZSA), Ministry of Health (MOH). University Teaching Hospital Virology Laboratory – (UTH-VL), ICF. Zambia demographic and health survey. 2018. https://www.dhsprogram.com/pubs/pdf/ FR361/FR361.pdf.
38. Agbadi P, Eunice TT, Akosua AF, Owusu S. Complex samples logistic regression analysis of predictors of the current use of modern contraceptive among married or in-union women in Sierra Leone: insight from the 2013 demographic and health survey. PLoS One. 2020;15:e0231630.
39. Zou D, Lloyd JEV, Baumbusch JL. Using SPSS to analyze complex survey data: a primer. J Mod Appl Stat Methods. 2020;18:2-22.
40. Croft Trevor N, Aileen MJM, Courtney KA. Guide to DHS Statistics. ICF; 2018.
41. Kenya National Bureau of Statistics, Ministry of Health/Kenya, National AIDS Control Council/Kenya, Kenya Medical Research Institute, Population NCf, Development/Kenya: Kenya Demographic and Health Survey 2014. Rockville; 2015.
42. Quraish Sserwanja. Socio-economic determinants of undernutrition among women of reproductive age in Uganda: a secondary analysis of the 2016 Uganda demographic health survey. UPPSALA UNIVERSITET. 2019;1-49. *http://www.diva-portal.org/smash/get/diva2:1367320/FULLTEXT01.pdf*.
43. Ministry of Health CD, Gender, Elderly, Children - MoHCDGEC/Tanzania Mainland, Ministry of Health - MoH/Zanzibar, National Bureau of Statistics - NBS/Tanzania, Office of Chief Government Statistician - OCGS/Zanzibar, ICF: Tanzania Demographic and Health Survey and Malaria Indicator Survey 2015–2016. Dar es Salaam: MoHCDGEC, MoH, NBS, OCGS, and ICF; 2016.
44. Christian P, Smith ER. Adolescent Undernutrition: Global Burden, Physiology, and Nutritional Risks. Annals of Nutrition and Metabolism. 2018;72(4):316-28.
45. Melaku YA, Zello GA, Gill TK, Adams RJ, Shi Z. Prevalence, and factors associated with stunting and thinness among adolescent students in Northern Ethiopia: a comparison to World Health Organization standards. Archives of Public Health. 2015;73(1):44.
46. Reinhardt K, Fanzo J. Addressing Chronic Malnutrition through Multi-Sectoral, Sustainable Approaches: A Review of the Causes and Consequences. Frontiers in nutrition. 2014;1:13.
47. Haseen F. Malnutrition among ultra poor women in Bangladesh: Malnutrition among Bangladeshi Women in Ultra Poor Households: Prevalence and Determinants. Germany: LAP Lambert Academic Publishing, 2010;52-3.
48. Eric Nzirakaindi Ikoona, Lucy Namulemo, Ronald Kaluya, Freddy Wathum Drinkwater Oyat, Judith Aloyo, David Lagoro Kitara. Prevalence and factors associated with underweight among 15–49-year-old women

in Sierra Leone: A secondary Data Analysis of Sierra Leone Demographic Health Survey of 2019. BMC Women's Health. 2023;23:192. https://doi.org/10.1186/s12905-023-02358-4.

49. Negash WD, Fetene SM, Shewarega ES. Multilevel analysis of undernutrition and associated factors among adolescent girls and young women in Ethiopia. BMC Nutr. 2022;8:104. https://doi.org/10.1186/s40795-022-00603-x.
50. Sserwanja Q, Kamara K, Mutisya LM, Musaba MW, Ziaei S. Rural and Urban Correlates of Stunting Among Under-Five Children in Sierra Leone: A 2019 Nationwide Cross-Sectional Survey. Nutr Metab Insights. 2021;14:11786388211047056. doi:10.1177/11786388211047056.
51. Cesar G Victora, Parul Christian, Luis Paulo Vidaletti, Giovanna Gatica-Domínguez, Purnima Menon, and Robert E Black. Revisiting maternal and child undernutrition in low-income and middle-income countries: variable progress towards an unfinished agenda. Lancet.2021;397(10282):1388–1399. doi: 10.1016/S0140-6736(21)00394-9.
52. Watanabe K & Petri WA Jr. Environmental enteropathy: elusive but significant subclinical abnormalities in developing countries. EBioMedicine. 2016;10:25–32.
53. Clark S, Berrang-Ford L, Lwasa S. The burden and determinants of self-reported acute gastrointestinal illness in an Indigenous Batwa Pygmy population in southwestern Uganda. Epidemiol Infect. 2015;143(11):2287–98.
54. Patterson K, Berrang-Ford L, Lwasa S, Namanya DB, Ford J, Twebaze F, *et al*. Seasonal variation of food security among the Batwa of Kanungu, Uganda. Public Health Nutr. 2017;20(1):1–11.
55. Bogin B Scheffler C & Hermanussen M. Global effects of income and income inequality on adult height and sexual dimorphism in height. Am J Hum Biol. 2017; 29.e22980.
56. Perkins JM, Subramanian SV, Davey Smith G. Adult height, nutrition, and population health. Nutr Rev. 2016;74:149-165.
57. Silventoinen K. Determinants of variation in adult body height. J Biosoc Sci. 2003;35:263–285.
58. de Oliveira VH & Quintana-Domeque C. Early-life environment and adult stature in Brazil: an analysis for cohorts born between 1950 and 1980. Econ Hum Biol. 2014; 15:67–80.
59. National Institute of Population Research and Training, Mitra, and Associates, & ICF International (2014) Bangladesh Demographic and Health Survey 2014. Dhaka and Rockville, MD: NIPORT, Mitra and Associates, and ICF International.
60. Fudvoye J & Parent AS. Secular trends in growth. Ann Endocrinol (Paris). 2017;78:88-91.
61. Ken Maleta. Undernutrition. Malawi Medical Journal; 2006;18(4):189-205.
62. Eric Nzirakaindi Ikoona, Mame Awa Toure, Kassim Kamara, Freddy Wathum Drinkwater Oyat, Judith Aloyo, David Lagoro Kitara, *et al*. Double Burden of Malnutrition Among Women in Reproductive Age (15-49 years) in Sierra Leone: A Secondary Data Analysis of the Demographic Health Survey of 2019 (SLDH-2019). Research Square. 2022. DOI: 10.21203/rs.3.rs-1992723/v1.
63. Survey Quraish Sserwanja, Kassim Kamara, Linet M Mutisya, Milton W Musaba and Shirin Ziaei. Rural and Urban Correlates of Stunting Among Under Five Children in Sierra Leone: A 2019 Nationwide Cross-Sectional. Nutrition and Metabolic Insights. 2021;4:1–10.
