# Supplementary material for: Prevalence and factors associated with undernutrition among 15–49-year-old women in Sierra Leone: A secondary data analysis of Sierra Leone Demographic Health Survey of 2019
Source: PLoS One. 2024 Nov 11;19(11):e0311845. doi: 10.1371/journal.pone.0311845 (PMC11554195; doi:10.1371/journal.pone.0311845)
Supplement: Attachment — Submitted filename: Comments.docx [file pone.0311845.s002.docx]

Comments for the project entitled “Prevalence and factors associated with undernutrition among 15–49-year-old women in Sierra Leone: A secondary data analysis of Sierra Leone Demographic Health Survey of 2019”:

1. The whole study based on only four data sets, BMI of reproductive age group females, Wealth proxy index and educational status and parity. Author associated underweight with level of education and wealth index. However, Stunting is a complex condition and dependent upon many factors like, genetic factor, hook warm infection, anemia, vitamin D deficiency, Diet and protein intake, diarrheal diseases, vaccination, age of marriage etc. The study is lacking of data of nutritional status, infection history, psycho-social development status etc. Thus in absence of all such data, interpretation of ‘ lower prevalence of underweight and stunning among reproductive age women in sierra Leone’ may not be correct and needs stronger support for such conclusion.
2. The Author mentioned (in result) that 7514 women of reproductive age group participated in the study- which give a wrong impression that it is an active surveillance , whereas it is a secondary data analysis and data of 7514 participants taken from 2019 demographic health survey.
3. Out of 15,934 women, selection of 7514 is not properly justified, author mentioned that those responded and within reproductive age group and have data of BMI etc were selected. But did not provided a flow chart for selection. Thus selection bias cannot be ruled out. Rather a detailed flow chart of total participant of the survey ( n=XX), eligible women (mentioning eligibility criteria) ( n=xx), not eligible( n=xxx) and why, total sample available for the study( n=XX) etc. may help to understand the selection process.
4. As per the WHO definition of stunning is “height –for-Age of children below 5 years is more than two standard deviations below the WHO’s child growth standards median”. Although author mentioned WHO criteria, but it seems not followed properly as they calculated 113/7514 ( 15-49 years age) as stunning based on weight and height only.
5. Control sample is missing. The male of same age group would help to understand the claim of undernutrition and stunning. Also analysis of 0-5 years of age group data may help to support the conclusion.
6. The conclusion of the study could not be supported by required data set.
