# Supplementary material for: Prevalence and factors associated with undernutrition among 15–49-year-old women in Sierra Leone: A secondary data analysis of Sierra Leone Demographic Health Survey of 2019
Source: PLoS One. 2024 Nov 11;19(11):e0311845. doi: 10.1371/journal.pone.0311845 (PMC11554195; doi:10.1371/journal.pone.0311845)
Supplement: Attachment — Submitted filename: PONE-D-23-29763.docx [file pone.0311845.s003.docx]

**Prevalence and factors associated with undernutrition among 15–49-year-old women in Sierra Leone: A secondary data analysis of Sierra Leone Demographic Health Survey of 2019.**

**Nelson Onira Alema^1^**^¶^**, Eric Nzirakaindi Ikoona^2&^, Mame Awa Toure^2^**^¶^**, Oliver Eleeza^2^**^¶^**, Amon Njenga^2^**^¶^**, John Bosco Matovu^3^**^¶^**, Lucy Namulemo^4,5,6^**^¶^**, Ronald Kaluya^6^**^¶^**, Kassim Kamara^7^**^¶^**, Freddy Wathum Drinkwater Oyat^8&^, Emmanuel Olal^8,9&^, Judith Aloyo^8,10&^, David Lagoro Kitara^8,11,12*^.**

^1^Department of Anatomy, Faculty of Medicine, Gulu University, Gulu City, Uganda.

^2^ICAP at Columbia University, Freetown, Sierra Leone.

^3^ICAP at Columbia University, Nairobi, Kenya.

^4^Foothills Community Based interventions, Monticello, Kentucky, USA.

^5^Lindsey Wilson College, School of Professional Counseling, Kentucky, USA.

^6^Uganda Counseling and Support Services, Kampala, Uganda.

^7^Directorate of Health Security and Emergencies, Ministry of Health and Sanitation, Sierra Leone.

^8^Uganda Medical Association (UMA), UMA-Acholi branch, Gulu City, Uganda.

^9^Yotkom Medical Centre, Kitgum, Uganda.

^10^Rhites-N, Acholi, Gulu City, Uganda.

^11#b^Gulu Centre for Advanced Medical Diagnostics, Research, Trainings, and Innovations (GRUDI BIONTECH INITIATIVE), Gulu City, Uganda.

^*12#a^Gulu University, Faculty of Medicine, Department of Surgery, Gulu City, Uganda.

#aCurrent Address: Gulu University, Faculty of Medicine, Department of Surgery, Gulu City, Uganda.

#bCurrent Address: Gulu Centre for Advanced Medical Diagnostics, Research, Trainings, and Innovations (GRUDI BIONTECH INITIATIVE), Gulu City, Uganda.

****Corresponding Author:*** *David Lagoro Kitara is a Takemi fellow of Harvard University and Faculty at Gulu University, Faculty of Medicine, Department of Surgery, P.0. Box 166, Gulu City, Uganda, email:* [*klagoro2@gmail.com*](mailto:klagoro2@gmail.com)*; phone: (+256)772524474; ORCID: 0000-0001-7282-5026.*

**Abstract**

**Background:** Undernutrition of women of childbearing age is pertinent for maternal and offspring health. This study aimed to determine the prevalence and associated factors of undernutrition (underweight and stunting) among women of reproductive age (15-49 years) in Sierra Leone using secondary data analysis of the 2019 Demographic Health Survey.

**Methods:** Anthropometric measurements and maternal characteristics were obtained from the Sierra Leone Demographic Health Survey (SLDHS) of 2019. The heights and weights of women were measured, and BMI in kg/m^2^ was calculated. Based on the World Health Organization's recommendations, stunting was defined as heights <145cm and being underweight as BMI <18.5kg/m^2^. Multivariate logistic regression analyses were conducted to identify correlates of undernutrition, with a significance level set at p<0.05.

**Results**: A total of 7,514 women of reproductive age, 15-49 years were analyzed in this study. The prevalence of stunting and underweight was 1.5% (113/7514) and 6.7%(502/7,514), respectively. Women with primary education had a 47% lower likelihood of stunting (adjusted Odds Ratio [aOR]=0.53, 95% Confidence Interval [CI]:0.30-0.94;p=0.029) than those with secondary education. Women in the poorest wealth index had a 51% lower likelihood of being stunted (aOR=0.49,95%CI:0.27-0.88;p=0.017) than middle wealth index. Underweight was 1.48 times more likely among women with parity of one-to-four (aOR=1.48,95% CI:1.08-2.03;p=0.015) than women who never gave birth. In addition, underweight was 1.41 times more likely among women who listened to radios (aOR=1.41,95% CI:1.14-1.74;p=0.002) than those who did not. Age groups of 15-19 years and 40-49 years had a 54% (aOR=0.46,95%CI:0.34-0.62;p<0.001) and 34% (aOR=0.66,95%CI:0.45-0.97;p=0.035) lower likelihood of being underweight than 20-29-year age group, respectively. Women with primary education had a 26% lower likelihood of being underweight (aOR=0.74,95%CI:0.56-0.99;p=0.042) than those with secondary education. However, none of the wealth indices was significantly associated with underweight.

**Conclusion:** The prevalence of underweight and stunting among women of reproductive age (15-49 years) in Sierra Leone was lower than regional and world data. This study highlights similarities and differences in this population's prevalence and correlates of undernutrition. Underweight and stunting were less likely in women with primary education, while parity of one-to-four and listening to radios were significantly associated with being underweight. Further trend studies using DHS data from 2010, 2014, and 2019 are warranted to understand the dynamics of undernutrition in Sierra Leone.

**Keywords:** Underweight, stunting, women of reproductive age (15-49 years), Sierra Leone, undernutrition.

**Introduction:** Malnutrition, characterized by deficiencies in calories, protein, vitamins, minerals, poor health, and social conditions, poses a significant health challenge for millions of women and adolescent girls worldwide [1]. Adequate nutrition is crucial for women's overall health and has far-reaching implications for the well-being of their children [1]. Children born to malnourished women are at higher risk of cognitive impairments, stunted growth, increased susceptibility to infections, and elevated morbidity and mortality rates throughout their lives [1].

Undernutrition remains a pressing global health issue, encompassing being underweight, wasted, stunted, and with deficiencies in essential minerals and vitamins [2]. Research indicates that women with a body mass index (BMI) below 18.5kg/m^2^ in developing countries face an escalating mortality risk and heightened vulnerability to illnesses [2-4]. Accordingly, the impact of malnutrition extends beyond women's health, affecting the well-being of their children [5-6]. This scenario perpetuates a cycle of undernutrition that spans generations [5-6], especially in countries like Sierra Leone, where social and biological factors such as civil unrest, poverty, epidemic outbreaks, and food insecurity contribute to women's vulnerability to undernutrition [5]. In addition, numerous individual, household, and community factors influence women's nutritional health status [5-6].

On the one side, stunting is a consequence of complex interactions among household, environmental, socioeconomic, and cultural factors [7-9]. It has detrimental effects such as susceptibility to infections, impaired cognitive and motor development, and elevated risks of non-communicable diseases later in life [7-9]. Also, research has shown that individuals who experience stunting during childhood were more likely to face challenges such as poor cognitive function, lower educational performance, reduced adult wages, decreased productivity, and an increased risk of nutrition-related chronic diseases in adulthood [10]. Therefore, ensuring adequate nutrition is a fundamental foundation for individual and population health [11-16].

Furthermore, maternal undernutrition, underweight, and stunting have been linked to adverse maternal health conditions, such as chronic energy deficiency, cesarean delivery, pre-eclampsia, anemia, decreased productivity, mental health issues, and adverse pregnancy outcomes [11-16]. On the other end of the malnutrition spectrum, overweight and obesity pose significant health risks for women, including a higher likelihood of developing hypertension, diabetes, cardiovascular diseases, and stroke [17–20].

The determinants of undernutrition in women encompass many factors, including community-level water, sanitation, and hygiene (WASH) practices [21,22], food stability status [23], household income and wealth, women's educational level, age at first marriage, age at first delivery, multiparity, short birth intervals, and land ownership [19,24-28]. Therefore, identifying maternal nutritional prevalence levels and determinants is crucial for targeted interventions and resource allocation in resource-limited settings [19,21-28].

Despite the significance of understanding maternal nutritional status, limited research has been conducted in Sierra Leone, often focusing solely on malnutrition determinants in young children and adolescents. The present study addresses this research gap by investigating the risk factors of undernutrition among women of reproductive age (15-49 years) in Sierra Leone, utilizing data from the Sierra Leone Demographic Health Survey (SLDHS-2019).

Findings of this study hold essential policy implications from a global health perspective and specifically for Sierra Leone, aiding in monitoring progress toward sustainable development goals (SDGs) and regional nutrition strategies. Moreover, the study can guide the allocation of limited resources by the Government and health stakeholders to improve the nutritional and health status of women and infants in Sierra Leone.

In using data from a population-based cohort of women of childbearing age in Sierra Leone, this study aimed to determine the prevalence and associated factors of undernutrition (underweight and stunting) among women of reproductive age (15-49 years) based on the 2019 Demographic Health Survey.

**Methods.**

**Study design:** The SLDHS-2019 was conducted as a countrywide representative cross-sectional survey led by the Bureau of Statistics of Sierra Leone (Stats SL) with technical assistance from ICF through DHS programs. This survey was funded by the United States Agency for International Development (USAID) [29].

**Study sites:** This study was conducted in all four provinces and western areas of Sierra Leone [29].

**Sampling and study population:** The sampling of the study respondents was based on the 2015 population and housing census of the Republic of Sierra Leone [30]. This census was conducted by Statistics Sierra Leone (Stats SL) and the 2015 population and housing census provided the ready-made sampling frame for the SLDHS-2019 [30]. Sierra Leone is administratively divided into four provinces and western areas (urban and rural), sixteen districts, and 190 chiefdoms [30,31,32]. Each district is subdivided into chiefdoms/census wards, and each chiefdom/census ward is subdivided into sections [30,31,32]. In addition, the 2015 population and housing census subdivided each locality into convenient census; the enumeration areas (EAs) [30,33]. The EAs were the primary sampling units (PSUs) and clusters for the SLDHS-2019 [30-35]. The list of EAs from the 2015 census formed the basis for estimating the number of households required and classifying EAs (clusters) into urban/rural for the SLDHS-2019 sampling frame [30,31,34,35].

Furthermore, the SLDHS-2019 employed a two-stage stratified sampling design, and stratification was achieved by classifying each district into urban and rural areas [34,35]. So, thirty-one sampling strata were created, and samples were selected independently in each stratum via a two-stage selection process [34,35].

Thus, implicit stratifications were achieved at each lower administrative level by sorting the sampling frame before sample selection according to administrative order and using a probability proportional-to-size selection during the first sampling stage [34,35].

Also, five hundred and seventy-eight (578) EAs were selected using a probability proportional to EA size [34,35] in the first stage of the selection process. In addition, the enumeration area size was determined by the number of households residing in it, and a household listing operation was performed in all selected enumeration areas [34,35]. The resulting lists of households served as a sampling frame for selecting households in the second stage of the survey [34,35].

In the second stage's selection, a fixed number of twenty-four households was chosen in every cluster through an equal probability systematic sampling, resulting in a total sample size of approximately 13,872 households distributed in 578 clusters [34,35]. The household listing in this stage was conducted using computer tablets, and households were randomly selected through computer programming [34,35].

The survey interviewed only pre-selected households in the clusters, and no replacements or changes of the selected households were allowed in the implementation stage of the survey to prevent selection bias of the study population [34,35]. Due to the non-proportional allocation of samples to the sixteen districts in Sierra Leone and the possible differences in response rates, sample weights were calculated, added to the data file, and applied so that the results would be representative at national and domain levels [34,35]. Further, because the SLDHS-2019 sample was a two-stage stratified cluster sampling, sample weights were calculated separately at each sampling stage based on sampling probabilities [34,35]. After that, the SLDHS-2019 included all women aged 15-49 in the sampled households [34,35]. Permanent residents in the selected homes and visitors who stayed overnight before the survey were eligible for interviews in the household [34,35]. The Man's questionnaire covered the identification of respondents, background information, reproduction, contraception, marriage and sexual activity, fertility preferences, employment status, gender roles, HIV and AIDS, and other health-related issues [35]. The Biomarker questionnaire covered the identification of respondents, weights, heights, and hemoglobin measurements for children aged 0–5 years, weights, heights, HIV testing, and hemoglobin measurements for women aged 15–49 years [35]. The Fieldworker questionnaire covered the background information on each field worker [35].

**Anthropometric measurements.** The weight of respondents was recorded in kilograms (kg) to the nearest decimal point and was measured using an electronic scale (SECA 878) [34,35]. Participants' heights were measured using a stadiometer in centimeters (cm) to one decimal point [34,35]. The Body Mass Index (BMI) of respondents was calculated in kg/m^2^ using weights (in kilograms) and heights (meters) of women of reproductive age (15–49 years) and classified according to WHO criteria as underweight (<18.5kg/m^2^), normal weight (18.5–24.9kg/m^2^), overweight (25.0–29.9kg/m^2^), obesity (≥30.0kg/m^2^ and ≤50.0kg/m^2^), and overnutrition (≥25.0kg/m^2^ and ≤50.0kg/m^2^).

**Wealth Index (WI).** To calculate each household's wealth, we used wealth index (WI) as a proxy indicator of household wealth [35]. This composite index used household key asset ownership variables to calculate each household wealth index from the SLDHS-2019 data [35]. These variables were the characteristics of the household's dwelling unit, for example, the source of water, type of toilet facilities, type of fuel used for cooking, number of rooms, ownership of livestock, possessions of durable goods, mosquito nets, and primary materials for the floor, roof, and walls of the dwelling place [35]. The respondent's household wealth index was calculated using computer analysis of household composite factors [35]. It was then categorized into five quintiles: poorest, poorer, middle, richer, and richest wealth indices. (Table 1)

**Operational definitions.**

**Body Mass Index (BMI):** Weight in kilograms divided by heights in meters squared (kg/m^2^).

**Underweight:** BMI <18.5kg/m^2^

**Overweight:** BMI ≥25.0kg/m^2^ and ≤29.9kg/m^2^

**Obese:** BMI ≥30.0kg/m^2^ and ≤50.0kg/m^2^

**Overnutrition (Overweight and obese):** BMI ≥25.0kg/m^2^ and ≤50.0kg/m^2^.

**Enumeration Area (Clusters):** An EA is a geographic area consisting of a convenient number of dwelling units that serve as a counting unit for the survey.

**Data Collection:** Data collection for this survey was conducted from May 14, 2019, to August 31, 2019 [29]. The primary sampling unit (PSU), a cluster, was based on enumeration areas (EAs) obtained from the 2015 EA population census sampling frame [29]. The SLDHS-2019 used five validated questionnaires for the thematic parts of the survey [29]. The Household questionnaire collected data on household environment, assets, and basic demographic information of household members. The Woman's questionnaire collected data on women's reproductive health, domestic violence, and nutrition indicators [29]. The Man's questionnaire collected data on men's health, while the Biomarker questionnaire collected data on anthropometry and blood tests for mothers and children (0-5 years), and the Fieldworker questionnaire collected data on background information of fieldworkers [36,37].

This secondary data analysis included women of reproductive age, 15-49 years, whose anthropometric characteristics were recorded with consent. Trained health technicians were deployed to measure the heights and weights of the participants to ensure the quality of anthropometric measurements [29].

Out of the weighted sample of 15,574 women in the dataset, 7,514 anthropometric measurements were included in the survey design, while 8,060 had invalid weight and height measurements due to erroneous and ineligible measurements. The weight and height measurements are vital for calculating the BMI of each participant, which was finally used for assessing the nutritional status of each respondent.

Number of women in the Survey (N=15,574)

Number of women with no height and weight measurements (52%) (n=8,060)

Number of Women with BMI Measurements (48%) (n=7,514)

**Figure 1: Flow chart for the study.**

In some respondents' results, heights and weights were not well recorded (n=7548) and some respondents refused to have weights and heights taken (n=512), and we could only obtain completed anthropometric measurements for 7,514 women who were not lactating, non-pregnant, and post-menopausal women. In the final analysis, a weighted sample 7,514 was included in our secondary data analysis, as summarized in Figure 1 and Table 1. A complete protocol with detailed explanations about data collection processes and sampling is available online [29].

**Outcome variables:** The first outcome variable for this study was stunting among women (15-49 years). It was defined as heights of <145cm ± Standard Deviations (SD) from the median value set by the World Health Organization (WHO). The second outcome variable was underweight (BMI <18.5kg/m^2^).

**Independent variables:** The independent variables in this study were based on previous studies, the WHO stunting framework, underweight, normal weight, and available information in the SLDHS-2019 database. We included sixteen independent variables (age, parity, type of residence, sex of household head, Household size, work-status, marital status, regions of Sierra Leone, level of education, wealth index, BMI categories, listening to radios, reading of magazines, watching television, smoking cigarettes, and alcohol use) in this data analysis.

**Women's characteristics:** Parity (categorized as para 0, para one-to-four, and five and above), work status (categorized as working-class versus not working), marital status (categorized as married versus not married/single), levels of education (categorized as no education, primary, secondary, and higher), age groups (categorized as 15-19, 20-29, 30-39, and 40-49 years), woman's stunting status (defined as heights <145cm for stunted and ≥145cm for not stunted women), and woman's BMI classification as normal BMI (18.5-24.9kg/m^2^) and underweight (<18.5kg/m^2^).

**Household characteristics:** These characteristics include; regions of Sierra Leone (Northwest, Eastern, Western, Southern, and Northern); household wealth indices (categorized as richest, richer, middle, poorer, and poorest); sex of the head of household (female versus male); household size (less than six versus six and above); residency (urban versus rural); watching television (yes versus no); reading of magazines (yes versus no); listening to radios (yes versus no); smoking cigarettes (yes versus no); and alcohol use (yes versus no).

**Ethical approval:** This survey protocol was approved by the Sierra Leone Ethics and Scientific Review Committee (SLESRC) and the ICF Institutional Review Board. In addition, this study was conducted according to institutional guidelines, where written informed consent was obtained from each adult respondent. For those under 18 years, assent was obtained in the presence of parents or legal representatives.

**Data analysis:** Frequency tables and proportions/percentages were used to describe summaries of categorical variables, while means and standard deviations (±SD) were used for continuous variables. Sample weights were used to account for unequal probability sampling in different study population strata and ensured the representativeness of the survey results at all levels [29]. Statistical software SPSS version 25.0 Statistical software complex samples package incorporating all variables in the analysis plan was used to account for the multistage sampling design inherent in the DHS dataset, including individual sample weight, sample strata for sampling errors/design, and cluster numbers [38-40].

Using a complex sample package ensured the sampling design was incorporated into the analysis, leading to accurate and reliable results. Cross tabulations were conducted, and associations between socio-demographic and economic characteristics, and women's nutritional status (stunting and underweight), including their Odds ratios (OR) and P-values, were presented.

To assess associations of each independent variable with dependent variables (stunting and underweight), a bivariate logistic regression analysis was conducted, and Crude Odds Ratios (COR), at 95% Confidence Intervals (CI) and P-values were presented. Independent variables which were found significant at bivariate level, and those with P-values ≤0.20 were included in the final multivariate logistic regression analysis model for each dependent variable. The final regression model excluded variables with P-values above 0.201 at the bivariate level and the adjusted Odds Ratios (aOR), at 95% Confidence Intervals (CI), and corresponding P-values were calculated, with statistical significance levels set at 0.05.

**Sensitivity analysis:** Sensitivity analysis for stunting was conducted by excluding women with parity of five and above in the multivariate logistic regression model, as it had only 23(1.3%) stunted women. By excluding them from the final regression model, the other factors remained significant, and no substantial changes were observed in the strength of associations. The same statistical approach was used for studying underweight in this study population. Cross tabulations were conducted, and associations between socio-demographic characteristics and women's nutritional status (underweight versus normal weight), including their aOR at 95% CI and P-values, were presented with no significant differences observed after excluding women with BMI ≤15.0kg/m^2^.

**Results and discussions.**

The study was a secondary data analysis of the demographic health survey conducted in Sierra Leone in 2019 and included 7,514 women of reproductive age, 15-49 years (Table 1, Figure 1). Among the women, the majority belonged to the 20-29-year age group, accounting for 33.6%(2528/7514) of the total population. Women with parity ranging from one to four represented just over 50%(51.8%; 3892/7514) of the total study population, while most participants resided in rural areas, 58.9%(4,422/7,514) of Sierra Leone. Male-headed households constituted slightly over two-thirds of the study population at 71.3%(5,356/7,514). Moreover, households with a size of six or more individuals constituted the majority at 60.1%(4,519/7,514) (Table 1).

Most study population were working women, representing 69.7%(5,234/7,514) of the population. Among marital status categories, married women constituted 63.8%(4,795/7,514). Regionally, women from the south of Sierra Leone constituted the largest proportion at 24.4%(1,831/7,514), followed by the north at 24.2%(1,822/7,514), the east at 21.0%(1,579/7,514), the west at 16.7%(1,256/7,514), and the northwest at 13.7%(1,026/7,514). In educational level, the majority had no formal education, accounting for 47.5%(3,571/7,514), followed by secondary education at 35.2%(2,641/7,514), primary education at 13.5%(1,017/7,514), and a smaller proportion with higher education at 3.8%(285/7,514) (Table 1).

Regarding wealth indices, 21.7%(1,634/7,514) of women were in the richer wealth index category, followed by the poorest and middle at 20.4%(1,533/7,514) each, poorer wealth index at 19.0%(1,482/7,514), and the smallest proportion among the richest wealth index at 18.5%(1,388/7,514) (Table 1).

In terms of BMI categories, most women had normal BMI, accounting for 66.2%(4,974/7,514), followed by overweight at 19.7%(1,479/7,514), obese at 7.4%(5,59/7,514) and the smallest proportion among underweight women at 6.7%(502/7,514) (Table 1).

Regarding social activities, most participants did not watch television, accounting for 74.9%(5,625/7,514). Furthermore, most participants did not listen to radios 58.2%(4,372/7,514) and did not read magazines 93.5%(7,025/7,514). Additionally, most women did not smoke cigarettes 97.0%(7,290/7,514) and did not use alcohol 41.0%(3,081/7,514) (Table 1).

**The prevalence of stunting:**  Out of 15,574 women in the SLDHS-2019, 48%(7,514/15,574) had valid height measurements. The mean height was 157.6cm with a standard deviation (SD) of ±6.3cm. The minimum recorded height was 107.7cm, and the maximum was 186.2cm (Figure 2). The overall prevalence of stunting in the study population was 1.5%(113/15,574) (Table 2).

**The data source is SLDHS of 2019.**

**Figure 2: The heights of women in the reproductive age (15-49 years) in Sierra Leone.**

Figure 2 shows normally distributed heights among women aged (15-49) years in Sierra Leone. The mean height was 157.6 cm, SD±6.3.

**The prevalence of underweight:** Among the total population of women (n=7,514), the mean BMI was 23.8kg/m^2^ (SD±4.7). The prevalence of underweight was 6.7%(502/7,514), with a minimum BMI recorded at 12.8kg/m^2^. Within the underweight category, two outlier BMI values were 12.8kg/m^2^ and 14.5kg/m^2^, each representing 0.03% of the total study population. These outlier BMIs were situated on the left side of the normal distribution of underweight (Table 2).

Frequency

| Age-groups of participants (years) | underweight | Stunted |
| --- | --- | --- |
| 15-19 | 227 | 35 |
| 20-29 | 106 | 33 |
| 30-39 | 97 | 32 |
| 40-49 | 72 | 13 |

**The data source is SLDHS of 2019.**

**Figure 3: Frequency of underweight among age-groups of women (15-49 years) in the 2019 SLDHS**

Figure 3 shows the frequency of underweight as it decreased with age group populations, with the majority in the 15-19-year age group 45.2%(227/502), followed by the 20-19-year age group, 21.1%(106/502); 30-39-year age group 19.3%(97/502), and least among the 40-49-year age group 14.3%(72/502).

**Correlates of stunting among women of reproductive age (15-49 years) in Sierra Leone:** The study showed that primary education and being in the poorest wealth index were less likely factors of being stunted among the study population. Women with a primary education had a 47% lower likelihood of being stunted (aOR=0.53,95%CI:0.30-0.94;p=0.029) than those with secondary education. Similarly, women in the poorest wealth index had a 51% lower likelihood of being stunted (aOR=0.49,95%CI:0.27-0.88;p=0.017) than those in the middle wealth index. Other factors such as parity, residence (urban or rural), sex of the household head, household size, work status, marital status, regions of residence, listening to radios, reading of magazines, watching television, alcohol use, and smoking cigarettes did not significantly affect the occurrence of being stunted among study participants (Table 2).

**Correlates of underweight among women (15-49 years) in Sierra Leone.** After adjusting for individual characteristics in the final multivariate logistic regression model, the determinants of being underweight among Sierra Leonean women of reproductive age (15-49 years) were identified: Women with parity of one-to-four had a 1.48 times more likelihood of being underweight (aOR=1.48, 95%CI:1.08-2.03; p=0.015) than women who never gave birth. In addition, women who listened to radios were 1.41 times more likely of being underweight (aOR=1.41,95%CI:1.14-1.74; p=0.002) than those who did not. However, being in the age group of 15-19 years was associated with a 54% lower likelihood of being underweight (aOR=0.46, 95% CI:0.34-0.62; p<0.001) than the 20-29-year age-group and being in the age group of 40-49 years was associated with a 34% lower likelihood of being underweight (aOR=0.66, 95%CI:0.45-0.97; p=0.035). Furthermore, having primary education was associated with a 26% lower likelihood of being underweight (aOR=0.74, 95% CI: 0.56-0.99; p=0.042) than secondary education. None of the wealth indices showed a significant association with being underweight in this study population (Table 3).

**Table 1: Socio-economic and demographic characteristics of women in the reproductive age (15-49 years) in Sierra Leone.**

| **Variables** | **Frequency (n=7,514)** | **Percent (%)** |
| --- | --- | --- |
| **Ages (years)** |  |  |
| 15-19 | 1,616 | 21.5 |
| 20-29 | 2,528 | 33.6 |
| 30-39 | 2,048 | 27.3 |
| 40-49 | 1,322 | 17.6 |
| **Parity** |  |  |
| Never gave birth | 1,895 | 25.2 |
| One to four | 3,892 | 51.8 |
| Five and above | 1,727 | 23.0 |
| **Type of residence** |  |  |
| Urban | 3,092 | 41.1 |
| Rural | 4,422 | 58.9 |
| **Sex of the head of household** |  |  |
| Male | 5,356 | 71.3 |
| Female | 2,158 | 28.7 |
| **Household size** |  |  |
| Less than six | 2,995 | 39.9 |
| Six and above | 4,519 | 60.1 |
| **Work status** |  |  |
| Not working | 2,280 | 30.3 |
| Working | 5,234 | 69.7 |
| **Marital status** |  |  |
| Married | 4,795 | 63.8 |
| Not married | 2,719 | 36.2 |
| **Regions of Sierra Leone** |  |  |
| East | 1,579 | 21.0 |
| North | 1,822 | 24.2 |
| Northwest | 1,026 | 13.7 |
| South | 1,831 | 24.4 |
| Western | 1,256 | 16.7 |
| **Levels of education** |  |  |
| No formal education | 3,571 | 47.5 |
| Primary | 1,017 | 13.5 |
| Secondary | 2,641 | 35.2 |
| Higher | 285 | 3.8 |
| **Wealth Indices** |  |  |
| Poorest | 1,533 | 20.4 |
| Poorer | 1,428 | 19.0 |
| Middle | 1,531 | 20.4 |
| Richer | 1,634 | 21.7 |
| Richest | 1,388 | 18.5 |
| **BMI categories (kg/m^2^)** |  |  |
| Underweight (<18.5) | 502 | 6.7 |
| Normal weight (18.5-24.9) | 4,974 | 66.2 |
| Overweight (25.0-29.9) | 1,479 | 19.7 |
| Obese (≥30.0) | 559 | 7.4 |
| **Watching Television** |  |  |
| Yes | 1,889 | 25.1 |
| No | 5,625 | 74.9 |
| **Listening to radios** |  |  |
| Yes | 3,142 | 41.8 |
| No | 4,372 | 58.2 |
| **Reading of magazines** |  |  |
| Yes | 489 | 6.5 |
| No | 7.025 | 93.5 |
| **Smoking of cigarettes** |  |  |
| Yes | 224 | 3.0 |
| No | 7,290 | 97.0 |
| **Alcohol use** |  |  |
| Yes | 667 | 8.9 |
| No | 3,081 | 41.0 |

**The data source is SLDHS-2019.**

In Table 1 the majority of women of reproductive age (15-49 years) in Sierra Leone were in the 20-29-year age group 2528/7514(33.6%); parity of one-to-four 3892/7514(51.8%); of rural residence 4422/7514(58.9%); male-headed households 5356/7514(71.3%); household size of six and above 4519/7514(60.1%); working class 5234/7514(69.7%); married 4,795/7514(63.8%); from the South 1831/7514(24.4%); had no formal education 3571/7514(47.5%); richer wealth index 1634/7514(21.7%); normal weight 4974/7514(66.2%); did not watch television 5625/7514(74.9%); did not listen to radios 4372/7514(58.2%); did not read magazines 7025/7514(93.5%), did not smoke cigarettes 7290/7514(97.0%), and did not use alcohol 3081/7514(41.0%).

**Table 2: Bi- and multivariate analysis of stunting among women (15-49 years) in SLDHS-2019.**

| **Variables** | **Stunted**  **(n=113)**  **(n, %)** | **Not stunted**  **(n=7,401)**  **(n, %)** | **Unadjusted**  **POR** | **95% CI** | **p value** | **aOR** | **95% CI** | **p value** |
| --- | --- | --- | --- | --- | --- | --- | --- | --- |
|  |  |  |  |  |  |  |  |  |
| **Age groups (years)** |  |  |  |  |  |  |  |  |
| 20-29 | 33(1.3) | 2495(98.7) | **Reference** |  |  | **Reference** |  |  |
| 15-19 | 35(2.2) | 1582(97.8) | 0.597 | 0.370-0.965 | 0.035 | 0.815 | 0.437-1.520 | 0.419 |
| 30-39 | 32(1.6) | 2016(98.4) | 0.833 | 0.511-1.360 | 0.465 | 0.936 | 0.533-1.644 | 0.520 |
| 40-49 | 13(1.0) | 1309(99.0) | 1.332 | 0.699-2.539 | 0.384 | 1.559 | 0.741-3.277 | 0.818 |
| **Parity** |  |  |  |  |  |  |  |  |
| Never gave birth | 38(2.0) | 1857(98.0) | **Reference** |  |  | **Reference** |  |  |
| One to four | 52(1.3) | (3840(98.7) | 1.511 | 0.991-2.304 | 0.055 | 1.489 | 0.792-2.801 | 0.216 |
| Five and above | 23(1.3) | 1704(98.7) | 1.516 | 0.900-2.555 | 0.118 | 1.524 | 0.659-3.524 | 0.324 |
| **Residence** |  |  |  |  |  |  |  |  |
| Rural | 80(1.8) | 4342(98.2) | **Reference** |  |  | **Reference** |  |  |
| Urban | 33(1.1) | 3059(98.9) | 1.708 | 1.136-2.569 | 0.010 | 1.257 | 0.614-2.572 | 0.531 |
| **Sex of the household head** |  |  |  |  |  |  |  |  |
| Male | 76(1.4) | 5280(98.6) | **Reference** |  |  |  |  |  |
| Female | 37(1.7) | 2121(98.3) | 0.825 | 0.555-1.226 | 0.342 |  |  |  |
| **Household size** |  |  |  |  |  |  |  |  |
| Six and above | 59(1.3) | 4460(98.7) | **Reference** |  |  | **Reference** |  |  |
| Less than six | 54(1.8) | 2941(98.2) | 0.72 | 0.497-1.045 | 0.084 | 0.761 | 0.518-1.112 | 0.166 |
| **Work status** |  |  |  |  |  |  |  |  |
| Not working | 33(1.4) | 2247(98.6) | **Reference** |  |  |  |  |  |
| Works | 80(1.5) | 5154(98.5) | 0.946 | 0.946-1.424 | 0.791 |  |  |  |
| **Marital status** |  |  |  |  |  |  |  |  |
| Not married | 48(1.8) | 2671(98.2) | **Reference** |  |  | **Reference** |  |  |
| Married | 65(1.4) | 4730(98.6) | 1.308 | 0.898-1.905 | 0.162 | 1.303 | 0.763-2.224 | 0.333 |
| **Region of residence** |  |  |  |  |  |  |  |  |
| East | 24(1.5) | 1555(98.5) | **Reference** |  |  | **Reference** |  |  |
| North | 25(1.4) | 1797(98.6) | 1.109 | 0.631-1.950 | 0.718 | 1.167 | 0.656-2.074 | 0.600 |
| Northwest | 8(0.8) | 1018(99.2) | 1.964 | 0.879-4.389 | 0.100 | 1.908 | 0.846-4.302 | 0.119 |
| South | 43(2.3) | 1788(97.7) | 0.642 | 0.388-1.062 | 0.085 | 0.695 | 0.415-1.162 | 0.165 |
| Western | 13(1.0) | 1243(99.0) | 1.476 | 0.748-2.910 | 0.261 | 0.821 | 0.368-1.831 | 0.630 |
| **Level of education** |  |  |  |  |  |  |  |  |
| Secondary | 32(1.2) | 2609(98.8) | **Reference** |  |  | **Reference** |  |  |
| No formal education | 56(1.6) | 3515(98.4) | 0.770 | 0.497-1.192 | 0.241 | 0.624 | 0.351-1.107 | 0.107 |
| Primary | 25(2.5) | 992(97.5) | 0.487 | 0.287-0.825 | 0.008 | 0.531 | 0.300-0.938 | 0.029 |
| Higher | 0 | 285(100) | 19814180 | 0 | 0.994 | 12203543 | 0 | 0.994 |
| **Wealth Indices** |  |  |  |  |  |  |  |  |
| Middle | 17(1.1) | 1514(98.9) | **Reference** |  |  | **Reference** |  |  |
| Poorest | 41(2.7) | 1492(97.3) | 0.409 | 0.231-0.722 | 0.002 | 0.485 | 0.268-0.880 | 0.017 |
| Poorer | 21(1.5) | 1407(98.5) | 0.752 | 0.395-1.432 | 0.386 | 0.778 | 0.404-1.497 | 0.452 |
| Richer | 24(1.5) | 1610(98.5) | 0.753 | 0.403-1.408 | 0.374 | 0.654 | 0.302-1.417 | 0.282 |
| Richest | 10(0.7) | 1378(99.3) | 1.547 | 0.706-3.391 | 0.275 | 1.068 | 0.377-3.026 | 0.902 |
| **Watching television** |  |  |  |  |  |  |  |  |
| No | 96(1.7) | 5529(98.3) | **Reference** |  |  | **Reference** |  |  |
| Yes | 17(0.9) | 1872(99.1) | 1.912 | 1.139-3.211 | 0.014 | 1.385 | 0.744-2.578 | 0.304 |
| **Listens to radio** |  |  |  |  |  |  |  |  |
| No | 74(1.7) | 4298(98.3) | **Reference** |  |  | **Reference** |  |  |
| Yes | 39(1.2) | 3103(98.8) | 1.37 | 0.927-2.204 | 0.114 | 0.902 | 0.585-1.392 | 0.642 |
| **Reading of magazines** |  |  |  |  |  |  |  |  |
| No | 108(1.5) | 6917(98.5) | **Reference** |  |  |  |  |  |
| Yes | 5(1.0) | 484(99.0) | 1.511 | 0.614-3.722 | 0.369 |  |  |  |
| **Smokes cigarettes** |  |  |  |  |  |  |  |  |
| No | 109(1.5) | 7181(98.5) | **Reference** |  |  |  |  |  |
| Yes | 4(1.8) | 220(98.2) | 0.835 | 0.305-2.285 | 0.725 |  |  |  |
| **Alcohol use** |  |  |  |  |  |  |  |  |
| No | 45(1.5) | 3036(98.5) | **Reference** |  |  |  |  |  |
| Yes | 8(1.2) | 659(98.8) | 1.221 | 0.573-2.602 | 0.605 |  |  |  |

**aOR: adjusted Odds Ratio; CI: Confidence Interval; COR: Crude Odds Ratio; SLDHS: Sierra Leone Demographic and Health Survey.**

In Table 2, the determinants of stunting among Sierra Leone women of reproductive age were less likely among women of primary level of education, aOR=0.53,95%CI:0.30-0.94;p=0.029 and those in the poorest wealth index aOR=0.49,95%CI:0.27-0.88; p=0.017.

**Table 3: Prevalence and determinants of underweight among women (15-49 years) in SLDHS-2019.**

| **Variables** | **Under-**  **weight**  **(N=502)**  **(n, %)** | **Normal weight**  **(N=4,974)**  **(n, %)** | **Unadjusted**  **(COR)** | **95% CI** | **p value** | **Adjusted (aOR)** |  |  |
| --- | --- | --- | --- | --- | --- | --- | --- | --- |
|  |  |  |  |  |  |  | **95% CI** | **p value** |
|  |  |  |  |  |  |  |  |  |
|  |  |  |  |  |  |  |  |  |
| **Age groups (years)** | | |  |  |  |  |  |  |
| 20-29 | 106(5.6) | 1,773(94.4) | **Reference** |  |  | **Reference** |  |  |
| 15-19 | 227(16.0) | 1,192(84.0) | 0.314 | 0.246-0.400 | <.001 | 0.457 | 0.335-0.624 | <.001 |
| 30-39 | 97(7.2) | 1,244(92.8) | 0.767 | 0.577-1.019 | 0.068 | 0.746 | 0.536-1.037 | 0.081 |
| 40-49 | 72(8.6) | 765(91.4) | 0.635 | 0.465-0.867 | 0.004 | 0.663 | 0.453-0.972 | 0.035 |
| **Parity** |  |  |  |  |  |  |  |  |
| Never gave birth | 225(14.5) | 1,330(85.5) | **Reference** |  |  | **Reference** |  |  |
| One to four | 182(6.7) | 2537(93.3) | 2.358 | 1.918-2.899 | <.001 | 1.479 | 1.079-2.029 | 0.015 |
| Five and above | 95(7.9) | 1,107(92.1) | 1.971 | 1.531-2.538 | <.001 | 1.362 | 0.876-2.117 | 0.170 |
| **Residence** |  |  |  |  |  |  |  |  |
| Rural | 340(9.7) | 3,156(90.3) | **Reference** |  |  |  |  |  |
| Urban | 162(8.2) | 1,818(91.8) | 1.209 | 0.994-1.470 | 0.057 |  |  |  |
| **Sex of household head** | | | | | | | | |
| Male | 343(8.7) | 3,621(91.3) | **Reference** |  |  | **Reference** |  |  |
| Female | 159(10.5) | 1,353(89.5) | 0.806 | 0.661-0.983 | 0.033 | 0.866 | 0.701-1.071 | 0.186 |
| **Household size** | | | | | | | |  |
| Six and above | 321(9.7) | 2,998(90.3) | **Reference** |  |  |  |  |  |
| Less than six | 181(8.4) | 1,976(91.6) | 1.169 | 0.966-1.415 | 0.109 |  |  |  |
| **Work status** |  |  |  |  |  |  |  |  |
| Not working | 191(11.1) | 1,529(88.9) | **Reference** |  |  | **Reference** |  |  |
| Working | 311(8.3) | 3,445(91.7) | 1.384 | 1.144-1.673 | 0.001 | 1.011 | 0.800-1.277 | 0.928 |
| **Marital status** |  |  |  |  |  |  |  |  |
| Not Married | 270(12.6) | 1,872(87.4) | **Reference** |  |  | **Reference** |  |  |
| Married | 232(7.0) | 3,102(93.0) | 1.928 | 1.603-2.319 | <.001 | 1.251 | 0.936-1.672 | 0.130 |
| **Region of residence** | | |  |  |  |  |  |  |
| East | 96(8.1) | 1,082(91.9) | **Reference** |  |  | **Reference** |  |  |
| North | 153(10.5) | 1,305(89.5) | 0.757 | 0.579-0.989 | 0.041 | 0.765 | 0.581-1.008 | 0.057 |
| Northwest | 73(9.2) | 724(90.8) | 0.88 | 0.640-1.210 | 0.431 | 0.898 | 0.648-1.243 | 0.515 |
| South | 134(10.3) | 1,173(89.7) | 0.777 | 0.590-1.022 | 0.071 | 0.789 | 0.595-1.045 | 0.098 |
| Western | 46(6.2) | 690(93.8) | 1.331 | 0.925-1.916 | 0.777 | 1.248 | 0.823-1.892 | 0.298 |
| **Level of education** | | |  |  |  |  |  |  |
| Secondary | 185(9.5) | 1,755(90.5) | **Reference** |  |  | **Reference** |  |  |
| No formal education | 211(8.1) | 2,399(91.9) | 1.199 | 0.975-1.474 | 0.086 | 0.886 | 0.662-1.186 | 0.417 |
| Primary | 96(12.3) | 686(87.7) | 0.753 | 0.580-0.979 | 0.034 | 0.742 | 0.557-0.989 | 0.042 |
| Higher | 10(6.9) | 134(93.1) | 1.413 | 0.730-2.733 | 0.305 | 0.677 | 0.338-1.357 | 0.272 |
| **Wealth Indices (WI)** |  |  |  |  |  |  |  |  |
| Middle | 121(10.3) | 1,050(89.7) | **Reference** |  |  | **Reference** |  |  |
| Poorest | 104(8.3) | 1,156(91.7) | 1.281 | 0.973-1.666 | 0.078 | 1.236 | 0.929-1.646 | 0.146 |
| Poorer | 120(10.2) | 1,053(89.8) | 1.011 | 0.775-1.320 | 0.935 | 0.935 | 0.711-1.229 | 0.630 |
| Richer | 97(9.1) | 974(90.9) | 1.157 | 0.874-1.533 | 0.309 | 1.150 | 0.850-1.557 | 0.365 |
| Richest | 60(7.5) | 741(92.5) | 1.423 | 1.030-1.967 | 0.032 | 1.158 | 0.782-1.713 | 0.464 |
| **Watching television** | | |  |  |  |  |  |  |
| No | 404(9.5) | 3,851(90.5) | **Reference** |  |  |  |  |  |
| Yes | 98(8.0) | 1,123(92.0) | 1.202 | 0.955-1.514 | 0.117 |  |  |  |
| **Listens to radio** | | | |  |  |  |  |  |
| No | 350(10.4) | 3,007(89.6) | **Reference** |  |  | **Reference** |  |  |
| Yes | 152(7.2) | 1,967(92.8) | 1.506 | 1.235-1.837 | <.001 | 1.407 | 1.136-1.742 | 0.002 |
| **Reading magazines** | | | | |  |  |  |  |
| No | 473(9.1) | 4,698(90.9) | **Reference** |  |  |  |  |  |
| Yes | 29(9.5) | 276(90.5) | 0.958 | 0.646-1.421 | 0.832 |  |  |  |
| **Smokes cigarettes** | | | | | |  |  |  |
| No | 484(9.1) | 4,835(90.9) | **Reference** |  |  |  |  |  |
| Yes | 18(11.5) | 139(88.5) | 0.773 | 0.469-1.274 | 0.313 |  |  |  |
| **Alcohol use** |  |  |  |  |  |  |  |  |
| No | 140(6.7) | 2,005(93.3) | **Reference** |  |  |  |  |  |
| Yes | 35(7.5) | 429(92.5) | 0.856 | 0.582-1.258 | 0.428 |  |  |  |

**aOR: adjusted Odds Ratio; CI: Confidence Interval; COR: Crude Odds Ratio; SLDHS: Sierra Leone Demographic and Health Survey.**

In Table 3, the determinants of underweight among Sierra Leone women was likely among women with parity of one-to- four aOR=1.48,95%CI:1.08-2.03;p=0.015 and those who listened to radios, aOR=1.41,95%CI:1.14-1.74;p=0.002. However, underweight was less likely among age-group of 15-19 years, aOR=0.46,95%CI:0.34-0.62;p<0.001; age-group of 40-49 years, aOR=0.66,95%CI:0.45-0.97;p=0.035, and those with primary level of education, aOR=0.74,95%CI:0.56-0.99;p=0.042.

This population-based study provides valuable insights into the prevalence and correlates of being underweight and stunted among women of reproductive age (15-49 years) in Sierra Leone (Table 1, Figures 1, and 2). The prevalence of being stunted among women (15-49 years) in Sierra Leone at 1.5%, is higher than that reported in the DHS of Kenya (less than 1%) [41], and Uganda (1.3%) [36,42] but lower than Tanzania (less than 3%) [43].

Stunting among women of reproductive age is a significant concern, as it reflects long-term exposure to inadequate nutrition, infection, and environmental stress [44]. The consequences of stunting are far-reaching, particularly for girls and women of reproductive age [45], and the effects are experienced at individual, community, and national levels [46]. It is disturbing to note that an estimated 450 million adult women in developing countries are stunted due to malnutrition during childhood [47]. Thus, addressing stunting among women is crucial for improving maternal and child health outcomes.

**Stunting among women of reproductive age (15-49 years) in Sierra Leone from the 2019 DHS.** Women with primary education were 47% less likelihood of being stunted than those with secondary education. Similarly, women in the poorest wealth index were 51% less likelihood of being stunted compared to those in the middle wealth index. These findings highlight the importance of education and socio-economic status in mitigating the risk of stunting among women. However, no other factors were significantly associated with being stunted in this study population (Table 2).

In contrast, the correlates of being underweight differed from those of stunting (Tables 2 and 3). A parity of one to four and listening to radios were the significant factors associated with being underweight (Table 3). Women with a parity of one to four were 1.48 times more likely of being underweight than those who had never given birth (Table 3). On the other hand, age groups of 15-19 years and 40-49 years, as well as primary education, were less likely of being underweight. These findings may suggest that different factors contribute to being underweight than stunting among women (15-49 years) in Sierra Leone (Table 3) [48].

The underlying reasons for the low likelihood of being stunted and underweight among women with primary education in this study population remain unclear, highlighting the need for in-depth exploration through qualitative research. Conducting qualitative studies would allow for a deeper understanding of the factors and mechanisms contributing to the observed association between primary education and better nutritional outcomes. By delving into women's lived experiences and socio-cultural context, qualitative research can provide valuable insights to unravel the complex dynamics at play. Thus, additional investigation through qualitative research is warranted to understand why primary education emerges as a shielding factor against being stunted and underweight in this study population.

It is interesting to note that women in the poorest wealth index were less likely of being stunted than women in the middle wealth index (Table 2). This finding contradicts many studies in other African countries where stunting is more prevalent among women in the poorest wealth indices [36,42,49].

Studies on stunting among children in Sierra Leone from the same SLDHS-2019 show a high prevalence among children five years and below [50]. However, our findings that women in the reproductive age group (15-49 years) from the same data source (SLDHS-2019) had no likely association with any age group were unique. In comparison, children below five years experienced a high prevalence of stunting (31.6% in rural versus 24.0% in urban areas) [50].

The unique finding in Sierra Leone necessitates further investigation to explore the underlying factors contributing to this difference. It is plausible that low-income households have adopted favorable eating habits and practices, such as consuming locally available foods like “plasas”. “Plasas”, a mixture of green leaves with palm oil and fish, is affordable and highly nutritious. Understanding the dietary choices and affordability of nutritious foods among low-income households could provide valuable insights into the observed findings.

Furthermore, stunting is a chronic condition that begins during the prenatal period and persists through early childhood and adolescence, with the first two years of life being particularly critical [43,49]. Previous studies have highlighted the high prevalence of stunting among women of reproductive age in low-and-middle-income countries, as stunted children often continue to experience stunting into adulthood [50,51]. However, it is essential to note that some individuals who were stunted in childhood overcame these challenges by accessing education, obtaining better employment opportunities, increasing their income, or marrying into higher socio-economic strata. As a result, they may have transitioned from lower to higher wealth indices, indicating the potential for social mobility and improvement in their overall well-being. This socio-economic progress achieved by these women may have played an important role in the observed outcome of low socio-economic status being unlikely of undernutrition in this study population.

In addition, many studies show that improved drinking water was associated with a lower risk of stunting and that improved water was a proxy for less exposure to enteric pathogens [52]. Watanabe and Petri deliberated that environmental enteropathy is a chronic disease caused by continuous exposure to faecally contaminated food and water that does not produce symptoms but contributes to poor physical development [52]. This finding may have been a factor experienced among study populations in other countries but not in Sierra Leone.

These findings on stunting among women in Sierra Leone contrast with another in Uganda, where the population in the Southwestern northeastern (pygmies and Batwa) was naturally shorter compared to the average Ugandan population [53-55]. More to this could be explained by genetic factors, which play a part at individual level, where it is likely that women of reproductive age in Sierra Leone were generally taller because of their genetic makeup [12]. A contrasting scenario was observed in western Uganda among the pygmies and others who were generally shorter than the average Ugandan population [36,53,54]. However, the situation can be determined further by conducting more comprehensive studies on the height profiles of women in Sierra Leone over several decades to determine the changing patterns of women's heights stratified by regions of the country.

Also, one of the insignificant factors of stunting was the age group of 15-19 years, which is linked to an age group with rapid growth, increased activities, and a high need for adequate nutrients (Table 2). The need for adequate nutrients and diet are paramount for growth and development of persons in that age category. Our findings that there were no factors significantly associated with stunting among women in specific age groups and poor household wealth indices were inconsistent with literature from Bangladesh and other countries [55-60].

Genetic predilections and environmental factors mainly determine adult heights. In addition to genetic impacts, incomes, social status, infections, and nutrition have been shown to affect body height in the European population [60]. Also, environmental factors are likely to be more important determinants of height in low-and middle-income countries because environmental stress, including food availability and infections, are higher in those countries compared to high-income countries.

Perkins et al. explained in their reviews that short adult stature in low-and-middle-income countries are mainly because of cumulative net impacts of nutrition associated with diseases and environmental conditions, such as socio-economic status [56].

The correlates of stunting and underweight among women of reproductive age (15-49 years) in Sierra Leone were different and raised our concerns (Tables 2 and 3). Many factors singly or collectively contribute to underweight and stunting, including eating patterns, food types, their availability, infections, diseases, physical activity levels, and sleep routines [5,6]. In addition to social determinants of health, genetics and taking certain medications have been shown to play essential roles in undernutrition in a population [5,6,10,61].

If compared with overweight and obesity the two are mainly caused by food consumption and activities where people gain weight when they eat more calories than they burn through daily activities [61,62]. In addition, environmental factors around us matter in the development of obesity and overweight, just like stunting and underweight [62]. The world around us influences our ability to maintain a healthy weight and lifestyle. That has been seen in many African communities where people who are obese are considered healthy, living a prosperous and fulfilling life, an issue which is admired by women in many African communities [62].

On the other side of the spectrum, some communities have begun to admire smaller size and equate them to successful and healthy lives. In this, several blue-collar workers have begun to reduce their sizes by conducting regular exercises, eating organic foods, fasting, eating fewer fast foods, less snacking, taking fewer salts and sugars, living a less sedentary lifestyle, riding bicycles, or walking to work, sleeping better, avoiding stressful and mental health situations [62].

Perhaps most interesting from this study is that correlates of underweight and stunting among women in Sierra Leone were different, a factor that should be determined through a comprehensive study, unearthing the underlying reasons. This finding contrasts with many studies in the African continent [36,41-43,45].

Chronic effects of malnutrition in early childhood due to inadequate nutrients and unavailability of food are reflected in later life by stunting and other lifelong consequences such as reduced cognitive function, maternal and child health complications, which we did not find in this study population (Table 2).

These correlates of stunting ought to be addressed in Sierra Leonne's women if improvement in maternal and child health indicators are to be achieved soon in this country [63]. Feeding habits, diets, and food availability for young women in Sierra Leone should be prioritized as soon as possible since many young women of reproductive age are affected by stunting and underweight (Tables 2 and 3).

In addition, early childhood nutrition programs (for example, school feeding programs) could be a welcome intervention for school-going female children.

It is worth noting that there is limited literature on stunting among women of reproductive age in Sierra Leone, with most studies focusing on underweight. Therefore, findings of this study contribute to filling this knowledge gap and could be used for setting a proper agenda for the population.

**Strengths and limitations of this study:** This study has several strengths. First, the data quality of this study was assured as the SLDHS-2019 used well-trained field personnel, standardized protocols, and validated tools in data collection processes. Second, this study utilized a nationally representative sample population of women in the reproductive age of 15-49 years. As a result, the study's findings can be generalizable to the target population in Sierra Leone and many low-to-middle-income countries in the African continent. Third, using validated tools and calibrated instruments by the SLDHS-2019, the generated estimates are more robust than other studies in Sierra Leone's context. In addition, we used data with a large sample size, which was collected, entered, and cleaned by a team of well-trained and highly experienced scientists, thus limiting mistakes in the dataset used in the analysis. Finally, as we used the concentration index, these findings are more robust in predicting socio-economic inequalities among the study population.

However, this study had limitations that warrant further discussion. First, the SLDHS-2019 was a cross-sectional survey. As a result, we cannot establish a sequential relationship between explanatory and outcome variables. Second, due to the absence of some crucial data, several significant variables, such as food security and dietary diversity, could not be included in the final model for the analysis. Third, the SLDHS-2019 did not collect individual incomes and expenditures but household data. It used a wealth index as a proxy indicator for household wealth. Fourth, SLDHS collected data only on 15–49-year-old women of reproductive age in Sierra Leone. With the current changes in adolescents' reproductive actions and behaviors, there are children less than 15 years old who have gone through an entire cycle of reproduction. As a result, the distribution of undernutrition among women below and beyond this age group (15-49 years) was not factored in the analysis. Finally, most data on predictors of undernutrition were based on self-reported information. They were not verified through records, which risks socially acceptable answers, hence social desirability bias in this result.

**Generalizability of results:** Results from this study can be generalized to women of reproductive age (15-49 years) in resource settings in low-to-middle-income countries.

**Conclusion.** The prevalence of underweight and stunting among women of reproductive age (15-49 years) in Sierra Leone was lower compared to regional and world data. This study highlights similarities and differences in this population's prevalence and correlates of undernutrition. Underweight and stunting were less likely in women with primary education, while parity of one-to-four and listening to radios were significantly associated with underweight. Further trend studies using DHS data from 2010, 2014, and 2019 are warranted to understand the dynamics of undernutrition in Sierra Leone.

Furthermore, there is a need to improve the social determinants of health in Sierra Leone in women of reproductive age, including school feeding programs among children and adolescents.

In addition, it is essential to note that this study's findings have important implications for addressing maternal and child health in Sierra Leone. The identified correlates of stunting and underweight should be addressed through targeted interventions. Improving feeding habits, ensuring dietary diversity, and addressing food availability for young women in Sierra Leone should be prioritized. Early childhood nutrition programs, such as school feeding programs, could be effective interventions for improving the nutritional status of school-going female children.

In summary, this study provides valuable insights into the prevalence and correlates of underweight and stunting among women of reproductive age in Sierra Leone. The findings highlight the importance of education, socio-economic status, and environmental factors in influencing nutritional outcomes. Addressing the correlates of stunting and underweight among women is essential for improving maternal and child health indicators in Sierra Leone. Further research is needed to explore the underlying reasons for the observed differences in correlates between stunting and underweight and to develop targeted interventions to alleviate these nutritional challenges.

**Abbreviations**

aOR=adjusted Odds Ratio; BMI=Body Mass Index; CI=Confidence Intervals; COR=Crude Odds Ratio; DHS=Demographic Health Survey; EA=Enumeration Areas; SD=Standard Deviation; SLDHS=Sierra Leone Demographic Health Survey; SDGs=Sustainable Development Goals; USAID=United States of America Agency for International Development; WHO=World Health Organization.

**Declarations**

**Acknowledgment:** We acknowledge with many thanks for the assistance from 2019 SLDHS for the information obtained.

**Availability of data and material:** All datasets supporting this article's conclusion are within this article and have been submitted as supporting information files for this manuscript.

**Competing interests:** All authors declare no conflict of interest.

**Financial Disclosure:** The authors received no specific funding for this work.

**Authors' contributions:** DLK, JA, and FWDO designed the study. JA, ENI, FWDO, and DLK supervised data management. ENI, JA, MAT, LN, EO, NOA, OE, and DLK analyzed and interpreted data. NOA, ENI, FWDO, JA, MAT, EO, LN, KK, AN, OE, JBM, RK, and DLK wrote and revised the manuscript. All the authors approved the revised manuscript.

**Authors' Information:** Dr. Nelson Onira Alema is a Lecturer in the Department of Anatomy, Faculty of Medicine, Gulu University, Gulu City, Uganda; Dr. Eric Nzirakaindi Ikoona (ENI) is a Technical Director at ICAP at the University of Columbia, Sierra Leone; Dr. Mame Awa Toure (MAT) is at ICAP at the University of Columbia, Freetown, Sierra Leone; Dr. Oliver Eleeza (OE) is an associate director strategic information, ICAP at Columbia University, Sierra Leone; Dr. John Bosco Matovu (JBM) is at ICAP at the University of Columbia, Nairobi, Kenya; Dr. Amon Njenga (AN) is at ICAP at the University of Columbia, Freetown, Sierra Leone; Dr. Lucy Namulemo (LN) is at Foothills Community Based interventions, Monticello, Kentucky, USA; Dr. Ronald Kaluya (RK) is at Uganda Counseling and Support Services, Kampala, Uganda; Dr. Kassim Kamara (KK) is at the Directorate of health security and emergencies, Ministry of Health and Sanitation, Sierra Leone; Dr. Freddy Wathum Drinkwater Oyat (FWDO) is a senior physician, a public health specialist, and a member of Uganda Medical Association, UMA-Acholi branch, Gulu City, Uganda; Dr. Judith Aloyo (JA) is the Deputy Chief of Party at the Rhites-N, Acholi, Gulu City, Uganda; Dr. Emmanuel Olal (EO) is a Physician and a public health specialist at Yotkom Medical Centre, Kitgum, Uganda; Prof. David Lagoro Kitara (DLK) is a Takemi fellow of Harvard University and a Professor at Gulu University, Faculty of Medicine, Department of Surgery, Gulu City, Uganda.

**References**

1. Leslie Elder, Elizabeth Ransom. Nutrition of women and adolescent girls: Why it matters. <https://www.prb.org/resources/nutrition-of-women-and-adolescent-girls-why-it-matters/>.
2. Sun Movement. Scaling up nutrition: a framework for action. Sun Movement [homepage on the Internet]. 2011. http://scalingupnutrition.org/wp-content/uploads/ pdf/SUN_Framework.pdf.
3. Rotimi C, Okosun I, Johnson L. The distribution of chronic energy deficiency among adult Nigerian men and women. Eur J Clin Nutr.1999;53(9):734-739.
4. World Health Organization. Physical status: the use and interpretation of anthropometry. Geneva: WHO, 1995.
5. Merchant KM, Kurtz KM. Women’s nutrition through the life cycle: social and biological vulnerabilities. In: Marge Koblinsky, Judith Timyan and Jill Gay, editors. The health of women: a global perspective. Boulder: Westview Press, 1993;63-90.
6. Bitew FH, Telake DS. Undernutrition among women in Ethiopia: rural-urban disparity. Calverton: ICF Macro, 2010.
7. Stewart CP, Iannotti L, Dewey KG, Michaelsen KF, Onyango AW. Contextualizing complementary feeding in a broader framework for stunting prevention. Matern Child Nutr. 2013;9(suppl 2):27-45.
8. Prado EL, Dewey KG. Nutrition and brain development in early life. Nutr Rev. 2014;72:267-284.
9. Grantham-McGregor S, Cheung YB, Cueto S, Glewwe P, Richter L, Strupp B. Developmental potential in the first 5 years for children in developing countries. Lancet. 2007;369:60-70.
10. [Ashraf Soliman](https://pubmed.ncbi.nlm.nih.gov/?term=Soliman%20A%5BAuthor%5D), [Vincenzo De Sanctis](https://pubmed.ncbi.nlm.nih.gov/?term=De%20Sanctis%20V%5BAuthor%5D), [Nada Alaaraj](https://pubmed.ncbi.nlm.nih.gov/?term=Alaaraj%20N%5BAuthor%5D), [Shayma Ahmed](https://pubmed.ncbi.nlm.nih.gov/?term=Ahmed%20S%5BAuthor%5D), [Fawziya Alyafei](https://pubmed.ncbi.nlm.nih.gov/?term=Alyafei%20F%5BAuthor%5D), [Noor Hamed](https://pubmed.ncbi.nlm.nih.gov/?term=Hamed%20N%5BAuthor%5D), *et al.,* Early and Long-term Consequences of Nutritional Stunting: From Childhood to Adulthood. [Acta Biomed.](https://www.ncbi.nlm.nih.gov/pmc/articles/PMC7975963/) 2021;92(1): e2021168.
11. Shafique S, Akhter N, Stallkamp G. Trends of under- and overweight among rural and urban poor women indicate the double burden of malnutrition in Bangladesh. Int J Epidemiol. 2007; 36:449–457.
12. Mallia T, Grech A, Hili A. Genetic determinants of low birth weight. Minerva Ginecol. 2017; 69:631–643.
13. Dahlui M, Azahar N, Oche OM. Risk factors for low birth weight in Nigeria: evidence from the 2013 Nigeria Demographic and Health Survey. Glob Health Action 9. 2016;28822.
14. Nnam NM. Improving maternal nutrition for better pregnancy outcomes. Proc Nutr Soc. 2017;74:454-459.
15. Khan MN, Rahman MM, Shariff AA. Maternal undernutrition and excessive body weight and risk of birth and health outcomes. Arch Public Health.2017;75:12.
16. Xu H, Shatenstein B, Luo Z-C. Role of nutrition in the risk of preeclampsia. Nutr Clin Care. 2009; 67:639-657.
17. Chopra M, Galbraith S & Darnton-Hill. A global response to a global problem: the epidemic of overnutrition. Bull World Health Organ. 2002; 80:952–958.
18. Ng M, Fleming T, Robinson M. Global, regional, and national prevalence of overweight and obesity in children and adults 1980–2013: a systematic analysis. Lancet. 2014; 384:766–781.
19. Zahangir MS, Hasan MM, Richardson A. Malnutrition, and non-communicable diseases among Bangladeshi women: an urban–rural comparison. Nutr Diabetes. 2017; 7, e250.
20. Ly KA, Ton TGN, Ngo QV. Double burden: a cross-sectional survey assessing factors associated with underweight and overweight status in Danang, Vietnam. BMC Public Health.2013;13:35.
21. Fenn B, Bulti AT, Nduna T. An evaluation of an operations research project to reduce childhood stunting in a food-insecure area in Ethiopia. Public Health Nutr.2012;15:1746–1754.
22. World Health Organization, UNICEF & US Agency for International Development (2015) Improving Nutrition Outcomes with Better Water, Sanitation and Hygiene: Practical Solutions for Policy and Programmes. Geneva: WHO. 2015.
23. Harris-Fry H, Azad K, Kuddus A. Socioeconomic determinants of household food security and women’s dietary diversity in rural Bangladesh: a cross-sectional study. J Health Popul Nutr. 2015;33:12.
24. Islam A, Islam N, Bharati P. Socio-economic and demographic factors influencing nutritional status among early childbearing young mothers in Bangladesh. BMC Women’s Health. 2016;16:58.
25. Hossain MG, Bharati P, Aik SAW. Body mass index of married Bangladeshi women: trends and association with socio-demographic factors. J Biosoc Sci. 2012;44:385-399.
26. Bhuiya A & Mostafa G. Levels and differentials in weight, height, and body mass index among mothers in a rural area of Bangladesh. J Biosoc Sci. 1993;25:31-38.
27. Baqui AH, Arifeen SE, Amin S. Levels and correlates of maternal nutrition status in urban Bangladesh. Eur J Clin Nutr. 1994;48:349–357.
28. Ahsan KZ, Arifeen SE, Al-Mamun MA. Effects of individual, household, and community characteristics on child nutritional status in the slums of urban Bangladesh. Arch Public Health.2017;75:9.
29. Statistics Sierra Leone-StatsSL, ICF. Sierra Leone: Demographic and health survey 2019. 2020. https://www.dhsprogram.com/pubs/pdf/FR365/FR365. pdf.
30. World Food Program (WFP). State of Food Security in Sierra Leone 2015 Comprehensive Food Security and Vulnerability Analysis Data collected September - October 2015. 2015 Sierra Leone CFSV. https://efaidnbmnnnibpcajpcglclefindmkaj/https://documents.wfp.org/stellent/groups/public/documents/ena/wfp288316.pdf?iframe.
31. Worldometer. The population of Sierra Leone. 2022. https://www.worldometers.info/world-population/sierra-leone-population/
32. Sierra Leone NHSSP. National Health Sector Strategic Plan 2017 – 2021. chrome-extension://efaidnbmnnnibpcajpcglclefindmkaj/https://extranet.who.int/countryplanningcycles/sites/default/files/planning_cycle_repository/sierra_leone/sierra_leone_nhssp_2017-21_final_sept2017.pdf
33. World Bank (WB). Microdata in Sierra Leone, 2019. Demographic and Health Survey 2019. Microdata Library. 2019. https://microdata.worldbank.org/index.php/catalog/3826.
34. Statistics Sierra Leone (Stats SL) and ICF. 2020. Sierra Leone Demographic and Health Survey 2019. Freetown, Sierra Leone, Rockville, Maryland, USA: Stats SL and ICF. 2020. chrome-extension://efaidnbmnnnibpcajpcglclefindmkaj/https://dhsprogram.com/pubs/pdf/FR365/FR365.pdf
35. Pamela Okot Atim, Smart Geoffrey Okot, Eric Nzirakaindi Ikoona, Lucy Namulemo, Judith Aloyo, David Lagoro Kitara, *et al.* Factors and prevalence of undernutrition among women of reproductive age (15-49 years) in Sierra Leone. A secondary data analysis of the demographic health survey of 2019. Research Square. 2023. DOI: <https://doi.org/10.21203/rs.3.rs-3101722/v1>.
36. Sserwanja Q, Mukunya D, Habumugisha T, Mutisya LM, Tuke R, Olal E. Factors associated with undernutrition among 20-to 49-year-old women in Uganda: a secondary analysis of the Uganda demographic health survey 2016. BMC Public Health. 2020;20:1644.
37. Zambia Statistics Agency (ZSA), Ministry of Health (MOH). University Teaching Hospital Virology Laboratory – (UTH-VL), ICF. Zambia demographic and health survey. 2018. https://www.dhsprogram.com/pubs/pdf/ FR361/FR361.pdf.
38. Agbadi P, Eunice TT, Akosua AF, Owusu S. Complex samples logistic regression analysis of predictors of the current use of modern contraceptive among married or in-union women in Sierra Leone: insight from the 2013 demographic and health survey. PLoS One. 2020;15:e0231630.
39. Zou D, Lloyd JEV, Baumbusch JL. Using SPSS to analyze complex survey data: a primer. J Mod Appl Stat Methods. 2020;18:2-22.
40. Croft Trevor N, Aileen MJM, Courtney KA. Guide to DHS Statistics. ICF; 2018.
41. Kenya National Bureau of Statistics, Ministry of Health/Kenya, National AIDS Control Council/Kenya, Kenya Medical Research Institute, Population NCf, Development/Kenya: Kenya Demographic and Health Survey 2014. Rockville; 2015.
42. Quraish Sserwanja. Socio-economic determinants of undernutrition among women of reproductive age in Uganda: a secondary analysis of the 2016 Uganda demographic health survey. UPPSALA UNIVERSITET. 2019;1-49. [*http://www.diva-portal.org/smash/get/diva2:1367320/FULLTEXT01.pdf*](http://www.diva-portal.org/smash/get/diva2:1367320/FULLTEXT01.pdf)*.*
43. Ministry of Health CD, Gender, Elderly, Children - MoHCDGEC/Tanzania Mainland, Ministry of Health - MoH/Zanzibar, National Bureau of Statistics - NBS/Tanzania, Office of Chief Government Statistician - OCGS/Zanzibar, ICF: Tanzania Demographic and Health Survey and Malaria Indicator Survey 2015–2016. Dar es Salaam: MoHCDGEC, MoH, NBS, OCGS, and ICF; 2016.
44. Christian P, Smith ER. Adolescent Undernutrition: Global Burden, Physiology, and Nutritional Risks. Annals of Nutrition and Metabolism. 2018;72(4):316-28.
45. Melaku YA, Zello GA, Gill TK, Adams RJ, Shi Z. Prevalence, and factors associated with stunting and thinness among adolescent students in Northern Ethiopia: a comparison to World Health Organization standards. Archives of Public Health. 2015;73(1):44.
46. Reinhardt K, Fanzo J. Addressing Chronic Malnutrition through Multi-Sectoral, Sustainable Approaches: A Review of the Causes and Consequences. Frontiers in nutrition. 2014;1:13.
47. Haseen F. Malnutrition among ultra poor women in Bangladesh: Malnutrition among Bangladeshi Women in Ultra Poor Households: Prevalence and Determinants. Germany: LAP Lambert Academic Publishing, 2010;52-3.
48. Eric Nzirakaindi Ikoona, Lucy Namulemo, Ronald Kaluya, Freddy Wathum Drinkwater Oyat, Judith Aloyo, David Lagoro Kitara. Prevalence and factors associated with underweight among 15–49-year-old women in Sierra Leone: A secondary Data Analysis of Sierra Leone Demographic Health Survey of 2019. BMC Women’s Health. 2023;23:192. [*https://doi.org/10.1186/s12905-023-02358-4*](https://doi.org/10.1186/s12905-023-02358-4)*.*
49. Negash WD, Fetene SM, Shewarega ES*.* Multilevel analysis of undernutrition and associated factors among adolescent girls and young women in Ethiopia. BMC Nutr. 2022;8:104. https://doi.org/10.1186/s40795-022-00603-x.
50. Sserwanja Q, Kamara K, Mutisya LM, Musaba MW, Ziaei S. Rural and Urban Correlates of Stunting Among Under-Five Children in Sierra Leone: A 2019 Nationwide Cross-Sectional Survey. Nutr Metab Insights. 2021;14:11786388211047056. doi:10.1177/11786388211047056.
51. [Cesar G Victora](https://pubmed.ncbi.nlm.nih.gov/?term=Victora%20CG%5BAuthor%5D), [Parul Christian](https://pubmed.ncbi.nlm.nih.gov/?term=Christian%20P%5BAuthor%5D), [Luis Paulo Vidaletti](https://pubmed.ncbi.nlm.nih.gov/?term=Vidaletti%20LP%5BAuthor%5D), [Giovanna Gatica-Domínguez](https://pubmed.ncbi.nlm.nih.gov/?term=Gatica-Dom%C3%ADnguez%20G%5BAuthor%5D), [Purnima Menon](https://pubmed.ncbi.nlm.nih.gov/?term=Menon%20P%5BAuthor%5D), and [Robert E Black](https://pubmed.ncbi.nlm.nih.gov/?term=Black%20RE%5BAuthor%5D). Revisiting maternal and child undernutrition in low-income and middle-income countries: variable progress towards an unfinished agenda. [Lancet.2021;397(10282):1388–1399.](https://www.ncbi.nlm.nih.gov/entrez/eutils/elink.fcgi?dbfrom=pubmed&retmode=ref&cmd=prlinks&id=33691094) doi: [10.1016/S0140-6736(21)00394-9](https://doi.org/10.1016%2FS0140-6736(21)00394-9).
52. Watanabe K & Petri WA Jr. Environmental enteropathy: elusive but significant subclinical abnormalities in developing countries*.* EBioMedicine. 2016;**10:25**–32.
53. Clark S, Berrang-Ford L, Lwasa S. The burden and determinants of self-reported acute gastrointestinal illness in an Indigenous Batwa Pygmy population in southwestern Uganda. Epidemiol Infect. 2015;143(11):2287–98.
54. Patterson K, Berrang-Ford L, Lwasa S, Namanya DB, Ford J, Twebaze F, *et al.* Seasonal variation of food security among the Batwa of Kanungu, Uganda. Public Health Nutr. 2017;20(1):1–11.
55. Bogin B Scheffler C & Hermanussen M. Global effects of income and income inequality on adult height and sexual dimorphism in height. Am J Hum Biol. 2017; 29.e22980.
56. Perkins JM, Subramanian SV, Davey Smith G*.* Adult height, nutrition, and population health. Nutr Rev. 2016;74:149-165.
57. Silventoinen K. Determinants of variation in adult body height. J Biosoc Sci. 2003;35:263–285.
58. de Oliveira VH & Quintana-Domeque C. Early-life environment and adult stature in Brazil: an analysis for cohorts born between 1950 and 1980. Econ Hum Biol. 2014; 15:67–80.
59. National Institute of Population Research and Training, Mitra, and Associates, & ICF International (2014) Bangladesh Demographic and Health Survey 2014. Dhaka and Rockville, MD: NIPORT, Mitra and Associates, and ICF International.
60. Fudvoye J & Parent AS. Secular trends in growth. Ann Endocrinol (Paris). 2017;78:88-91.
61. Ken Maleta. Undernutrition. Malawi Medical Journal; 2006;18(4):189-205.
62. Eric Nzirakaindi Ikoona, Mame Awa Toure, Kassim Kamara, Freddy Wathum Drinkwater Oyat, Judith Aloyo, David Lagoro Kitara, *et al.* Double Burden of Malnutrition Among Women in Reproductive Age (15-49 years) in Sierra Leone: A Secondary Data Analysis of the Demographic Health Survey of 2019 (SLDH-2019). Research Square. 2022. DOI: [10.21203/rs.3.rs-1992723/v1](http://t.researchsquare.com/track/click/31114617/doi.org?p=eyJzIjoiU2VKV3ktenphTXJOWkZib25nVk5QUjlIVlZFIiwidiI6MSwicCI6IntcInVcIjozMTExNDYxNyxcInZcIjoxLFwidXJsXCI6XCJodHRwczpcXFwvXFxcL2RvaS5vcmdcXFwvMTAuMjEyMDNcXFwvcnMuMy5ycy0xOTkyNzIzXFxcL3YxXCIsXCJpZFwiOlwiYmJkYjhhM2ZlM2IwNGI1ZGEwYjE2OGNiNjE0Njc0YzhcIixcInVybF9pZHNcIjpbXCIzZGQwZTAxMmExMzk4NDhkNTAzYjI4ZTBiZmU1Y2QxMDcxNzhlZTgwXCJdfSJ9).
63. Survey Quraish Sserwanja, Kassim Kamara, Linet M Mutisya, Milton W Musaba and Shirin Ziaei. Rural and Urban Correlates of Stunting Among Under Five Children in Sierra Leone: A 2019 Nationwide Cross-Sectional. Nutrition and Metabolic Insights. 2021;4:1–10.
