# Supplementary material for: Prevalence and factors associated with undernutrition among 15–49-year-old women in Sierra Leone: A secondary data analysis of Sierra Leone Demographic Health Survey of 2019
Source: PLoS One. 2024 Nov 11;19(11):e0311845. doi: 10.1371/journal.pone.0311845 (PMC11554195; doi:10.1371/journal.pone.0311845)
Supplement: Attachment — Submitted filename: REVIEWERS COMMENT-PONE-D-23-29763.docx [file pone.0311845.s004.docx]

**REVIEWERS COMMENT-PONE-D-23-29763**

Malnutrition, characterized by deficiencies in calories, protein, vitamins, minerals, poor health, and social conditions, poses a significant health challenge for millions of women and adolescent girls worldwide. Adequate nutrition is crucial for women's overall health and has far-reaching implications for the well-being of their children. Children born to malnourished women are at higher risk of cognitive impairments, stunted growth, increased susceptibility to infections, and elevated morbidity and mortality rates throughout their lives. Undernutrition remains a pressing global health issue, encompassing being underweight, wasted, stunted, and with deficiencies in essential minerals and vitamins. Research indicates that women with a body mass index (BMI) below 18.5kg/m^2^ in developing countries face an escalating mortality risk and heightened vulnerability to illnesses. Despite the significance of understanding maternal nutritional status, limited research has been conducted in Sierra Leone, often focusing solely on malnutrition determinants in young children and adolescents. The present study addresses this research gap by investigating the risk factors of undernutrition among women of reproductive age (15-49 years) in Sierra Leone, utilizing data from the Sierra Leone Demographic Health Survey (SLDHS-2019). Findings of this study hold essential policy implications from a global health perspective and specifically for Sierra Leone, aiding in monitoring progress toward sustainable development goals (SDGs) and regional nutrition strategies. Moreover, the study can guide the allocation of limited resources by the Government and health stakeholders to improve the nutritional and health status of women and infants in Sierra Leone.

**Major comment:** the study was well researched, detailed and scientific. The methodology, analysis and interpretation were well written and discussed, hence the manuscript is good for publication. However, few corrections are needed.

**Minor comments**:

The WHO reference criteria for classification of BMI must be provided-Page 6

Page 8-Outcome variables; the WHO reference must be provided

The ethical approval for the protocol (ID number) must be provided on page 9

The first line on page 20 had no reference

References: many of the earlier references on pages 24 and 25 are obsolete.
