# Supplementary material for: Prevalence and factors associated with undernutrition among 15–49-year-old women in Sierra Leone: A secondary data analysis of Sierra Leone Demographic Health Survey of 2019
Source: PLoS One. 2024 Nov 11;19(11):e0311845. doi: 10.1371/journal.pone.0311845 (PMC11554195; doi:10.1371/journal.pone.0311845)
Supplement: Attachment — Submitted filename: Point by point response to reviewers.docx [file pone.0311845.s005.docx]

**A point-by-point response to reviewers.**

**Prevalence and factors associated with undernutrition among 15–49-year-old women in Sierra Leone: A secondary data analysis of Sierra Leone Demographic Health Survey of 2019.**

**On behalf of the authors, I wish to thank the reviewers for the comprehensive review of our manuscript. We are certain it has helped us to improve the quality of our manuscript. Your reviews are well appreciated.**

**Reviewer 1: The point-by-point response.**

1. The WHO reference criteria for the classification of BMI must be provided 6. We agree with your suggestion and on page 6 we have included this. The Body Mass Index (BMI) of respondents was calculated in kg/m^2^ using weights (in kilograms) and heights (meters) of women of reproductive age (15–49 years) and classified according to WHO criteria as underweight (<18.5kg/m^2^), normal weight (18.5–24.9kg/m^2^), overweight (25.0–29.9kg/m^2^), obesity (≥30.0kg/m^2^ and ≤50.0kg/m^2^), and overnutrition (≥25.0kg/m^2^ and ≤50.0kg/m^2^) [36].

[36] World Health Organization (WHO). Malnutrition. 2023. <https://www.who.int/news-room/fact-sheets/detail/malnutrition>.

2. Page 8-Outcome variables; the WHO reference must be provided. We agree with your suggestion and thank you very much. Here it is. The first outcome variable for this study was stunting among women (15-49 years). It was defined as heights of <145cm ± Standard Deviations (SD) from the median value set by the World Health Organization (WHO) [37,38].

[37] WHO. Global nutrition targets 2015: stunting policy brief (WHO/NMH/NHD/14.3). World Health Organization: Geneva. 2014a.

[38] Sserwanja Q, Mukunya D, Habumugisha T, Mutisya LM, Tuke R, Olal E. Factors associated with undernutrition among 20-to 49-year-old women in Uganda: a secondary analysis of the Uganda demographic health survey 2016. BMC Public Health. 2020;20:1644.

3. The ethical approval for the protocol (ID number) must be provided on page 9. Yes, we agree that we should provide the ethical approval number for this survey. We acknowledge this as an important requirement for this publication. However, we are requesting you the reviewer that when we were given the authorization to use the Sierra Leone DHS data, it was without the IRB approval number which was conducted by the Sierra Leone Ethics and Scientific Review Committee and the IRB of ICF. For more information, I wish to refer you to these documents and the letter of authorization to use this DHS data from 2019. <https://dhsprogram.com/pubs/pdf/FR365/FR365.pdf> and Quraish Sserwanja, Kassim Kamara, Linet M Mutisya, Milton W Musaba and Shirin Ziaei. Rural and Urban Correlates of Stunting Among Under Five Children in Sierra Leone: A 2019 Nationwide Cross-Sectional. Nutrition and Metabolic Insights. 2021;4:1–10. We contacted the ICF program manager for the IRB and have not yet sent it to us. We therefore request you to allow this manuscript to proceed as this was an international survey that has been regularly conducted across countries and there have been several publications that have been made from secondary data analysis of DHS data.
4. The first line on page 20 had no reference. We agree with your review comment on page 20. We have reviewed it and included the citation and reference. The reference is [54]. [54] Watanabe K & Petri WA Jr. Environmental enteropathy: elusive but significant subclinical abnormalities in developing countries*.* EBioMedicine. 2016;**10:25**–32.
5. References: many earlier references on pages 24 and 25 are obsolete. We agree to your review comments and have accordingly revised references on pages 24 and 25 and are here for you to review our work. Overall, we are grateful for your review comments, and thank you very much.

**Reviewer 2:**

Comments for the project entitled “Prevalence and factors associated with undernutrition among 15–49-year-old women in Sierra Leone: A secondary data analysis of Sierra Leone Demographic Health Survey of 2019”:

1. The whole study was based on only four data sets, BMI of reproductive age group females, Wealth proxy index, and educational status and parity. The author associated underweight with the level of education and wealth index. However, Stunting is a complex condition and is dependent upon many factors like genetic factors, hookworm infection, anemia, vitamin D deficiency, Diet and protein intake, diarrheal diseases, vaccination, age of marriage, etc. The study lacks data on nutritional status, infection history, psycho-social development status, etc. Thus in the absence of all such data, the interpretation of ‘ lower prevalence of underweight and stunting among reproductive age women in Sierra Leone’ may not be correct and needs stronger support for such a conclusion. **Response: Thank you reviewer for your comments. We agree that stunting is a complex condition and dependent on many factors as you rightly stated. We have reviewed your other comments and asked ourselves many questions and seeking the most needed answers to your questions. One of which was that we were conducting a secondary data analysis which has been used internationally for many years. It is not possible that we could assess all the factors that determine stunting in one setting but our findings could open more opportunities for further research in this area. We, the authors were persuaded that this was a national survey conducted by the DHS project of Sierra Leone and that the sixteen variables selected were found to have links to undernutrition in the study population.**
2. The Author mentioned (in the result) that 7514 women of reproductive age group participated in the study- which gives a wrong impression that it is an active surveillance, whereas it is a secondary data analysis and data of 7514 participants taken from the 2019 demographic health survey. **Response:** Thank you for your correct observation. We agree to your fair comments. This was indeed a secondary data analysis that was obtained from a cross-sectional study of the 2019 DHS Sierra Leone data. We have corrected this in the revised manuscript. We acknowledge the mistake and apologize for the errors caused.
3. Out of 15,934 women, the selection of 7514 is not properly justified, the author mentioned that those who responded and were within the reproductive age group and have data of BMI, etc were selected. But did not provide a flow chart for selection. Thus selection bias cannot be ruled out. Rather a detailed flow chart of the total participants of the survey ( n=XX), eligible women (mentioning eligibility criteria) ( n=xx), not eligible( n=xxx) and why, total sample available for the study( n=XX), etc. may help to understand the selection process. **Response: Thank you for your comments and very informative. We have revised the manuscript and provided the flowchart to show participants and their eligibility criteria.**
4. As per the WHO definition of stunning “height –for age of children below 5 years is more than two standard deviations below the WHO’s child growth standards median”. Although the author mentioned WHO criteria, but it seems not followed properly as they calculated 113/7514 ( 15-49 years ago) as stunning based on weight and height only. **Response: Thank you for your very informative comments. We have revised the manuscript and provided the flowchart to show stunting among participants. This study was on women of reproductive age (15-49 years) in Sierra Leone in 2019 using DHS data. We have in our discussions tried to relate the prevalence of stunting and other forms of malnutrition in children below 5 years and women of reproductive age from the same DHS datasets. Otherwise, thank you for your review comments because this has enriched our revised manuscript.**
5. The control sample is missing. The male of the same age group would help to understand the claim of undernutrition and stunning. Also, analysis of 0-5 years of age group data may help to support the conclusion. **Response: Thank you for your comments. We have revised the manuscript and provided the flowchart to show the selection criteria for participants. This study was on women of reproductive age (15-49 years) in Sierra Leone in 2019 using DHS data. We have in our discussions tried to relate the prevalence of stunting and other forms of malnutrition in children below 5 years and women of reproductive age from the same DHS datasets. Analysis of men’s data did not yield any helpful information and it did not correspond to the pattern in women of reproductive age. So, we chose not to include it in this manuscript. Otherwise, thank you for the useful comments which have helped us improve our revised manuscript.**
6. The conclusion of the study could not be supported by the required data set. **Response: Thank you for your comments. We have reviewed your comments and have found that our conclusion describes what we obtained from the 2019 DHS Sierra Leone. Our conclusions are based on the analysis findings between the independent and dependent variables. In this case, we wish to request that our conclusion be maintained as it is in the revised manuscript.**

**Reviewer 3:**

**Thank you for your valuable comments. We have included all the revisions in the revised manuscript.**

**Reviewer**: The table shows people with undernutrition are less likely to listen to the radio. please check Table 3, interpret, and explain. Also, mention if there is any possible ecological fallacy. please mention this in the discussion.

**Response: We determined that women who listened to radios were more likely to suffer undernutrition than those who did not (OR=1.407(1.136-1.742); p=0.002). We have explained the possible ecological fallacy in the discussion section.**
